# Middle ear innovation in Early Cretaceous eutherian mammals

**DOI:** 10.1038/s41467-023-42606-7

**Published:** 2023-10-26

**Authors:** Haibing Wang, Yuanqing Wang

**Affiliations:** 1 Key Laboratory of Vertebrate Evolution and Human Origins of Chinese Academy of Sciences, Institute of Vertebrate Paleontology and Paleoanthropology, Chinese Academy of Sciences, 100044 Beijing, China; 2 College of Earth and Planetary Sciences, University of Chinese Academy of Sciences, 100049 Beijing, China

**Keywords:** Palaeontology, Evolutionary developmental biology

## Abstract

The middle ear ossicles in modern mammals are repurposed from postdentary bones in non-mammalian cynodonts. Recent discoveries by palaeontological and embryonic studies have developed different models for the middle ear evolution in mammaliaforms. However, little is known about the evolutionary scenario of the middle ear in early therians. Here we report a detached middle ear preserved in a new eutherian mammal from the Early Cretaceous Jehol Biota. The well-preserved articulation of the malleus and incus suggest that the saddle-shaped incudomallear joint is a major apomorphy of Early Cretaceous eutherians. By contrast to the distinct saddle-like incudomallear articulation in therians, differences between the overlapping versus the half-overlapping incudomallear joints in monotremes and stem mammals would be relatively minor. The middle ear belongs to the microtype by definition, indicating its adaptation to high-frequency hearing. Current evidence indicates that significant evolutionary innovations of the middle ear in modern therians evolved in Early Cretaceous.

## Abstract (web summary)

The evolution of the middle ear in early therians is unclear. Here, the authors report a reconstructed, detached middle ear in a eutherian mammal from the Early Cretaceous Jehol Biota, suggesting independent decoupling of hearing and chewing apparatuses.

## Introduction

Mammals are one of the most successful groups of living creatures with tremendous diversity of morphology and physiologic capability to dominate diverse environments^1^. They possess a broad auditory spectrum and sensitive hearing, particularly for high-frequency sounds, featured by a unique ossicular chain in the middle ear among the hearing organs of tetrapods, a mechanical apparatus that transmits airborne vibrations to the inner ear^2–4^. The characters of the malleus–incus configuration and the stapes are generally conserved in the major extant mammalian groups, despite the variations of the ear bone characters between species^2,5^. Furthermore, the mammalian middle ear is one of the most valuable sources for tracing the evolution of mammals from their “reptilian ancestors”, as palaeontological evidence indicates that the middle ear bones in extant mammals are repurposed from the postdentary bones, quadrate, and columella in nonmammalian cynodonts^6^. It is widely accepted that there are three evolutionary and phylogenetic stages in the evolution of the mammalian middle ear, the postdentary-attached middle ear (PAME), the Meckelian-attached middle ear (MAME), and the detached middle ear (DME)^7–9^. In addition, the evidence for embryonic development of the middle ear and mandible in living mammals is consistent with the evolutionary sequence of the mammalian middle ear suggested by fossil discoveries of Mesozoic mammaliaforms, from *Morganucodon* of the Late Triassic-Early Jurassic to later “transitional” mammaliaforms^3,6,7,10–17^.

In extant mammals, the certain middle ear characters, such as the malleus–incus configuration, are more conserved in evolution than other aspects of the middle ear. For example, monotreme middle ears are very distinctive from those of therians. The therian middle ears can be recognized in six morphotypes according to Fleischer^2^ (ancestral ear, microtype ear, transitional ear, freely mobile ear, and two cetacean ears, the *Kogia*-type and *Tursiops*-type), and these morphotypes are known to have functional differences^2,18,19^. In contrast, there are still many open questions regarding the evolution of the middle ear in Mesozoic mammaliaforms. Recent fossil discoveries from the Middle-Late Jurassic Yanliao Biota and the Early Cretaceous Jehol Biota continue to shed light on the evolution of the middle ear in stem clades (e.g., eutriconodontans^3,7,8^, multituberculates^20,21^, haramiyidans^9,22,23^, “symmetrodontans”^24,25^). Recently, much work has been devoted to developing models for the evolution of the middle ear in mammaliaforms^8,9,14,17,20,21,25–28^, to elucidate when and how modern mammals inherited the configuration of the ossicular chain from their Mesozoic ancestors. For instance, the overlapping configuration of the incudomallear joint was long regarded as unique in monotremes across mammals but is considered already established in Late Jurassic haramiyidans and Early Cretaceous multituberculates^9,20,23^ (see alternative interpretations^21,29^). The saddle-like configuration of the incudomallear joint is near universal among extant therians, but the evolution of the middle ear in earliest therians is poorly known. Prior to discussing the morphological transformation of the incuodmallear joint, one of the fundamental questions is what a saddle-shaped incudomallear joint looks like in Cretaceous stem taxa. To date, the best evidence for the middle ear in earliest therians is from Early Cretaceous *Ambolestes*^30^, *Cokotherium*^31^, and Late Cretaceous *Uchkudukodon nessovi*^32,33^. It is still unknown how the middle ear detached from the mandible and how the saddle-shaped incudomallear joint evolved in cladotherians. In this paper, we attempt to answer these questions on the innovation of the middle ear in early eutherians based on a newly discovered specimen from the Early Cretaceous Jiufotang Formation of the Jehol Biota. We also discuss how morphological transformations in the auditory apparatus (Meckelian cartilage and middle ear bones) may be related to the development of the masticatory apparatus in the evolution of mammaliaforms.

## Results and discussion

### Systematic paleontology

Class Mammalia Linnaeus, 1758

Infraclass Eutheria sensu Huxley, 1880

Order incertae sedis

Family incertae sedis

*Microtherulum oneirodes* gen. et sp. nov. Wang et Wang, 2023.

#### Etymology

The generic name *Micro* refers to the microtype configuration of the middle ear; *therulum*, little beast. The specific name from *oneirodes* (Greek), dreamlike, refers to the dreamlike fossil discovery that fills the gap in the middle ear evolution in early therians.

#### Holotype

**Fig. 1:**
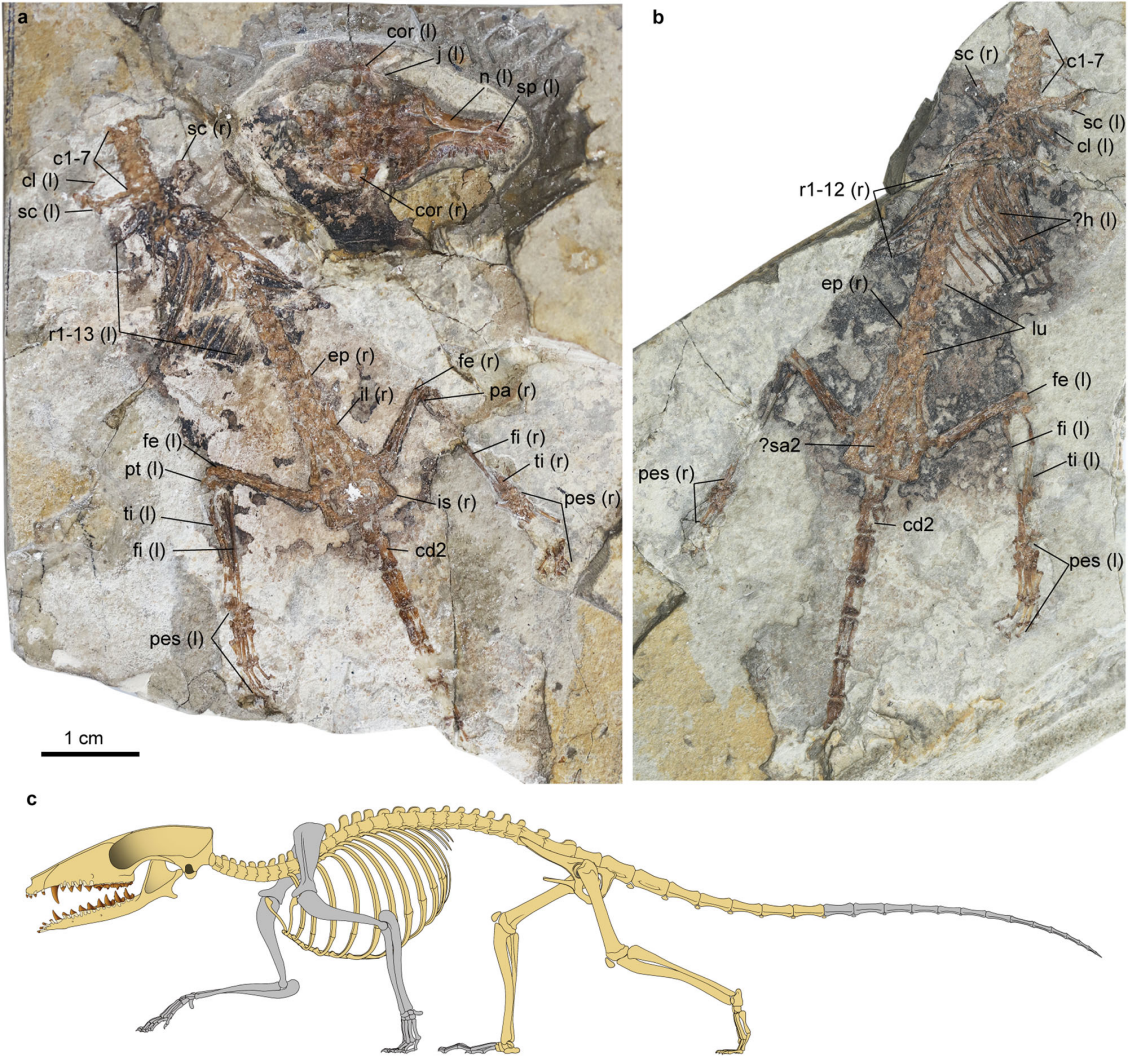
Holotype specimen (IVPP 24190) of the Early Cretaceous eutherian *Microtherulum oneirodes*. **a** Main slab A (IVPP V24190A). **b** Counterpart slab B (IVPP V24190B). **c** Restoration of the skeleton (Gray shading denotes damaged elements). c1-7 cervical vertebrae 1-7, cd2 caudal vertebra 2, cl clavicle, cor coronoid process, ep epipubis, fe femur, fi fibula, h humerus, il ilium, is ischium, j jugal, n nasal, pt patella, r1-13 ribs 1-13, sa sacral vertebra, sc scapular, sp septomaxilla, ti tibia.

**Fig. 2:**
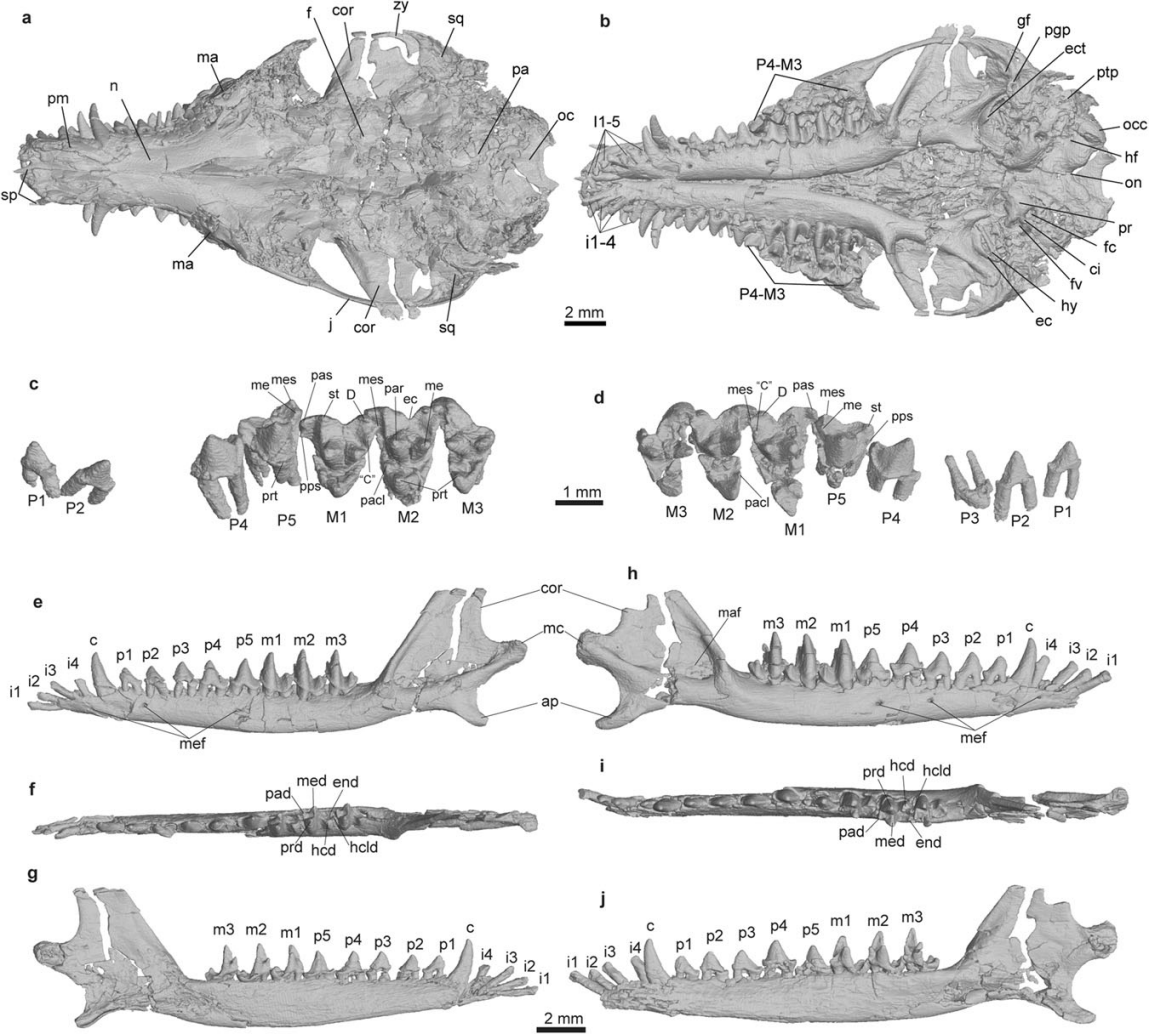
Skull morphology of *Microtherulum*. **a**, **b** Virtual reconstruction of the skull in dorsal (**a**) and ventral (**b**) views. **c** Left upper cheek teeth in occlusal view. **d** Right upper cheek teeth in occlusal view. **e**, **g** Left mandibles in lateral (**e**), occlusal (**f**), and medial (**g**) views. **h**, **j** Right mandible in lateral (**h**), occlusal (**i**), and medial (**j**) views. ap angular process, C stylar cusp C, “C” stylar cusp in postmetacrista, ci crista interfenestralis, cor coronoid process, D stylar cusp D, ec ectoflexus, ect ectotympanic, end entoconid, f frontal, fc fenestra cochleae, fv fenestra vestibuli, gf glenoid fossa, hcd hypoconid, hcld hypoconulid, hf hypoglossal foramen, hy hyoids, I1-5 upper incisors 1-5, i1-4 lower incisors 1-4, j jugal, ma maxilla, maf masseteric fossa, mc mandibular condyle, me metacone, med metaconid, mef mental foramen, mes metastyle, n nasal, oc occipital, occ occipital condyle, on odontoid notch, P4-M3 penultimate and ultimate upper premolars (P4 and P5), and upper molars M1, M2, and M3, pa parietal, par paracone, pacl paraconule, pad paraconid, pas parastyle, pgp postglenoid process, pm premaxilla, pps preparastyle, pr promontorium, prd protoconid, prt protocone, ptp posttympanic process, sp septomaxilla, sq squamosal, st stylocone.

**Fig. 3:**
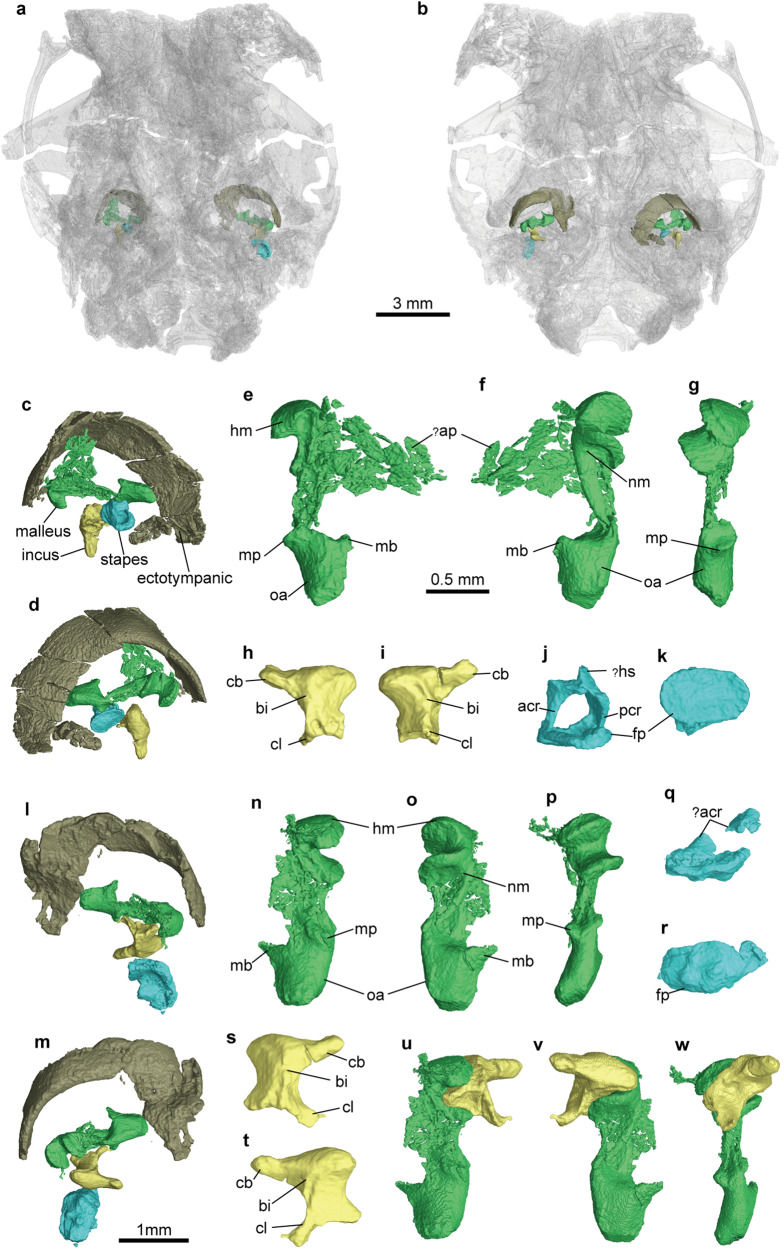
Middle ear bones of *Microtherulum oneirodes* based on virtual reconstruction with malleus in green, incus in yellow, stapes in blue, and ectotympanic in brown. **a**, **b** Virtual reconstruction of the posterior part of skull in dorsal (**a**) and ventral (**b**) views with transparency. **c**, **d** Left middle ear bones in dorsal (**c**) and ventral (**d**) views of the specimen. **e**, **g** Left malleus in dorsal (**e**), ventral (**f**), and posterior (**g**) views. **h**, **i** Left incus in dorsal (**h**) and ventral (**i**) views. **j, k** Left stapes ventral (**j**) and medial (**k**) views. **l**, **m** Right middle ear bones in dorsal (**l**) and ventral (**m**) views of the specimen. **n**, **p** Right malleus in dorsal (**n**), ventral (**o**), and posterior (**p**) views. **q**, **r** Right stapes in ventral (**q**) and medial (**r**) views. **s**, **t** Right incus in dorsal (**s**) and ventral (**t**) views. **u**, **w** Restoration of right incudomallear articulation in dorsal (**u**), ventral (**v**), and posterior (**w**) views. acr anterior crus of stapes, ap anterior process of malleus, bi body of incus, cb crus breve, cl crus longum, fp footplate of stapes, hm head of malleus, hs head of stapes, in incus, ma malleus, mb manubrial base, mp muscular process, nm neck of malleus, oa orbicular apophysis, pcr posterior crus of stapes, st stapes.

A nearly complete skeleton preserved on the main (IVPP V24190A) and counterpart slabs (IVPP V24190B) at the Institute of Vertebrate Paleontology and Paleoanthropology, Beijing, China (Figs. 1–3; Supplementary Note 1, Supplementary Figs. 1–3, and Supplementary Table 1).

Locality and age: The holotype is from the Jiufotang Formation at Yangjiaogou, Chaoyang City, Liaoning, China, dated to 118–120 million years ago^34–37^.

#### Diagnosis

I5-C1-P5-M3/i4-c1-p5-m3 (I: incisor; C: canine; P, premolar: M, molar; lower cases denote lower teeth) (Fig. 2). Differs from all pre-tribosphenic and pseudotribosphenic clades in distinct tribosphenic molars. Differs from metatherians in having a typical eutherian dental formula and in lacking inflected angular process, keel-like paraconid, and twinning of hypoconulid and entoconid^38^. Among Jurassic-Early Cretaceous eutherians, *Microtherulum* is similar to *Juramaia*^39^ and *Eomaia*^40^ in having the same dental formula; differs from *Acristatherium*^41^, *Ambolestes*^30^, *Cokotherium*^31^, *Sinodelphys*^42^, and *Sasayamamylos*^43^ in having different counts of upper/lower incisors and premolars. Differs from *Prokennalestes* and *Murtoilestes* in lacking metaconule in upper molars. It has no Meckelian sulcus as in *Sasayamamylos* and *Montanalestes*^44^ but differs from *Eomaia*, *Ambolestes*, *Cokotherium*, *Sinodelphys*, and *Prokennaleste*s^45^ that have a Meckelian sulcus. Differs from *Ambolestes*, *Cokotherium*, and Late Cretaceous eutherians in having a protoconal swelling in the penultimate upper premolar. Differs from *Juramaia*^39^ in having three mental foramina, a single-rooted upper canine, smaller paracone in P4 (compared to P5), preparastyle in P5, transversely narrow upper molars with a more expanded and higher protocone, paraconule more labially positioned in upper molars, and in lacking metaconule on upper molars. Differs from *Eomaia*^40^ in having a large trenchant P4, multiple mental foramina, more prominent entoconid on the anteroposteriorly longer talonid in lower molars, and in lacking a diastema between the lower canine and p1. Differs from *Acristatherium*^41^ in having a shallower extoflexus, greater size differential between P4-5, a posteriorly increasing size in p1-5, and a more extended talonid basin in lower molars. Differs from *Sinodelphys*^42^ in lacking a distinct diastema in the upper and lower dentition. Differs from *Ambolestes*^30^ that has a distinct diastema in upper and lower dentition, a Meckelian groove, and a broader talonid in lower molars. Differs from *Cokotherium*^31^ in having a larger metastylar lobe in M1-2, a more distinct paraconule in upper molars, a larger metacone in M3, and a shorter angular process, and in lacking ossified Meckelian cartilage and expansion of ectotympanic. Differs from *Ssayamamylos*^43^ that has smaller anterior lower premolars and broad coronoid process. Differs from *Prokennalestes*^45^ in having a single-rooted lower canine, less-developed metacone and protocone in ultimate upper premolar, in lacking a distal metacristid in lower molars and masseteric foramen. Differs from *Montanalestes*^44^ in having small paraconid (relative to metaconid) in lower molars and non-molarized p5 (see Supplementary Discussion for morphological comparisons among early eutherians).

### Description

The skull is nearly completely preserved and compressed in slab A (IVPP V24190A), exposed in dorsal view (Figs. 1 and 2). The ventral aspect of the cranium is visible in the images from the CT scans. The premaxilla is slender and its facial process extends posteriorly to the level of the upper canines. Dorsally, its facial process contacts the nasal along its entire length. Anteromedial to the premaxilla, a pair of slender projections are present, interpreted as the vestigial septomaxillae, which probably sit on the dorsal surface of the premaxilla in the lateral view of its anatomical position, but they may be slightly displaced (laterally on the left and medially on the right) due to compression of the cranium (Fig. 2). The nasal gradually expands posteriorly and its length is more than half the length of the cranial length. The suture between the frontal and the maxilla is not clear due to distortion and these two bones probably do not have facial contact in dorsal view. The frontal and parietal are compressed with numerous cracks and the suture between the two bones cannot be confidently identified. The postorbital process is vestigial. The zygomatic arch is slender and the zygomatic process of the maxilla is vestigial. The anterior zygomatic root is at the level of the ultimate upper molar. The lateral and ventral aspects of the braincase are badly damaged. The sagittal crest is weak. The morphology of the palate is mostly obscured by the mandibles in ventral view, and the posterior aspect of the palatine is badly damaged. In the basicranial region, the promontorium is bulbous in ventral view. No vascular groove for the internal carotid can be identified on the ventral surface of the promontorium. The fenestra vestibuli is oval on the right side, while the fenestra cochlea is nearly circular in ventral view. The crista interfenestralis between the fenestra cochleae and the fenestra vestibuli is well-defined in ventral view and extends toward the paraoccipital process posteriorly. Anterolateral to the promontorium are hyoid bones, either the epihyal or thyrohyal. The glenoid fossa is concave and oval. The postglenoid process is small on the posterior rim of the glenoid fossa and the postglenoid foramen is absent (Fig. 2a, b). Anterior to the occipital condyle is a single hypoglossal foramen on the left side.

The mandibles are nearly complete with damage to the coronoid process and near the root of the angular process (Fig. 2e, h). Three mental foramina are present on the lateral side of the mandible. The symphysis of the mandible extends posteriorly to the level of the lower canine. The mandible is slender and the ventral margin anterior to the angular process is concave. The angular process is short, low, and posteroventrally directed, and its root is located anteroposteriorly at the level of the coronoid process. The mandibular condyle has an oval outline with a distinct neck. The coronoid process is broad and high, with its dorsal part being slightly damaged. The masseteric fossa is broad and deep in lateral view and the masseteric foramen is absent. The Meckelian sulcus is probably absent because it has no sign of anterior extension for a sulcus on the medial aspect of the posterior portion of the mandible compared with that in *Cokotherium* and *Ambolestes* (Fig. 2g, j), although the possibility of the presence of a quite vestigial Meckelian sulcus cannot be completely ruled out.

There are five small upper incisors in the premaxilla. The upper incisors are conical, sub-equal in size, and separated by small diastemata (Fig. 2c, d). The upper canine is single-rooted and larger than the upper incisors. The postcanine loci consist of five for the upper premolar series (P1–P5) and three for the upper molars (M1–M3), with the middle upper premolar (P3) not preserved on the left side. The anterior upper premolars (P1–P4) are double-rooted, while the ultimate upper premolar (P5) bears three roots. The anterior upper premolars (P1–P3) are similar in having a trenchant tooth crown. The P3 is the smallest in the postcanine row. The penultimate upper premolar (P4) is pointed, and its tooth crown is not distinctly higher than that of P5. The tooth crown of P5 is transversely expanded and has a protocone on the lingual side. The P5 is narrower in width than that of the M1. The P5 develops a stylar shelf, on which the metastylar lobe is slightly larger than the parastylar lobe. On the lingual side of P5 is a small protocone that is much lower than the paracone of P5. There is a tiny cusp (preparastyle) anterolingual to the stylocone on P5. Three upper molars (M1–M3) are typically tribosphenic and transversely wide. The M2 is transversely wider than M1 and M3 based on the well-preserved left upper molars, while the right upper molars have distinct cracks between the paracone/metacone and protocone, making them superficially wider than the left molars. The tooth crown of M1-2 has a triangular outline in occlusal view. The stylar shelf is relatively narrow transversely with its width being less than half the total width of the upper molars. The preparastyle is distinct and forms a groove for the protoconid with a much larger parastyle and stylocone in the M1-2. The preparacrista is well-developed and the paracone is larger and higher than the metacone in the upper molars. The paraconule is small and situated near the middle of the preprotocrista, while the metaconule is absent in the upper molars. The protocone is low, without anteroposterior extension, and the hypocone is absent in the upper molars. The cusp “C” is prominent in the postmetacrista. The style C cusp is absent in the stylar shelf of the upper molars. The metastylar lobe is reduced in the M3.

Four lower incisors are procumbent and sub-equal in size (Fig. 2e, h). The lower canine is single-rooted and larger than the lower incisors. There are eight lower postcanine loci in the mandible, five lower premolars (p1-5) and three lower molars (m1-3). The lower premolars are double-rooted and increase in size posteriorly. All lower premolars have similar morphology without the development of the talonid. The heel becomes more prominent in the posterior lower premolars (p3-5). Three lower molars are similar in size. The protoconid is distinctly significantly higher than the paraconid and metaconid in the lower molars. The metaconid is slightly lower in height than the paraconid and more robust than the paraconid. The anterolabial cingulid is prominent in the lower molars. Three lower molars have a well-developed talonid. The anteroposterior length of the talonid is approximately equal to that of the trigonid and is almost as wide medio-laterally. The hypoconulid is higher than the entoconid and the hypoconid, located transversely between the latter two cusps on the talonid. The entoconid is more prominent on the m1 than on the m2-3.

### Middle ear morphology

**Fig. 4:**
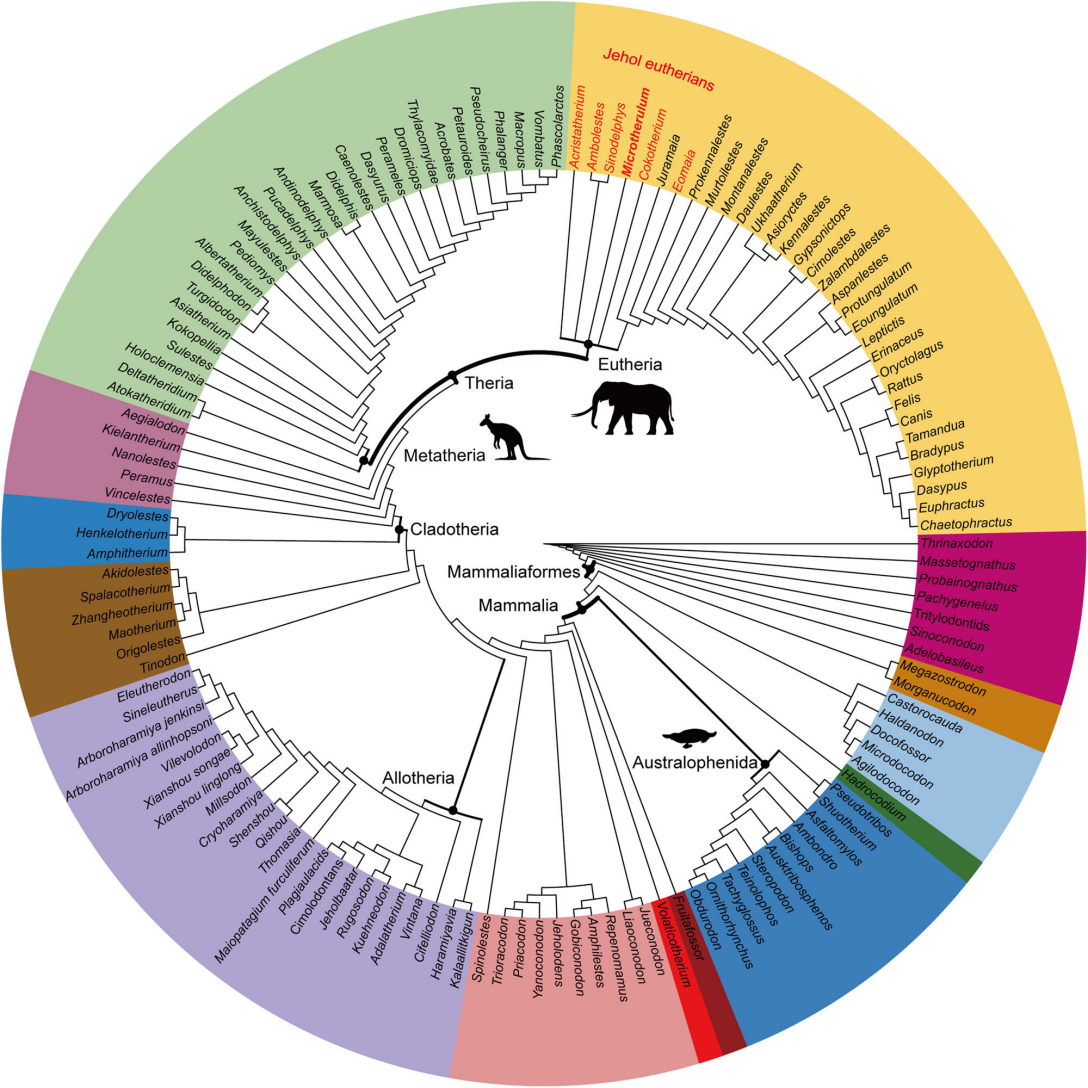
Phylogeny of Mesozoic mammaliaforms. The topology is based on the strict consensus of parsimony analysis using a modified morphological character matrix from previous studies^9,20,72,73^. Eutherians discovered in Early Cretaceous Jehol Biota are on the upper part of the tree with their names highlighted in red.

**Fig. 5:**
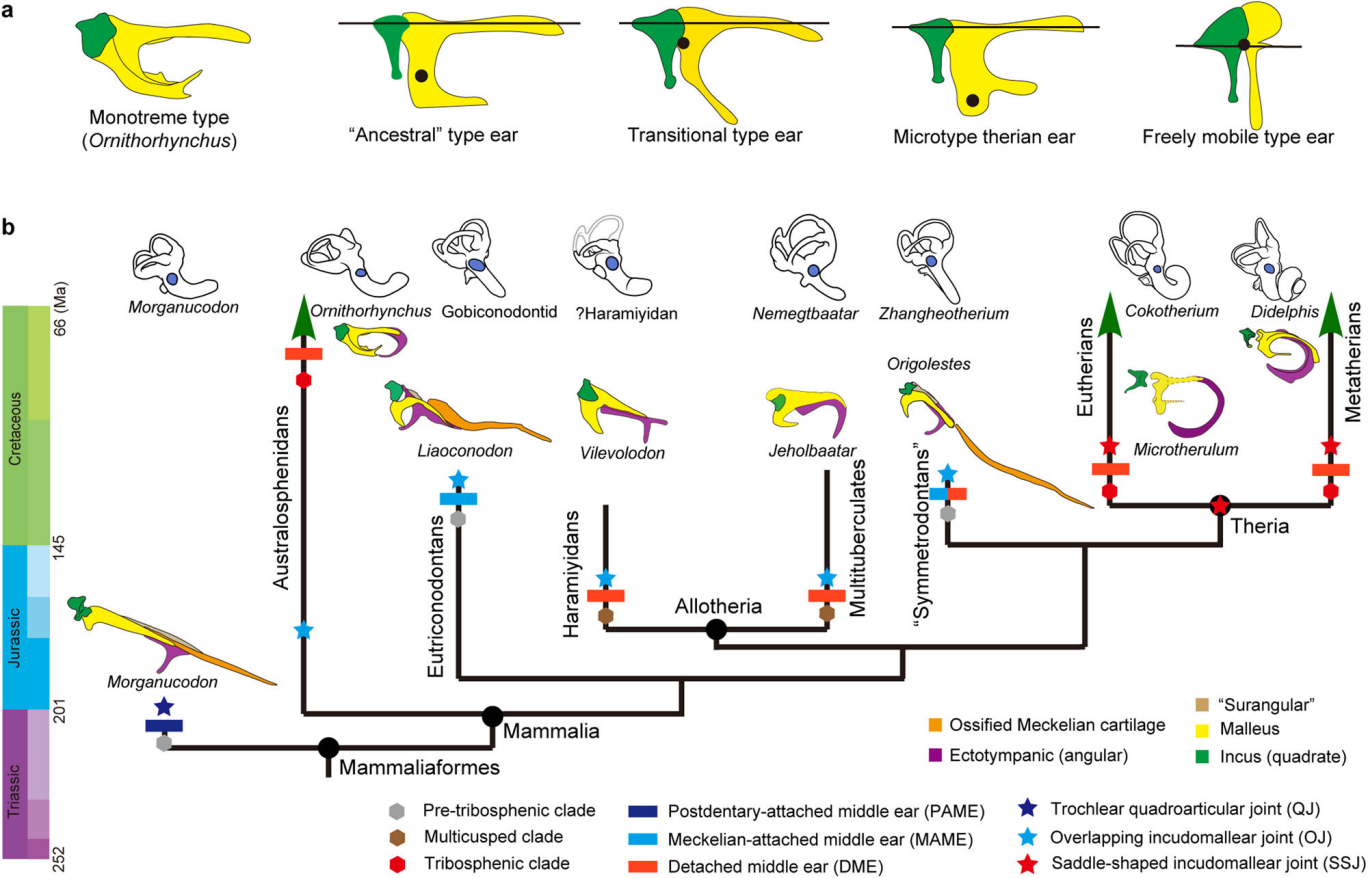
Ear evolution in Mesozoic mammaliaforms. **a** Morphotypes of the incudomallear joint in dorsal view in extant monotremes and therians^2, 18^, ancestral type (opossums), transitional type (hamsters), microtype ear (mice and bats), and freely mobile type (squirrels). In each morphotype, the horizontal line denotes the anatomical axes, and the dot denotes the estimated mass center of the ossicular chain. **b** Evolutionary events associated with hearing and chewing apparatuses are mapped in the simplified phylogeny, including development of tribosphenic molars, detachment of middle ear from mandible, transformation of incudomallear joints, and coiling of cochlear canal. The simplified topology is modified from the strict consensus of the parsimony analysis (Fig. 4), using a morphological character matrix modified from several sources^9,20,72,73^. The pattern of ear evolution highlights three morphotypes of the middle ear (PAME, MAME, and DME) and three morphotypes of the incudomallear joint (QJ, OJ, and SSJ), coupled with the innovation of tribosphenic teeth in non-allotherian clades. Schematic illustrations of the middle and inner ear are modified from multiple sources^6,20,25,55,71^. Different elements of the middle ear are marked, ectotympanic in purple, malleus in yellow, incus in green, and ossified Meckelian cartilage in orange.

*Microtherulum* is the first Mesozoic eutherian with a middle ear preserved nearly entirely on both sides (Figs. 2–5). The ectotympanic is a C-shaped bone (Fig. 3c, d, l, m). The posterior crus of the ectotympanic, damaged at the distal end, is slightly expanded compared to the anterior crus. No distinct sulcus is observed around the contact between the anterior crus of the ectotympanic and the anterior process of the malleus. The ectotympanic is probably positioned obliquely rather than horizontally, a feature typical of most eutherians but distinct from monotremes^46,47^. The ectotympanic differs from the elongate, likely distorted “U-shaped” ectotympanic with less expansion as observed in Late Cretaceous *Uchkudukodon*^32^. It also differs from the fusiform ectotympanic with expansion in the anterior crus represented in Late Cretaceous Djadokhta eutherians (e.g., *Asioryctes* and *Zalambdalestes*)^46,48^. The ectotympanic ring is relatively more expanded in shape in *Microtherulum* than that of Early Cretaceous *Ambolestes*.

The malleus is a massive bone in the middle ear (Fig. 3e–g). This bone is fairly well-preserved in the specimen, and its reconstruction is combined by multiple featured pieces, including the malleus head and incudomallear joint facet, the orbicular apophysis, and the manubrial base. The head of the malleus is round and blunt with a distinct neck. Anterior to the head of the malleus are bony fragments, probably representing the compressed anterior process of the malleus on the left side, but this part is badly damaged on the right side. The body of the malleus bears an incomplete muscular process on the dorsal side. Anteriorly, the manubrial base tapers and the manubrium of the malleus is badly damaged (likely elongated). In addition to the muscular process, the malleus is featured by a well-defined projection with a constricted base near the manubrial base, interpreted here as the orbicular apophysis. Given its size relative to the head of the malleus, the orbicular apophysis in *Microtherulum* is more prominent than those of hedgehogs and opossums^49,50^. The malleus in *Microtherulum* is unique in that it has a distinct orbicular apophysis compared to all known Mesozoic mammaliaforms, which by definition is indicative of the microtype middle ear^2,18,51^. There may be a slight distortion between the neck of the malleus and the orbicular apophysis, with the left malleus being more prominent. Unlike other Mesozoic mammaliaforms, the massive malleus with the orbicular apophysis in *Microtherulum* represents a morphological innovation in the middle ear. The relatively well-preserved saddle-shaped joint of the malleus and the incus (Fig. 3u–w) is similar to that in extant therians, and reveals new information rarely preserved in Mesozoic mammaliaform fossils. The incus has a body and two processes (Fig. 3h, i, s, t), similar to that of many living mammals (e.g., carnivorans, rodents, and bats)^2,5^. The crus breve (short process) of the incus is rod-shaped. The crus longum (long process) of the incus is probabaly damaged at the distal end, with the right side being more complete. In the right incus, the preserved part of the crus longum is shorter than the crus breve due to the brekage and tapers distally. The lenticular process is not preserved in *Microtherulum*. The stapes is slightly damaged and the most prominent feature of the stapes is the oval footplate (Fig. 3j, k, g, q, r). The stapedial foramen is large, bordered by the anterior crus and the posterior crus of the stapes. The head of the stapes is incomplete. The robust interlocking between the malleus and incus in a saddle-shaped joint in *Microtherulum* exclusively resembles that of therian middle ears and contrasts with the overlapping or partial overlapping condition seen in haramiyidans, multituberculates, eutriconodontans, zhangheotheriids, and monotremes^8,20,23,25^.

### Fate of the Meckelian cartilage (MC)

The absence of the Meckelian sulcus suggests that there was no substantial bony or cartilaginous contact between the mandible and the middle ear in *Microtherulum* (Figs. 2 and 3). This is consistent with a growing body of evidence from other mammaliaforms that the presence of the Meckelian sulcus is the best available osteological correlate for inferred connection of the middle ear to the mandible, and absence of the sulcus represents the loss of the Meckelian element^3,7,9,52^. The MC can be ossified as an essential link between the mammalian middle ear and the mandible in several basal mammaliaforms, including docodontans, eutriconodontans, and “symmetrodontans”^3,8,25,26,53^. Evidence of the MC is sparse and indirect in the fossil record of early eutherians, and a vestigial Meckelian sulcus is retained in *Ambolestes* and *Prokennalestes*^30,45^. Some Early Cretaceous eutherians have the DME (as observed in *Microtherulum*), while others (as observed in *Cokotherium*) still retain a gracile ossified MC, a precursor condition to DME^31^ (see Supplementary Fig. 1 and Supplementary Discussion). It appears that degradation of the Meckelian cartilage is also variable among Early Cretaceous eutherians, suggesting a high evolutionary variability in the detachment of the middle ear in early mammalianforms except for allotherians. The loss of the MC evolved earlier in allotherians in the Late Jurassic than in other mammals in the Early Cretaceous or later^20,23,54,55^. Developmental studies suggest that the MC retains the capacity of ossification and can persist with a corresponding Meckelian groove beyond juvenile stages by knockout of chondroclasts in development in mutant mice^14^. Fossil evidence would provide valuable sources for investigating the fate of different parts of the MC in temporal and spatial frameworks in developmental studies^14,16,17,56^.

### Evolutionary innovation of incudomallear joint in deep time

In living mammals, the configuration of the incudomallear joint is conservative, and shows a clear distinction between monotremes and therians. The incudomallear joint has an overlapping configuration in extant monotremes and is saddle-shaped in therians^6,7,20,27^. The incudomallear joint configuration is quite different between extant monotremes (overlapping joint) and therians (saddle-shaped joint). The malleus–incus morphology of several Mesozoic mammaliaforms has been debated, with alternative interpretations for the evolution of incudomallear structures^9,57,58^. The malleus and incus of *Microtherulum* are the earliest-known evidence of the saddle-like incudomallear joint. It suggests that this novel configuration originated no later than in eutherians of the Early Cretaceous (Figs. 3 and 5). The broad and saddle-shaped incudomallear facets as shown in *Microtherulum* are in contrast to a relatively thin, flat (or slightly concave) facet as shown in Late Jurassic haramiyidans (*Arboroharamiya* and *Vilevolodon*)^9,23^ (see alternative interpretation^22^) and Early Cretaceous mammals (*Liaoconodon*, *Jeholbaatar*, *Origoleste*s, and *Sinobaatar*)^20,21,25^ (see alternative interpretations^21^). The new fossil of *Microtherulum* may shed light on the debate about alternative interpretations on the evolution of the incudomallear joint morphotypes. Compared to *Microtherulum* and extant therians, the so-called transitional form (partial overlapping joint^9^ or braced hinge joint^21^) as recently described for Late Jurassic and Early Cretaceous mammaliaforms seems redundant due to its minor distinction from the overlapping joint^58^. In addition, during cranial development, the incus has two contacts, the incudomallear and incudopetrosal contacts. The incudopetrosal contact is closely associated with incus morphologies during development in all three groups of living mammals^27^. This suggests that different strategies for stabilizing the jaw hinge, that may not involve additional transitional stages (e.g., “braced incudomallear joint”)^21^. The incudomallear joint in *Microtherulum* leads to a new hypothesis that the derived saddle-shaped incudomallear joint of extant therians evolved no later than the Early Cretaceous.

### Phylogeny

Phylogenetic analysis using parsimony reveals that *Microtherulum* is clustered with other Early Cretaceous Jehol therians (*Cokotherium*, *Acristatherium*, *Ambolestes*, and *Sinodelphys*), which are placed as early branching members of the monophyletic Eutheria in the strict consensus (Fig. 4). The topology among early branching therians is different in the 50% majority consensus of the parsimony analysis (see Supplementary Figs. 4 and 5). It is debatable about the accuracy and precision of the phylogenentic reconstruction over different phylogenetic methods, and here we follow the strict consensus of the parsimony analysis. Current phylogeny suggests that *Juramaia* is not the most basal lineage of eutherians and is comparable to Jehol eutherians, despite the debate on its stratigraphic occurrence by a temporal gap of about 40 million years, either Late Jurassic (about 160 million years ago)^39,59^ or Early Cretaceous (about 120 million years ago)^60,61^.

### Decoupling of hearing and chewing apparatuses

DME evolved multiple times in Mesozoic mammaliaforms based on the current phylogeny (also see Supplementary Discussion) (Fig. 5b). The occurrence of the DME is one of the most important innovations in the evolution of the mammalian dentary and middle ear^6,7,62^. Postdentary bones in nonmammalian cynodonts were miniaturized and transformed towards the complete detachment of the middle ear by the gradual MC degradation in mammaliaforms, ultimately leading to the decoupling of hearing and chewing apparatuses^6–8,63^. The evolution of decoupling of the ear from the jaw has long been postulated, and gained for increasingly stronger support from new relavant materials preserved in mammaliaform fossils of the Jurassic and Early Cretaceous^9,20,25,31^. The decoupling event of hearing and chewing apparatuses is well established based on the evidence of different evolutionary stages in early eutherians, evidenced by the discovery of the ossified MC and inner ear in *Cokotherium*^31^, the Meckelian sulcus for MC and middle ear in *Ambolestes*^30^, and the detached middle ear in *Microtherulum* (Fig. 5b). The evolution of chewing apparatuses involves several aspects, such as the evolution of tooth structure (increase in shearing and grinding function from triconodont to tribosphenic tooth, or increase in morphological complexity of multicusped tooth in allotherians) and the evolution of molar occlusion (e.g., anteriorly directed occlusion in cladotherians and posteriorly directed occlusion in allotherians)^62,64,65^. Adaptation for efficient chewing has been proposed as the underlying mechanism for the decoupling event^20,25^, and allotherians represent the earliest clade to evolve DME in the Middle-Late Jurassic^9,20,23^. In non-allotherian mammaliaforms, this event was recently proposed in Early Cretaceous zhangheotheriid *Origolestes*^25^, as evidenced by a gap between the middle ear and the ossified MC. There are alternative interpretations^9,55^ that suggest the gap between the middle ear and ossified MC may be a fossil fracture rather than an anatomical structure^55^. Given that the Meckelian groove is absent in some spalacotheriids^66,67^, the decoupling in “symmetrodontan” probably evolved independently from that in cladotherians under current phylogenetic framework^24^. Compared to mammaliaforms with the PAME or MAME^8,26,68^, the free vibration of the middle ear system in early eutherians can be profoundly improved by DME acquisition (in *Microtherulum*, *Montanalestes*, and *Sasayamamylos*)^43,44^ or a DME precursor with a gracile Meckelian cartilage (in *Cokotherium* and *Ambolestes*)^30^, and a saddle-shaped incudomallear joint (in *Microtherulum*), together with the innovation of inner ear structures (e.g., the presence of a bony cribriform plate, a primary bony lamina and the base of the secondary bony lamina, as well as a nearly 360° coiled cochlear canal)^31,69^. Early Cretaceous eutherians had probably achieved better hearing by expanding the range of audible frequencies than Jurassic and other Early Cretaceous mammaliaforms, such as docodontans^70^, haramiyidans^71^, eutriconodontans^4^, zhangheotheriids^4,25^, and multituberculates^4^. The evolution of typical tribosphenic molars in therians, especially eutherians, may have enhanced efficient shearing and grinding functions during the Cretaceous evolution, which was also facilitated by degradation of the Meckelian cartilage that further reduces or removes the interference of mastication^31^, and by the development of a posteriorly directed angular process and modifications of anteriorly directed muscle force vectors during occlusion^65^. The early onset of adaptation to hearing and feeding in Early Cretaceous eutherians may have profound implications for the evolutionary success of therians later in Late Cretaceous and Cenozoic.

## Methods

The holotype specimen of *Microtherulum* (IVPP V24190) is a partial skeleton of an Early Cretaceous eutherian mammal, preserved in the main slab (IVPP V24190A) and the counterpart slab (IVPP V24190B). It was discovered in the Jiufotang Formation at Yangjiaogou site in Chaogyang city, Liaoning Province, China. It is currently housed in the collection of the Institute of Vertebrate Paleontology and Paleoanthropology (IVPP), Chinese Academy of Sciences, Beijing, China. The main slab of the holotype specimen was scanned using high-resolution micro-computed tomography at the Key Laboratory of Vertebrate Paleontology and Paleoanthropology at the Institute of Vertebrate Paleontology and Paleoanthropology and the Yinghua Inspection and Testing using a GE V|Tome|xm dual tube. The skull was scanned at a resolution of 10.567 μm per pixel (210 kV, 130 μA) and the posterior part of the skull was scanned at a resolution of 5.507 μm per pixel (100 kV, 150  μA). Segmentation was conducted in the VGStudio 3.0. In this study, the illustrations for the segmented middle ear bones based on high-resolution CT scan data might be slightly different from anatomical orientations of these bones due to slight distortion and compression of these fragile structures during the fossil preservation.

The character list was modified from previous studies^9,20,23^ with additional 61 characters adopted from two recent studies^72,73^ (see Supplementary Note 2). Characters are modified to ensure that each phylogenetic character is an organismal feature expressed as an independent variable^74^. Newly published mammaliaforms represented by well-preserved specimens were added in taxon sampling (e.g., *Spinolestes*, *Microdocodon*, *Kalaallitkigun*, *Jueconodon*, and *Cokotherium*)^72,73,75,76^, while *Sinobaatar* was excluded. The new character matrix for phylogenetic analysis is composed of 615 characters and 135 taxa. Data matrices were edited in Mesquite V. 3.7. Parsimony analysis was performed on Mac (supercharged by M1 Max) using TNT 1.6 with New Technology Search method^77^, implementing sectorial search, ratchet (200 iterations), drift (100 cycles), and tree fusing (10 rounds), under equally weighted parsimony^78–80^. Parsimony analysis returned 13 most parsimonious trees, with a length of 3125, CI = 0.306, RI = 0.792. The length of the strict consensus is 3125, CI = 0.298, RI = 0.784.

### Reporting summary

Further information on research design is available in the Nature Portfolio Reporting Summary linked to this article.

### Supplementary information

Supplementary Information

Reporting Summary

## Data Availability

The holotype of *Microtherulum oneirodes* (IVPP V24190) is housed in the collection of the Institute of Vertebrate Paleontology and Paleoanthropology (IVPP), Beijing, China. All data supporting the findings of this work (specimen, ct scan, virtual reconstructions) are available at IVPP. The data matrix for the phylogenetic analysis is available in the Supplementary Note 2. The original CT data can be shared on request via the Collection Department at IVPP. This published work and the nomenclatural acts it contains have been registered in ZooBank, and the Life Science Identifiers (LSID) for the new genus and species are registered with Zoobank (http://zoobank.org) with the identifiers urn:lsid:zoobank.org:act:5E0910F8-6F4B-46BF-B79C-4E0DF26EB630; urn:lsid:zoobank.org:act:EEC07F0A-F8E5-4D16-9E3C-12A93E27E496.
