# Supplementary material for: Middle ear innovation in Early Cretaceous eutherian mammals
Source: Nat Commun. 2023 Oct 26;14:6831. doi: 10.1038/s41467-023-42606-7 (PMC10603157; doi:10.1038/s41467-023-42606-7)
Supplement: Supplementary file 1 — Supplementary Information [file 41467_2023_42606_MOESM1_ESM.pdf]

Middle ear innovation in Early Cretaceous eutherian mammals

Haibing Wang[1*], Yuanqing Wang[1,2*]

[1] Key Laboratory of Vertebrate Evolution and Human Origins of Chinese Academy of Sciences, Institute of Vertebrate Paleontology and Paleoanthropology, Chinese Academy of Sciences, Beijing, 100044, China

[2] College of Earth and Planetary Sciences, University of Chinese Academy of Sciences, Beijing 100049, China

Corresponding authors: Haibing Wang: wanghaibing@ivpp.ac.cn (https://orcid.org/0000-0001-6811-7262) and Yuanqing Wang: wangyuanqing@ivpp.ac.cn (http://orcid.org/0000-0002-7467-6586)

Supplementary Notes 1

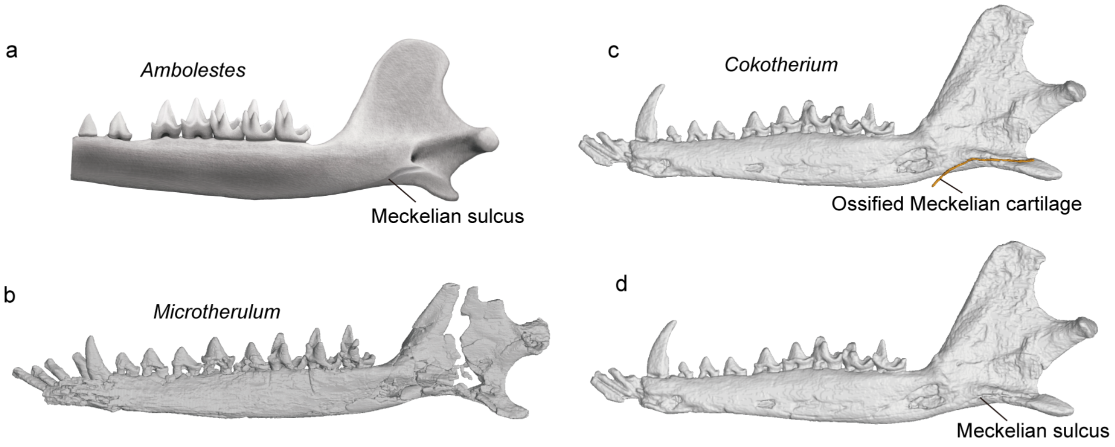

Supplementary Figure 1. Right mandibles in medial view in *Ambolestes*, *Microtherulum*, and *Cokotherium*. **a**, The Meckelian sulcus on the medial side of the right mandible in *Ambolestes* (Bi et al., 2018). **b**, The right mandible of *Microtherulum* in medial view. **c**, The ossified Meckelian cartilage in *Cokotherium* (Wang et al., 2022). **d**, The Meckelian sulcus in *Cokotherium* (Wang et al., 2022).

Descriptions of postcranial skeleton

The axial skeleton is splited in the counterpart slabs, resulting in a lack of sufficient detailed morphological information for most axial bones, except for ribs (Fig. 1, Supplementary Figure 2, Supplementary Table 1). The cervical vertebrae are probably complete from C1 (atlas) to C7 without any evidence of cerveical ribs. In V24190B, a total of 13 pairs of ribs are identified with confidence. Posteriot to the last thorasic vertebra, there are at least 6 lumbar vertebrae identified in V24190B. At least two sacral vertebrae are present. The sacral transverse process of the posterior-most sacral vertebra is distinct and broad. There are seven anterior caudal vertebrae preseved in V24190.

Both scapulars are preseved but not fully exposed in V24190A and V24190B. The outline of the ventral part is exposed in the left scapular, while the dorsal part is exposed in the right scapular. The left clavicle is identified in V24190B. The clavical is not robust, but gentley curved with a blunt acrominal end. The forelimbs are poorly

preseved, and a long bone embed in the left ribs is probabaly the left humerus in V24190B. The left humerus in V24190A is poorly preseved. Its proximal end reflects general outlines of the humerus with a distinct head. The preseved outline of the shaft is relatively straight. At the distal end of the humerus, the outlines of the entepicondyle and ectopicondyle can be identified. The entepicondyle is more prominent than the ectopicondyle.

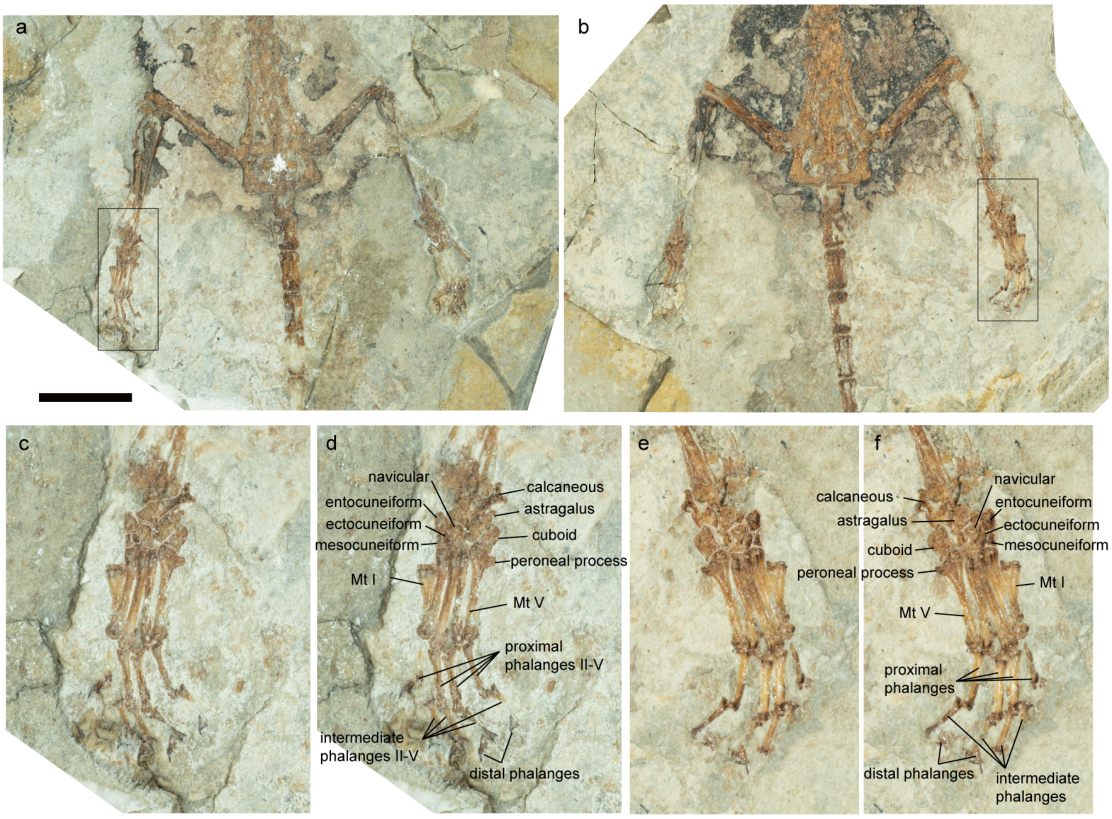

Supplementary Figure 2. The left pes on the main slab (V24190-A) (**a**) and counterpart slab (V24190-B) (**b**) in *Microtherulum*. Close-up views of the left pes on the main slab in dorsal view (**c**, **d**) and on the counterpart slab in ventral view (**e**, **f**). Scale bar, 1 cm.

The ilium and ischium are fused to enclose the femoral head. The sacral transverse process is short and the pelvis is narrow at the sacral joint. The obturator foramen is large and has a oval outline in dorsal view. A small, rod-like bone is preserved anterior to the ischium on the right side, and it probabaly represents the right epipubic bone. The femoral head displays a large, bulbous shape with a distinct neck. The great trochanter is smaller than the femoral head but is subequal to the head in height based

on preserved outlines in V24190A. The femur is badly damaged in the shaft and distal part of the femur. The ossified patella is present in the right hindlimb with a well-defined outline. The femoral shaft has a straight outline. The dital end of the femur is badly damaged. The tibia bends anteriorly with a small degree. The tibial shaft is narrowest at the mid-length and expands proximally and distally, while much of the proximal articular portion is damaged on both tibiae. The medial malleolus is small in distal end of the tibia (V24190A). The fibula is a straight bone and as long as the tibia. The proximal end of the fibula expands distinctly than the shaft and the disatal part. The lateral malleolus is not enlarged in the distal end of the fibula. Distally, the fibula contacts with the tibia in the fibular notch in posterior view (on the right hindlimb in V24190A). The parafibula is absent.

Both peses are preseved with surface fractured and split on the counterpart slabs (Supplementary Figure 2, Supplementary Table 1). The ankle bones of the left pes are more complete. The proximal row of tarsal bones are incomplete and slightly displaced from their anatomical positions. The astragalus is poorly preseved in V24190. The right astragalus has an irregular outline, with details unkown regarding its articular facets with surrounding bones. The calcaneous is the longest element of the tarsal bones. It is featured by a elongate tuber, with a rugose and bulbous dital end. The preserved outline of the navicular is nearly rectangular in the right tarsus. The navicular contacts the entocuneiform, mesocuneiform, and ectocuneiform distally. The distal row of tarsal bones are well defined and more completely preserved. The entocuneiform displays a rectangular outline and larger than the mesocuneiform and ectocuneiform. The entocuneiform contacts the Metatarsal I (Mt I) distally and this joint is offset from the metatarsal II-mesocuneiform joint. The mesocuneiform is the smallest element, while the ectocuneiform is slightly higher than the mesocuneiform. These two bones are narrow lateromedially. The mesocuneiform and ectocuneiform contact metatarsal II and III distally, respectively. The cuboid is elongated and enlarged to be the highest bone in the distal row of the tarsus. The enlargement of the cuboid is aligned with the metatarsal V, and the cuboid correspons to both metatarsal IV and V, given that the distal articular facet of the ectocuneiform with Metatarsal III is too narrow to contact metatarsal IV. The

metatarsal bones are nearly completely preserved on the right side. The lengths of the metatarsals are in the order, Mt I < Mt II < Mt V < Mt IV < Mt III.

The Mt 1 has an expansion and asymmetric proximal articular facet in the proximal part. The medial aspect of the proximal surface has a distinct process. Mt II-IV are more slender than the Mt I. The proximal end of Mt V is more expanded mediolaterally, featured by a prominent peroneal process (proximolateral tubercle).

Supplementary Table 1. Measurements of hindlimbs in *Microtherulum* (IVPP V24190A). (mm)

Length of element	Left	Right
Mt (metatarsal) I	*2.30	
Mt II	3.9	*3.36
Mt III	4.32	*1.73
Mt VI	4.16	*1.62
Mt V	3.96	*1.79
PP (proximal phalanx) I		
PP II		
PP III	2.54	
PP VI	2.41	
PP V	2.27	
IP (intermediate phalanx) I		
IP II	*1.69	1.68
IP III	*1.74	1.83
IP IV	1.82	1.97
IP V	*0.87	1.82
DP (distal phalanx) I	0.96	
DP II	*0.69	1.12
DP III		1.07
DP IV	1.22	1.06
DP V	*0.78	1.08
Femur	13.02	*11.31
Midshaft width of femur	1.46	
Tibia	14.45	*15.01
Midshaft width of tibia	0.78	
Fibula	14.3	*14.48
Midshaft width of fibula	0.49	*0.58

Note: Measurements for incomplete elements are denoted with *.

Supplementary Discussion

***Juramaia* -** The holotype of *Juramaia* is from the Late Jurassic Tiaojishan Formation at the Daxishan site in the Yanliao Biota (Luo et al., 2011). It is the only known Jurassic (earliest) eutherian so far, but recent quantitative analysis suggests an Early Cretaceous age of Juramaia (King and Beck, 2020). *Microtherulum* has three mental foramina on the lateral side of the mandible, while two are present in *Juramaia*. The dental formula is the same in *Juramaia* and *Microtherulum*. In *Microtherulum*, the protocone in the upper molars (particularly in M2) is more expanded and higher than that of *Juramaia*. *Microtherulum* also differs from *Juramaia* in having a single-rooted upper canine, smaller paracone in P4 (compared to P5), preparastyle in P5, transversely narrow upper molars, paraconule more labially positioned in upper molars, three mental foramina, and in lacking metaconule on upper molars. A tiny cusp is present anterolingual to the stylecone, a rare morphology for Early Cretaceous eutherians. The ectoflexus in P5-M2 is considered as deeper than other known Cretaceous eutherians in Juramaia (Luo et al., 2011). In *Microtherulum*, the ectoflexus in upper molars is slightly shallower than that in Juramaia. The angular process is more slender compared to the reconstruction in *Juramaia*. *Microtherulum* has three mental foramina and their location is placed slightly posteriorly than in *Juramaia*. Detailed morphology of the lower molars in *Juramaia* is not available so we are unable to compare the morphological difference of the lower molars (such as height differential between paraconid and metaconid, width differential between trigonid and talonid) between *Microtherulum* and *Juramaia*. Morphological comparisons between *Microtherulum* and *Juramaia* is restricted based on available evidence, because the holotype of *Juramaia* includes the skull and anterior half of the postcranial skeleton, whereas the forelimbs and shoulder girdle are poorly preserved in *Microtherulum*. In addition, the skull of *Juramaia* is exposed mainly in ventral view with the mandibles overlying the crania, and cranial morphologies of the dorsal roof are not exposed.

Eomaia- The holotype of *Eomaia* is from the Early Cretaceous Yixian Formation at the Dawangzhangzi site (Luo et al., 2002). Microtherulum has the same dental formula as *Eomaia*. *Microtherulum* differs from *Eomaia* in morphologies of upper premolars. The upper premolars in *Eomaia* (P1-4) decrease in size posteriorly and lack a large

trenchant P4, unlike those of other Early Cretaceous eutherians (e.g., *Juramaia, Ambolestes, Cokotherium*, *and Microtherulum*). Micortherulum is distinct from Eomaia in the absence of a diastema among upper premolars. The masseteric foramen is absent in *Microtherulum* and *Eomaia*. The meckelian sulcus is present in *Eomaia* but absent in *Microtherulum*. Microtherulum differs from *Eomaia* in having multiple mental foramina, more prominent entoconid on the anteroposteriorly longer talonid in lower molars, and in lacking a diastema between the lower canine and p1. The size differential between paraconid and metaconid is more distinct in Microtherulum than in Eomaia. These two taxa have similar features in tarsal bones, the mesocuneiform and entocuneiform distinctly smaller than the ectocuneiform, a large peroneal process at the proximal part of the metatarsal V. The cuboid is large and has a broad contact with the metatarsal V in *Microtherulum*, while the cuboid is relatively small and contacts the metatarsal IV and V in *Eomaia*. In Microtherulum, the navicular is displaced from its anatomical articulation and not clearly exposed in the specimen. Based on the exposed outline in the specimen (Extended Figure 1), this bone is likely broad and different from the narrow navicular in *Eomaia*.

Acristatherium- The holotype of *Acristatherium* is from the Early Cretaceous Yixian Formation at the Lujiatun site (Hu et al., 2010). Detailed morphological studies of the skull and dentition are still lacking for this taxon. *Microtherulum* differs from *Acristatherium* in different counts of upper and lower incisors. In *Microtherulum,* the size differential between P4 and P5 is less than that in *Acristatherium*, and the trenchant P4 lacks a protoconal swelling on the lingual side in *Acristatherium*. The P5 in *Microtherulum* is wide transversely with three roots and a small protocone, while the P5 in *Acristatherium* bears two roots and a protoconal swelling on the lingual side. The ectoflexus of the upper molars is deeper than in *Acristatherium*. The size differential between the parastylar lobe and metastylar lobe is less in *Microtherulum* than in *Acristatherium*. The distolabial region of M3 is more reduced in *Acristatherium*. *Microtherulum* is also distinct from Acristatherium in having p1-5 posteriorly increasing in size, a more expanded talonid basin in lower molars, and a weakly developed cusp e in lower molars.

Sinodelphys- The holotype of *Sinodelphys* is from the same locality as *Eomaia*, the Dawangzhangzi site in the Early Cretaceous Yixian Formation (Luo et al., 2003). It was originally considered as the earliest metatherian, characterized primarily by four upper molars, seven lower postcanine loci, a distinct diastema in upper and lower premolars, and other features from the carpal bones (Luo et al., 2003). The Meckelian sulcus is absent in *Sinodelphys* and *Microtherulum*. Recently, the dental formula of *Sinodelphys* has been discussed and revised (Bi et al., 2018) and updated phylogenetic analyses reveal that it is a basal eutherian (Bi et al., 2018; Wang et al., 2022). *Microtherulum* has a different dental formula (5-1-5-3/4-1-5-3) than the revised dental formula in *Sinodelphys* (4-1-5-3/4-1-4-3) (Bi et al., 2018). *Microtherulum* differs from *Sinodelphys* in lacking a large and double-rooted canine, a procumbent P1, and a distinct diastema in upper and lower premolars. The entoconid is thought to be approximate to the hypoconulid in the talonid of m1 in *Sinodelphys*, and this is comparable to that of *Microtherulum*. *Microtherulum*, similar to *Sinodelphys*, has a wide navicular compared to other Cretaceous eutherians.

Ambolestes-The holotype of *Ambolestes* is from the Early Cretaceous Yixian Formation at the Xisanjia site (Bi et al., 2018). *Ambolestes* resembles *Sinodelphys* and differs from most Early Cretaceous eutherians in having a reduced dentition (eight upper postcanine loci and seven lower postcanine loci). *Microtherulum* differs from *Ambolestes* in having transversely wide upper molars, more developed protocone in the upper molars, and in lacking a distinct diastema in upper and lower dentition, a mesostyle in upper molars, a reduced Meckelian sulcus, and a masseteric foramen. The tooth crown of the trenchant P4 is not much higher than the P5 in *Microtherulum*, and the size differential between P4 and P5 is more distinct in *Ambolestes*. The paraconid is slightly higher than the metaconid in *Ambolestes*, while the situation is reversed in *Micortherulum*. The hypoconulid is nearly in the middle line of the talonid in the lower molars in *Microtherulum* but is more lingually positioned based on Figure 2d, 2e in Bi et al. (2018). The ectotympanic in *Microtherulum* is a large bone compared to that of Ambolestes. It lacks a curved articular facet with the malleus in *Microthrulum*, and the anterior process of the malleus is probably relatively straight, in contrast to the condition in *Ambolestes*.

Cokotherium- The holotype of *Cokotherium* is from the Early Cretaceous Jiufotang Formation at the Sihedang site (Wang et al., 2022). It is the first known eutherian from the upper part of the Jehol Biota and Microtherulum is the second one discovered in Jiufotang Formation. *Micorotherulum* has a different and complete anterior dentition (upper and lower incisors) compared to *Cokotherium*. *Micorotherulum* differs from *Cokotherium* in having a smaller canine, protoconal swelling in P4, larger metastylar lobe in M1-2, more distinct paraconules in upper molars, larger metacone in M3, and a shorter angular process, and in lacking a Meckelian sulcus and ossified Meckelian cartilage.

Sasayamamylos- *Sasayamamylos* came from the Early Cretaceous Sasayama Group in Japan (Kusuhashi et al., 2013). *Microtherulum* differs from *Sasayamamylos* that has reduced dentition, a posteriorly inclined lower canine, smaller anterior lower premolars, a distinct angle at the posterior end of the mandibular symphysis, and a broad coronoid process.

Prokennalestes-The diagnostic feature of *Prokennalestes* is recently revised (Lopatin Averianov, 2018). Many derived features show up in the posterior upper premolars of *Prokennalestes* compared to Jehol eutherians, especially in P5. *Microtherulum* differs from *Prokennalestes* in having a single-rooted lower canine, a less-developed protoconal swelling in P4, less-developed metacone and protocone in ultimate upper premolar, weakly developed conules in upper molars, in lacking a cusp C in the upper molars, a distal metacristid in the lower molars and a masseteric foramen. The Meckelian sulcus is present in most specimens of *Prokennalestes* (Averianov and Lopatin, 2018).

Morphotypes of the mammalian middle ear and terminology

Conventionally, three morphotypes of the mammalian middle ear were proposed in synthetic reviews (Allin and Hopson, 1992; Luo et al., 2007; Meng et al., 2011; Luo, 2011), including mandibular mammalian middle ear (MME), transitional mammalian middle ear (or Partial mammalian middle ear) (TMME), and definitive mammalian middle ear (DMME). The terminology is widely used and cited in subsequent studies

(Luo et al., 2007; Luo, 2011; Meng, 2014; Luo and Manley, 2020). The increasing number of discoveries on the middle ear in Mesozoic mammaliaforms leads to ambiguities in the definition of these terms, particularly between TMME and DMME (Mao et al., 2020a; Wang et al., 2022). For instance, how the connection between the ossified Meckelian cartilage (OMC) and middle ear bones is interpreted would affect the identification of its morphotype in *Origolestes*. It is debatable whether the gap between the distal end of OMC and the middle ear bones represents the initial step of detachment (DMME) as suggested by Mao et al. (2020a), or whether it is just a fracture of TMME as in other eutriconodonts (e.g., *Liaoconodon* and *Yanoconodon*), zhangheotheriids, and spalacotheroids (e.g., *Zhangheotherium* and *Maotherium*), given the distinct OMC and the elongate Meckelian groove in the medial side of the dentary in *Origolestes* (Luo and Manley, 2020; Wang et al., 2021). Harper and Rougier (2019) proposed a new term, Detached middle ear (DME), for the condition of the crown mammalian common ancestor:

[*Based on the distribution of characteristics within fully adult extant mammals only, one could reasonably conclude that the condition of the crown mammalian common ancestor consisted of a Detached Middle Ear (DME), with auditory ossicles fully independent from the lower jaw apparatus,…*]

Further, the new terminology was updated for the mammalian middle ear in a recent study (Wang et al., 2021), including three morphotypes, Postdentary-attached middle ear (PAME), Meckelian-attached middle ear (MAME), and Detached middle ear (DME).

[*We find these terms uninformative as they do not describe an actual morphology. As replacements, we suggest postdentary-attached middle ear (PAME) for the former (Fig. 1a) and Meckelian-attached middle ear (MAME) for the latter (Fig. 1b). The former (PAME) more precisely could be postdentary- and Meckelian-attached middle ear, but that is too cumbersome. Regarding the latter (MAME), we suggest a broader definition: we do not limit attachment to an ossified Meckel's cartilage, but include taxa with a Meckelian sulcus where an ossified Meckel's cartilage is not known. This assumes that Meckel's cartilage occupies the Meckelian sulcus (Bensley, 1902; Simpson, 1928) and is continuous with the malleus (articular) and gonial (prearticular).*] (Wang et al., 2021)

These terms are descriptive because they incorporate distinct anatomical features of each morphotype (e.g., postdentary trough and Meckelian sulcus). Remarkably, the boundary between MAME and DME is clearly defined. The differentiation between the latter two stages lies in the morphology of OMC and the Meckelian sulcus. The discovery of a gracile OMC in Early Cretaceous Jehol eutherian *Cokotherium* bridges the gap between MAME and DME. The gracile OMC in *Cokotherium* probably loses substantial connection to the auditory ossicles, althought fossil evidence can not guarantee a complete detachment in the case of *Cokotherium* (Wang et al., 2022). The possibility that the OMC connects either to middle ear bones or to surrounding structures via some ligaments or muscles can not be ruled out entirely in *Cokotherium*. We propose that to date, the condition observed in *Cokotherium* represents the final stage of MAME and an essential precursor of DME under the assumption that no substantial connection of OMC to the middle ear exists. This finding further refines the boundary between MAME and DME. Developmental studies suggest that the Meckelian cartilage retains the capacity of ossification and can persist with a corresponding Meckelian groove beyond juvenile stages by the knockout of chondroclasts in mutant mice (Anthwal et al., 2017). In line with the regularity of vestigial structures (Sadier et al., 2021), the presence of a Meckelian groove/sulcus in fossils implys the existence of the Meckelian cartilage in adults, whether ossified or not. As such, the condition observed in Early Cretaceous eutherian *Ambolestes* and *Prokennalestes* probably falls within the category of MAME because of the presence of a short, gracile, and posteriorly confined Meckelian sulcus. In our description and discussion, we follow the terminology discussed above.

Following Wang et al. (2021), the term “auditory bones” or “middle ear bones” used in this paper denote the ossicular chain of the middle ear, including the malleus, incus, and stapes as in extant mammals (also see discussion below).

[*…we restrict middle ear ossicles to the bones in extant mammals that are suspended within the middle ear and are components of the lever system that transmits vibrations across the middle ear, linking the tympanic membrane and the fluids of the inner ear. In extant mammals, this includes the malleus, incus, and stapes*

only; the ectotympanic, to which the tympanic membrane is attached, forms the border between the middle and outer ears. Although the ectotympanic may vibrate with the ossicular chain through the connection to the anterior process of the malleus, it is not a resident of the middle ear.]

Evolution of DME in the Mesozoic

Based on the strict consensus of parsimony analysis, the DME evolved multiple times independently in australosphenidans, allotherians (multituberculates, haramiyidans, gondwanatherians), spalacolestines, and therians in the Mesozoic era (Kielan-Jaworowska et al., 2004; Luo, 2011; Wang et al., 2019; Krause et al., 2020; Han and Meng, 2016; Wang et al., 2022; Wang et al., 2021; Kusuhashi et al., 2013). In eutherians, Early Cretaceous taxa (with the exception of *Sasayamamylos*, *Montanalestes*, *Microtherulum*, and a few specimens of *Prokennalestes*) have a short Meckelian sulcus or OMC (Cifelli, 1999; Ji et al., 2002; Luo et al., 2003; Luo et al., 2011; Kusuhashi et al., 2013; Lopatin and Averianov, 2018), suggesting that some Early Cretaceous eutherians still possess MAME, similar to their contemporary eutriconodontans and "symmetrodontans". In addition to allotherians, "symmetrodontans" are probably the most basal clade that evolved DME among Mesozoic mammaliaforms (e.g., *Lactodens* and spalacolestines) (e.g., Cifelli and Madsen, 1999; Han and Meng, 2016). Recently, it is proposed that zhangheotheriid *Origolestes* represents the initial stage of separation between the middle ear and mandible (Mao et al., 2020). Based on the updated definition of three morphotypes (PAME, MAME, and DME), it seems that the middle ear in *Origolestes* belongs to MAME by definition, regardless of how the OMC is connected to the auditory ossicles. The OMC in *Origolestes* is more similar to those in its contemporary eutriconodontans (e.g., *Liaoconodon* and *Yanoconodon*), zhangheotheriids, and spalacotheroids (e.g., *Zhangheotherium* and *Maotherium*) than to those in *Ambolestes*, *Cokotherium*, and *Microtherulum* (Wang et al., 2001; Meng et al., 2003; Luo et al., 2007; Ji et al., 2009; Luo and Manley, 2020; Wang et al., 2022).

Interpretations of the middle ear in Mesozoic mammaliaforms

The mammalian middle ear is one of the iconic topics in the evolutionary biology. One of the fundamental questions in the evolution of the middle ear is whether closely

related taxa exhibit similar morphologies in the middle ear. Given the scarcity of well-preserved fossils of these delciate structures, testing this hypothesis is challenging. Nonetheless, *Vilevolodon* provides an illustrative example, because independent scientists have examined different specimens and presented different interpretations of the middle ear in *Vilevolodon* and other euharamiyidans (see Luo et al., 2017; Meng et al., 2019; Wang et al., 2022). Two independent studies confirmed that the ectotympanic is three-pronged, which contrasts with the plate-like outline as observed in *Arboroharamiya allinhopsoni*. This finding also contrasts with an alternative interpretation that suggests the ectotympanic represents as a stylohyal (Meng et al., 2019). Similar challenges arises in identifying middle ear structures in other genera across haramiyidans, multituberculates, and other mammaliaform lineages (Han et al., 2017; Wang et al., 2022; Wang et al., 2019; Mao et al., 2020b; Zhou et al., 2019; Wang et al., 2021). Meng and Mao (2021) suspected that the incus interpreted in *Jeholbaatar* and *Vilevolodon* is "non-incus" structure because of the morphological differences of this element between *Arboroharamiya* and *Vilevolodon*, as well as between *Jeholbaatar* and *Sinobaatar*.

[*…Why is the incus identified in IMMNH-PV01699 so different from that of the holotype and its sister taxon but similar to monotremes? The possibility that it is a non-incus structure, as those interpreted in Jeholbaatar and Qishou, cannot be ruled out. This could explain why both sets of the incus and malleus were moved to that degree from their position in life and yet remain well preserved*] (Meng and Mao (2021), p. 1)

We tentatively treat the reported "surangular" as originally interpreted in *Liaoconodon* and *Origolestes*, in which the presence of the surangular can not be entirely ruled out without detailed examination of these specimens. It is noteworthy that the malleus head is not distinct both in *Liaoconodon* and *Origolestes* due to the "surangular" dorsal to the malleus body. As previously suggested (Allin, 1975; Han et al., 2017; Wang et al., 2019), we postulate that the "surangular" may have been fused to the posterolateral side of the malleus body during mammaliaform evolution if it exists. In *Microtherulum*, no supporting evidence can be found for the presence of the surangular because no observable sutures exit around the malleus head.

In terms of the incus, we maintain the interpretations in *Jeholbaatar* proposed by Wang et al. (2019), suggesting that the middle ear is different in *Jeholbaatar* and *Sinobaatar pani*. Specifically, the incus of *Jeholbaatar* exhibits a different outline, including expanding edges beyond the manubrium (or manubrial base), a nearly vertical edge, and a projection. These expanding margins and sharp morphological transformations set it apart from the “lateral process of the malleus” or “manubrial base” interpreted in *Sinobaatar pani* by Mao et al. (2020, figure 2). Aside from the malleus, there are morphological differences between *Jeholbaatar* and *Sinobaatar pani* with regard to the ectotympanic and its connection to the malleus, the outline of the “incus”, and the morphotype of the stapes. To validate these interpretations of the middle ear in multituberculates, additional evidence obtained from exceptionally preserved fossils is imperative.

Incudomallear articulation in Mesozoic mammaliaforms

Three different models have been proposed for the evolution of the incudomallear articulation (the primary jaw joint of tetrapod vertebrates) (Wang et al., 2019; Mao et al., 2020b; Wang et al., 2021). Wang et al. (2019) proposed the dichotomy of the incudomallear articulation in Mesozoic mammaliaforms, dorsoventral (abutting) contact and saddle-shaped contact, as observed in extant mammals. Based on the available evidence at that time, this model highlighted the similarity of the incudomallear articulation in multituberculates and haramiyidans (also monotremes) (Wang et al., 2019). However, this model was not aimed to address the transition between two configurations of the incudomallear articular. In contrast, Mao et al. (2020b) proposed a new model that introduced a new form of articulation known as the braced hinge joint, in addition to the dorsoventral contact (flat contact) and the saddle-shaped contact (saddle-shaped joint). The second model suggested that the braced hinge joint was primitive for Mammalia, emphasizing that the flattened condition (as observed in monotremes) evolved from the braced hinge joint through a dorsal shift of the incus, and the saddle-shaped therian joint evolved from it through a caudal shift of the incus. However, subsequent studies have raised challenges to this model (Wang et al., 2021; Wible et al., 2021).

[*Mao et al. (2020b: supplementary movie 4) provided a movie of the CT renderings that allows a 360° view of the auditory apparatus as composite and*

individual elements. The incus depicted in the movie is plate-like with a gently convex surface on the side facing the malleus and the opposite side is gently concave. Although Mao et al. (2020b) noted the resemblance to monotremes, they ultimately concluded the incudomallear articulation in Sinobaatar is most like that in Liaoconodon and Origolestes, all having a braced hinge joint using their terminology (our partially overlapping joint, Fig. 3)]. (Wang et al., 2021, Supplementary Information, pp.19).

The third model also proposed a new form of the incudomallear articulation called the partial overlapping joint, which lies between the overlapping joint and saddle-shaped joint (Wang et al., 2021). In contrast to the second model, this model suggested that the overlapping joint is the primitive condition for mammals. This debate led to further discussions on detailed investigations into the incudomallear articulation in Mesozoic mammaliaforms. However, it still goes on with different interpretations of middle ear bones in the aforementioned taxa (e.g., haramiyidan *Arboroharamiya*, *Vilevolodon*, and *Qishou*) (Meng and Mao, 2021; Wible et al., 2021). Wible et al. (2021) concluded that the primitive state of the incudomallear articulation for Mammalia remains ambiguous based on the available evidence, in contrast to the original conclusion in Wang et al. (2021). Ultimately, the prevailing consensus seems that morphological evidence is still sparse to illustrate the evolutionary history of the mammalian middle ear, as stated in the recent work:

[…*Our optimization (Fig. 1b) shows that the primitive condition for Mammalia is ambiguous, which differs from the results of Wang et al. [5] (with complete overlap as primitive) as well as Meng and Mao [6] (with partial overlap primitive in their Fig. 1o). This change from the conclusion of Wang et al. [5] is a result of the addition of scores for S. pani and removal of Qishou, which highlights how fluid such analyses are, given how few taxa are known for middle. A finding of Wang et al. [5] that we emphasize here is the similarity of the incudomallear joint in multiple lineages of Mesozoic mammals and monotremes. We do not see major distinctions between the overlapping and partial overlapping joints and believe the transformation from one to the other did not require massive overhauling, contra Meng and Mao [6].*] (Wible et al., 2021, pp.2)

[*Wang et al. postulated their hypothesis based on the less-supported result of their analysis. Under the rule of parsimony, that hypothesis (Fig. 1n) should be falsified because it requires at least five evolutionary steps in the mammalian middle ear evolution.*] (Meng and Mao, 2021, pp.3)

In addition, Wible et al. (2021) suggested the similarity of the overlapping incudomallear joint in eutriconodontans, "symmetrodontans", allotherians, and monotremes. and no major distinctions exist between the overlapping joint and the partial overlapping joint.

[*A finding of Wang et al. [5] that we emphasize here is the similarity of the incudomallear joint in multiple lineages of Mesozoic mammals and monotremes. We do not see major distinctions between the overlapping and partial overlapping joints and believe the transformation from one to the other did not require massive overhauling, contra Meng and Mao [6].*] (Wible et al., 2021, pp.2)

Microtherulum offers new evidence for evaluating the morphological differences between different categories of the incudomallear joint across mammaliaforms (Figs. 3, 5, Supplementary Figure 3). The new findings in *Microtherulum* indicate that the saddle-shaped incudomallear joint evovled in Early Cretaceous eutherians is already similar to that of extant therians. By contrast to the distinct saddle-like incudomallear articulation in therians, differences between the overlapping versus the half-overlapping incudomallear joints in monotremes and stem mammals would be relatively minor. This evidence suggests that the overlapping and partial overlapping joints probably belong to the same category, namely the overlapping joint, compared to the saddle-shaped joint that had emerged in eutherians of the Early Cretaceous. Notably, our results highlight that the thin, elongated articular surface between the malleus and incus in several Mesozoic lineages significantly differs in both morphology and function from the saddle-shaped joint. The new findings support the dichotomy of the incudomallear articulation in Mesozoic mammaliaforms. Functionally, a posterior shift of the incus would facilitate the transition from the overlapping joint to the partial overlapping joint, as proposed by Wible et al. (2021).

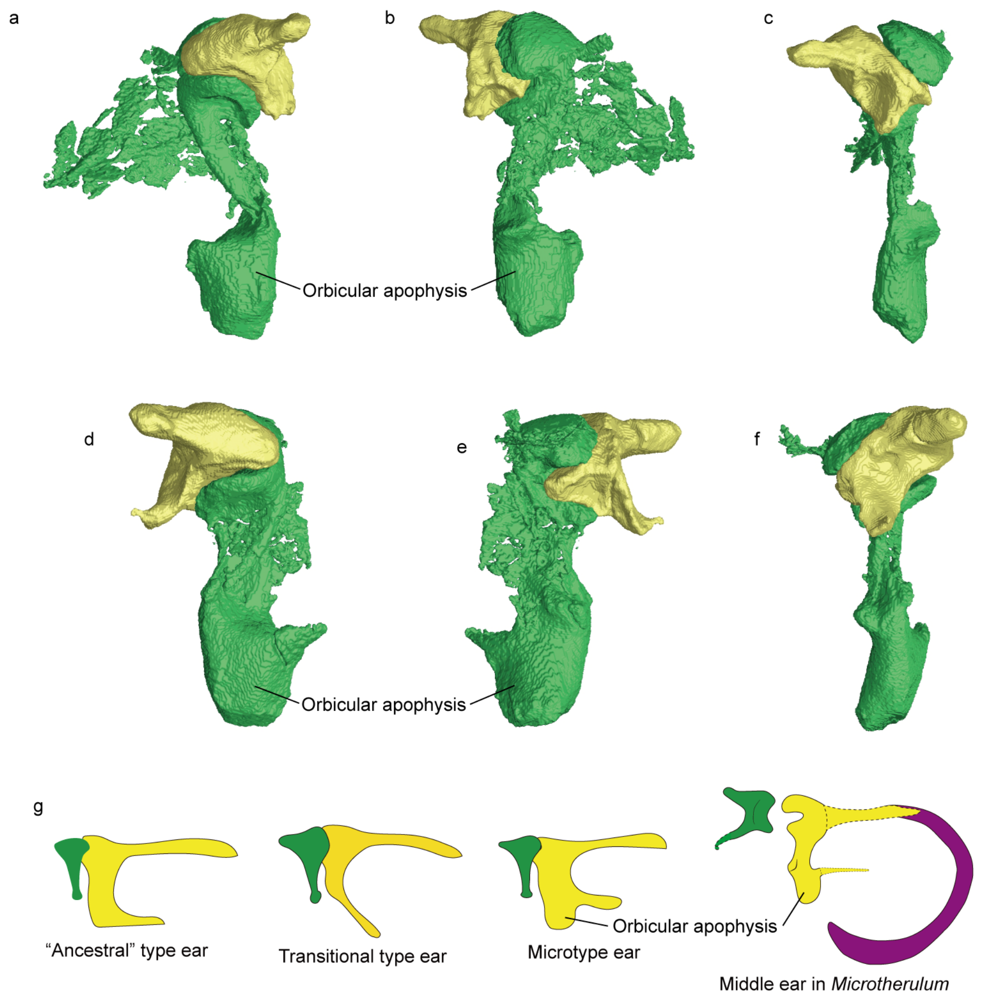

Supplementary Figure 3. The middle ear of *Microtherulum*. The left malleus and incus in ventral (a), dorsal (b), and posterior (c) views. The right malleus and incus in ventral (d), dorsal (e), and posterior (f) views. The comparison of the orbicular apophysis in different ear types (g).

Supplementary Note 2

Phylogenetic analysis

The character matrix was modified from previous studies (Han et al., 2017; Wang et al., 2019; Wang et al., 2022), and 61 characters were added to the new character list, 18 characters from Zhou et al. (2019) and 43 characters from Mao et al. (2021). All characters are modified to ensure that each phylogenetic character is an organismal feature expressed as an independent variable. Newly published mammaliaform taxa represented by well-preserved specimens were added in taxon sampling, such as *Kalaallitkigun*, *Jueconodon,* and *Cokotherium* (Sulej et al., 2020; Mao et al., 2021; Wang et al., 2022), while *Sinobaatar* was exluded. The updated character matrix for phylogenetic analysis now consisit of 615 characters and 135 OTUs. Data matrices were edited in Mesquite V. 3.03. Parsimony analysis was performed using TNT 1.5 with New Technology Search method (Goloboff, 2016), implementing sectorial search, ratchet (200 iterations), drift (100 cycles), and tree fusing (20 rounds) under equally weighted parsimony (Goloboff, 1999; Nixon, 1999; Torres et al., 2021). Parsimony analysis returned 13 MPTs, with a length of 3125, CI=0.306, RI=0.792. The length of the strict consensus is 3211, CI=0.298, RI=0.784 (Supplementary Figure 4). The length of the 50%-majority consensus is 3125, CI=0.305, RI=0.792 (Supplementary Figure 5).

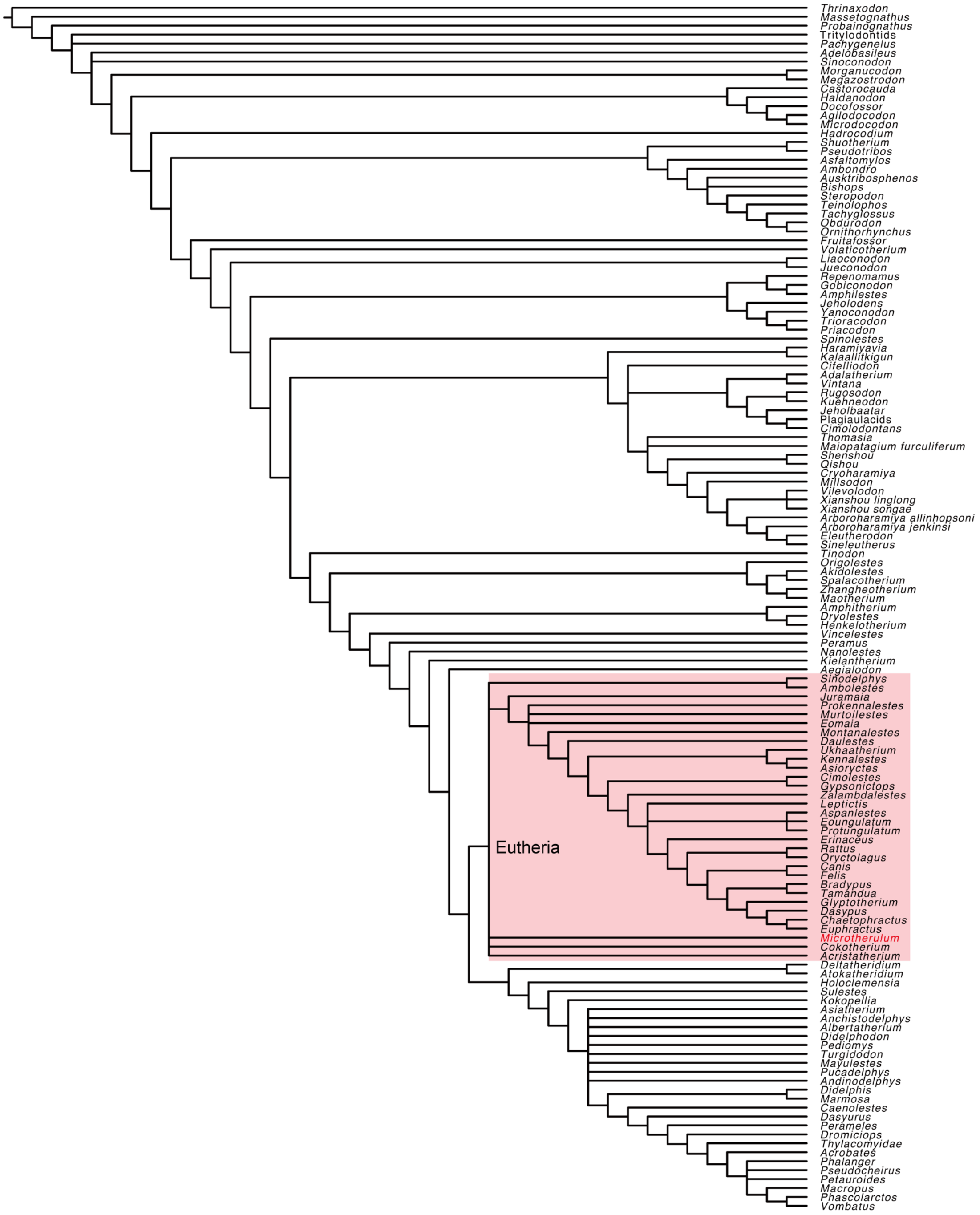

Supplementary Figure 4. Strict consensus of 13 MPTs returned from parsimony analysis.

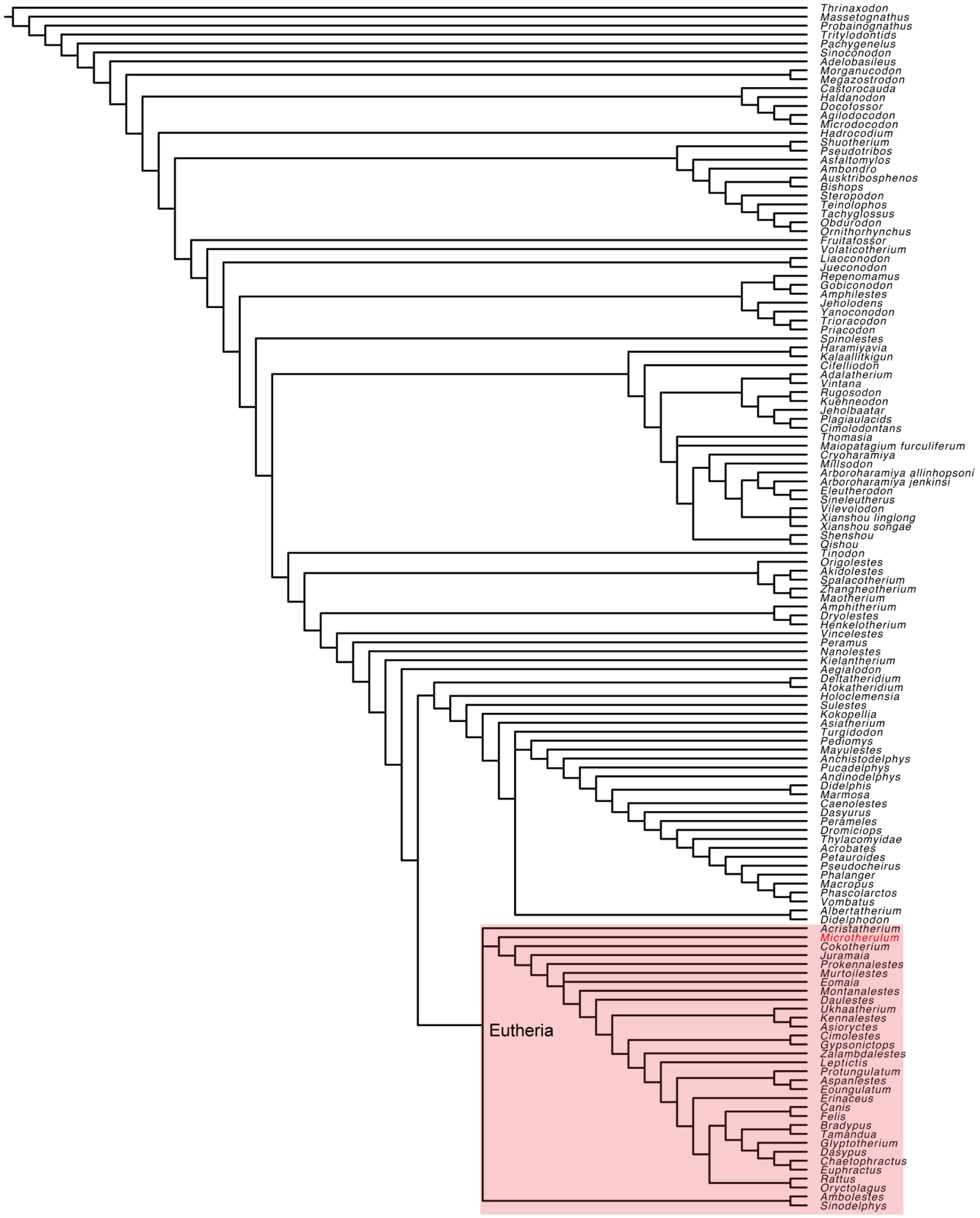

Supplementary Figure 5. The 50%-majority consensus of 13 MPTs returned from parsimony analysis.

Character list and scorings of *Microtherulum* for phylogenetic analysis in mammaliaforms

Mandible

1. Mandible, post-dentary trough (behind the tooth row), presence:

(0) Present;

(1) Absent.

Microtherulum = 1

2. Mandible, separate scars for the surangular/prearticular, presence:

(0) Present;

(1) Absent.

Microtherulum = 1

3. Overhanging medial ridge above the post-dentary trough (behind the tooth row) , presence:

(0) Present;

(1) Absent.

Microtherulum = 1

4. Mandible, well-developed Meckelian groove in adults (extending anterior to mandibular foramen) , presence:

(0) Present;

(1) Absent.

Microtherulum = 1

5. Mandible, curvature of Meckelian sulcus (under the tooth row):

(0) Parallel to the ventral border of the mandible;

(1) Convergent on the ventral border of the mandible.

Microtherulum = NA

6. Mandible, groove for the replacement dental lamina (Crompton's groove) , presence:

(0) Present;

(1) Absent.

Microtherulum = 1

7. Mandible, angular process, presence:

(0) Weakly developed to absent;

(1) Present

Microtherulum = 1

8. Mandible, angular process, inflection:

(0) Not inflected;

(1) Transversely flaring (This is different from character state (3) in having a lateral expansion of the angle and in lacking the anterior shelf)

(2) Slightly medially inflected;

(3) Strongly inflected, and continuing anteriorly as the mandibular shelf.

Microtherulum = 0

9. Mandible, angular process, position relative to the dentary condyle:

(0) Anterior position (the angular process is below the main body of the coronoid process, separated widely from the dentary condyle);

(1) Posterior position (the angular process is positioned at the level of the posterior end of the coronoid process, either close to, or directly under the dentary condyle).

Microtherulum = 1

10. Mandible, angular process, vertical elevation relative to the molar alveoli:

(0) Angular process low, at or near the level of the ventral border of the mandibular horizontal ramus;

(1) Angular process high, at or near the level of the molar alveolar line (and far above the ventral border of the mandibular horizontal ramus).

Microtherulum = 0

11. Flat ventral surface of the mandibular angle:

(0) Absent;

(1) Present.

Microtherulum = 0

12. Exoflection of the angular process of mandible, presence:

(0) Absent;

(1) Present.

Microtherulum = 0

13. Coronoid bone (or its attachment scar), development:

(0) Significant;

(1) Vestigial;

(2) Absent.

Microtherulum = 2

14. Mandibular foramen (posterior opening of the mandibular canal), location:

(0) Within the postdentary trough (or in the posterior part of Meckel's sulcus);

(1) In the pterygoid fossa and offset from Meckel's sulcus (the intersection of Meckel's sulcus at the pterygoid margin is ventral and posterior to the foramen);

(2) In the pterygoid fossa and in alignment with the posterior end of Meckel's sulcus; (3) In the pterygoid fossa but not associated with Meckel's sulcus;

(4) Not associated with any of the above structures.

Microtherulum = ?

15. Mandibular foramen, vertical position:

(0) Below the alveolar plane;

(1) At or above the alveolar plane.

Microtherulum = ?

16. Concavity (fossa) for the reflected lamina of the angular bone on the dentary, presence:

(0) Present;

(1) Absent.

Microtherulum = 1

17. Concavity (fossa) for the reflected lamina of the angular bone on the dentary, position:

(0) On the medial side;

(1) On the posterior aspect.

Microtherulum = NA

18. Splenial bone as a separate element (as indicated by its scar on the dentary), presence:

(0) Present;

(1) Absent.

Microtherulum = 1

19. Surangular bone (or associated postdentary element), contact with the squamosal, presence:

(0) Absent;

(1) Present.

Microtherulum = 0

20. Pterygoid muscle fossa on the medial side of the ramus of the mandible, presence:

(0) Absent;

(1) Present.

Microtherulum = 0

21. Medial pterygoid ridge (shelf) along the ventral border of the body of the mandible, presence:

(0) Absent;

(1) Present;

Microtherulum = 1

22. Medial pterygoid ridge (shelf) (along the ventral border of the body of the mandible), reaching the dentary condyle via a low crest, presence:

(0) Absent;

(1) Present.

Microtherulum =0

23. Masseteric fossa, ventral border, development:

(0) Absent or weakly developed;

(1) Present as a low and broad crest;

(2) Present as a well-defined and thin crest.

Microtherulum = 1

24. Crest of the masseteric fossa along the anterior border of the coronoid process, development:

(0) Absent or weakly developed;

(1) Distinctive;

(2) Hypertrophied and laterally flaring.

Microtherulum = 1

25. Anteroventral extension of the masseteric fossa, development:

(0) Absent;

(1) Extending anteriorly onto the body of the mandible;

(2) Further anterior extension below the ultimate premolar/first molar.

Microtherulum = 1

26. Labial mandibular foramen inside the masseteric fossa, presence:

(0) Absent;

(1) Present.

Microtherulum = 0

27. Masseteric fossa, posterior vertical shelf, connection to the dentary condyle, presence:

(0) Absent;

(1) Present.

Microtherulum = 0

28. Posterior-most mental foramen, position:

(0) In the canine and anterior lower premolar (premolariform) region (in the saddle behind the canine eminence of the mandible or behind incisor if canine is absent);

(1) Below the penultimate lower premolar (under the anterior end of the functional postcanine row);

(2) Below the ultimate lower premolar;

(3) At the ultimate lower premolar and the first lower molar junction;

(4) Under the first lower molar.

Microtherulum = 2

29. Mandible, articulation with the squamosal, presence:

(0) Absent;

(1) Present.

Microtherulum = 1

30. Mandible, articulation with the squamosal, condyle/glenoid, presence:

(0) Absent;

(1) Present.

Microtherulum = 1

31. Mandible, articulation, condyle, size:

(0) Small or vestigial;

(1) Massive.

Microtherulum = 1

32. Mandible, articulation, condyle, shape:

(0) Bulbous and transversely broad in its dorsal aspect;

(1) Mediolaterally narrow and vertically deep, forming a broad arc in lateral outline, either ovoid or triangular in posterior view.

Microtherulum = 1

33. Dentary peduncle (condylar process) and condyle, orientation:

(0) More posteriorly directed;

(1) Continuous with the semicircular posterior margin of the dentary; the condyle is facing up due to the upturning of the posterior-most part of the dentary;

(2) Dentary articulation extending vertically for the entire depth of the posterior mandibular ramus; it is confluent with the ramus and without a peduncle; the dentary articulation is posteriorly directed;

(3) Dentary peduncle more vertically directed.

Microtherulum = 0

34. Dentary peduncle, ventral (inferior) border, shape:

(0) Posteriorly tapering;

(1) Columnar and with a lateral ridge;

(2) Ventrally flaring;

(3) Robust and short;

(4) Ventral part of the peduncle and condyle continuous with the ventral border of the mandible.

Microtherulum = 0

35. Dentary peduncle, gracile and elongate outline, presence:

(0) Absent;

(1) Present.

Microtherulum = 0

36. Dentary condyle, position, relative to the level of the postcanine alveoli:

(0) Below or about the same level; (1) Above.

Microtherulum = 1

37. Tilting of the coronoid process of the mandible (measured as the angle between the anterior border of the coronoid process and the horizontal alveolar line of all molars):

(0) Coronoid process strongly reclined (the coronoid angle obtuse (≥150°);

(1) Coronoid process less reclined (135°-145°);

(2) Coronoid process less than vertical (110°-125°);

(3) Coronoid process near vertical (95°-105°).

Microtherulum = 2

38. Mandible, coronoid process, gracile base, presence:

(0) Absent;

(1) Present.

Microtherulum = 0

39. Mandible, coronoid process, height:

(0) Not reduced;

(1) Reduced.

Microtherulum = 0

40. Ultimate molar (or posterior-most postcanine), position, alignment to the anterior margin of the coronoid process (and near the coronoid scar if present):

(0) Medial to the coronoid process;

(1) Aligned with the coronoid process.

Microtherulum = 1

41. Mandible, movement during occlusion (as inferred from teeth), direction:

(0) Dorsal movement;

(1) Dorsomedial movement with a significant medial component;

(2) Dorsoposterior movement;

(4) Essentially horizontal movement with posterolateral translation.

Microtherulum = 1

42. Mandible, symphysis:

(0) Fused;

(1) Unfused.

Microtherulum = 1

43. Mandible, rostral mandibular spout, presence:

(0) Absent;

(1) Present.

Microtherulum = 0

44. Mandible, depth, relative to the length:

(0) Shallow;

(1) Deep.

Microtherulum = 0

Premolars:

45. Ultimate upper premolar with two rows of multiple cusps, presence:

(0) Absent;

(1) Present.

Microtherulum = 0

46. Upper ultimate and penultimate premolars, basined structure (with main cusps located peripherally surrounding a shallow and broad central basin), presence:

(0) Absent;

(1) Present.

Microtherulum = NA

This character is only scored for taxa that have postcanines with multiple rows of cusps.

47. Upper ultimate and penultimate premolars, central valley, mesial end:

(0) Open;

(1) Closed (trenched when deeply worn).

Microtherulum = NA

This character is only scored for taxa that have postcanines with multiple rows of cusps.

48. Ultimate upper premolar, width, relative to the first upper molar:

(0) Transversely narrower than, or subequal to, the first upper molar;

(1) Transversely wider than the first upper molar;

(2) Subequal in width.

Microtherulum = 0

49. Upper premolars, cusp, enamel ridges or flutings, presence:

(0) Absent;

(1) Present.

Microtherulum = 0

50. Ultimate upper premolar (with multi-rows of cusps), labial row of cuspules, presence:

(0) Absent;

(1) Present;

Microtherulum =NA

This character is only scored for taxa that have postcanine teeth with multiple rows of cusps.

51. Ultimate upper premolar, metastylar lobe, size:

(0) Reduced or absent;

(1) Enlarged and wing-like.

Microtherulum = 1

This character is only applicable to taxa with premolar-molar differentiation and is not applicable to taxa that have teeth with multiple rows of cusps.

52. Ultimate upper premolar, metacone or metaconal swelling, presence:

(0) Absent; (1) Present.

Microtherulum = 1

This character is only applicable to taxa with premolar-molar differentiation and is not applicable to taxa that have teeth with multiple rows of cusps.

53. Ultimate upper premolar, protocone or protoconal swelling, presence:

(0) Little or no lingual swelling;

(1) Present.

Microtherulum = 1

This character is only applicable to taxa with premolar-molar differentiation and is not applicable to taxa that have teeth with multiple rows of cusps.

54. Penultimate upper premolar, protocone or protoconal swelling, development:

(0) Little or no lingual swelling;

(1) Protoconal swelling;

(2) Distinctive and functional protocone.

Microtherulum = 1

This character is only applicable to taxa with premolar-molar differentiation and is not applicable to taxa that have teeth with multiple rows of cusps.

55. Upper premolar, tallest cusp within the premolar series, position:

(0) No premolar standing out;

(1) In ultimate premolar position;

(2) In penultimate premolar position.

Microtherulum = 2

This character is only applicable to taxa with premolar-molar differentiation.

56. Upper premolars, diastema posterior to the first upper molar, presence:

(0) Absent;

(1) Present.

Microtherulum = 0

This character is only applicable to taxa with premolar-molar differentiation.

57. Penultimate upper premolar, tallest cusp within longitudinal cusp row, position:

(0) Central;

(1) Tallest cusp anterior with posterior cusps (if existing) with decreasing heights;

(2) Tallest cusp on buccal row;

(3) Cusps even.

Microtherulum = 1

58. Ultimate lower premolar, hypertrophic mesial cusp, presence:

(0) Absent;

(1) Present.

Microtherulum = NA

This character is only applicable to taxa that have multi-rowed premolars.

59. Ultimate lower premolar, symmetry of the main (middle) cusp a (= protoconid), presence:

(0) Absent (anterior edge of cusp a is more convex in outline than the posterior edge);

(1) Present (anterior and posterior cutting edges are equal or subequal in length; neither edge is more convex or concave than the other in lateral profile).

Microtherulum = 1

This character is not applicable to taxa that have teeth with multiple rows of cusps.

60. Ultimate lower premolar, anterior cusp b (= paraconid), development:

(0) Indistinctive (or absent);

(1) Distinctive;

(2) Enlarged.

Microtherulum = 1

This character is not applicable to taxa that have teeth with multiple rows of cusps.

61. Ultimate lower premolar, arrangement of principal cusp a, cusp b (if present), and cusp c (assuming the cusp to be c if there is only one cusp behind the main cusp a):

(0) Aligned in a single straight line or at a slight angle;

(1) Distinctive triangulation;

(2) Premolar multicuspate in longitudinal row(s).

Microtherulum = 0

62. Ultimate lower premolar, posterior-most (distal) cingulid or cingular cuspule (in addition to cusp c or the metaconid if the latter cusp is present on a triangulated trigonid), development:

(0) Absent or indistinctive;

(1) Present;

(2) Present, in addition to cusp c or the c swelling;

(3) Presence of the continuous posterior (distal) cingulid at the base of the crown.

Microtherulum = 1

63. Ultimate lower premolar, outline:

(0) Laterally compressed (or slightly angled);

(1) Transversely wide (by trigonid);

(2) Transversely wide (by talonid);

(3) Transversely wide (by inflated anterior cusp and/or distal basined heel).

Microtherulum = 0

64. Posterior upper premolar, single enlarged anterior (mesial) sectorial cusp, presence:

(0) Absent;

(1) Present.

Microtherulum = 0

65. Penultimate or ultimate lower premolar, carnassial shearing notch in the middle of the tooth, presence:

(0) Absent;

(1) Present.

Microtherulum = 1

66. Lower premolars, basined heel, development:

(0) Indistinctive or absent;

(1) Weakly developed;

(2) Full molarization of posterior premolars.

Microtherulum = 0

67. Lower premolars, distinctive cingulid with cuspules or crenulated cingulid, presence:

(0) Absence;

(1) Present;

Microtherulum = 0

68. Lower premolars, distinctive cingulid with cuspules or crenulated cingulid, topographic relation to the main cusp row:

(0) Labially positioned;

(1) Lingually positioned.

Microtherulum = NA

69. Ultimate lower premolar, labial cingulid, development:

(0) Absent or vestigial;

(1) At least along the length of more than half of the crown);

(2) Cuspate distal cingulid.

Microtherulum = 0

70. Ultimate lower premolar, lingual cingulid, development:

(0) Absent or vestigial;

(1) Present.

Microtherulum = 0

71. Ultimate lower premolar, height of primary cusp a relative to cusp c (measured as the height ratio of a and c from the bottom of the valley between the two adjacent cusps):

(0) Indistinctive;

(1) Posterior cusp c distinctive but less than 30% of the primary cusp a;

(2) Posterior cusp c and primary cusp a equal or subequal in height (c is 40%-100% of a).

Microtherulum = 0

72. Penultimate lower premolar, paraconid (=cusp b), development:

(0) Absent;

(1) Present but not distinctive;

(2) Distinctive and slightly enlarged.

Microtherulum = 1

73. Penultimate lower premolar, principal cusp a, cusp b (if present), and cusp c (we assume the cusp to be c if there is only one cusp behind the main cusp a), arrangement:

(0) Individual cusps in straight alignment (for a tooth with a single cusp, the anterior and posterior crests from the main cusp are in alignment):

(1) Cusps in reversed triangulation;

(2) With multicusps or multi-serrations in a single longitudinal row;

(3) With multicusps or multi-serrations rows.

Microtherulum = 0

74. Penultimate lower premolar, labial cingulid, presence:

(0) Absent;

(1) Present.

Microtherulum = 0

75. Posterior premolars, elongation:

(0) Absent;

(1) Present.

Microtherulum = 0

76. Upper molars, the mesial U-ridge, closed by cuspules:

(0) Absent;

(1) Present;

Microtherulum = NA

This character is scored for taxa with multi-rows of cusps.

77. Upper molars, cuspules and/or transverse fluting of the central basin, presence:

(0) Absent; (1) Present.

Microtherulum = NA

78. Upper molars, cusp A1, position:

(0) A1 is at the same level as B1;

(1) A1 is distal to B1.

Microtherulum = NA

This character is only scored for those with multi-rows of multi-cusps.

79. M1, cusp formula (A row relative to B row):

(0) 4:4 or lower;

(1)5:4;

(2) 6:4 or higher.

Microtherulum = NA

80. Anterior lower molars, alignment of the main cusps (justification for separating this feature from the next character on the list: several taxa of "obtuse-angled symmetrodonts" and eutriconodont amphilestids show a gradient of variation in cusp triangulation along the molar series; the degree of triangulation may be different between the anterior and posterior molars):

(0) Single longitudinal row;

(1) Reversed triangle–acute (≤90°);

(2) Two or more longitudinal multicuspate rows.

Microtherulum = 1

81. Posterior lower molars, cusp triangulation:

(0) Absent;

(1) Multi-row (primarily two rows) and multi-cuspate;

(2) Present;

Microtherulum = 2

82. Posterior lower molars, triangulation of cusps, development:

(0) Posterior molars slightly triangulated;

(1) Posterior molars fully triangulated.

Microtherulum = 1

83. Upper molar, B1 cusp, presence:

(0) Absent;

(1) Present.

Microtherulum = 0

This character is only applicable to taxa that have molars with triangulation.

84. Postvallum/prevallid shearing (based on the second lower molar), presence:

(0) Absent;

(1) Present.

Microtherulum = 1

85. Postvallum/prevallid shearing, angle of the main trigonid shear facets (based on the second lower molar):

(0) Slightly oblique;

(1) More transverse.

Microtherulum = 1

86. Postvallum/prevallid shearing, shear facets, development (based on the second lower molar):

(0)Weakly developed;

(1) strongly developed;

Microtherulum = 1

87. Rank of postvallum shear (on the upper second molar) (increasing the ranks of postvallum shear and can be ordered):

(0) Present but only by the first rank: postmetacrista;

(1) Present, with the addition of a second rank (postprotocrista below postmetacrista) but the second rank doesnot reach labially below the base of the metacone;

(2) Metacingulum/metaconule present, in addition to postprotocrista, but the metacingulum crest does not extend beyond the base of the metacone;

(3) Metacingulum extended beyond metacone;

(4) Metacingulum extended to the metastylar lobe;

(5) Second rank postvallum shear forming a broad shelf (as in selenodonty).

Microtherulum = 3

This character is only applicable to molars with reversed triangulation of cusps.

88. Postcingulum, presence:

(0) Absent or weak;

(1) Present;

Microtherulum = 0

89. Postcingulum, development:

(0) Short;

(1) Reaching past the metaconule;

(2) Formed by the hypoconal shelf raised to near the level of the protocone;

Microtherulum = NA

90. Upper and lower molars, precise opposition, presence:

(0) Absent;

(1) Present.

Microtherulum = 1

91. Upper and lower molars, precise opposition, form:

(0) Either one-to-one, or occluding at the opposite embrasure or talonid;

(1)) One lower molar contacts sequentially more than one upper molar;

Microtherulum = 0

92. Relationships between the cusps of the opposing upper and lower molars, presence:

(0) Absent;

(1) Present.

Microtherulum = 1

93. Relationships between the cusps of the opposing upper and lower molars:

(0) Lower primary cusp a occludes in the groove between upper cusps A, B;

(1) Lower main cusp a occludes in front of the upper cusp B and into the embrasure between the opposite upper tooth and the preceding upper tooth;

(2) Parts of the talonid occluding with the lingual face (or any part) of the upper molar;

(3) Lower multicuspate rows alternately occluding between the upper multicuspate rows;

(4) Columnar tooth without cusps and with beveled wear across the entire crown contact surface.

Microtherulum = 2

94. Lower m1 with multicuspate rows, lingual row occlude into the basin of upper molar, presence:

(0) Absent; (1) Present.

Microtherulum = NA

95. Lower m2 with multicuspate rows, the lingual cusp row occlude into the basin of upper molar, presence:

(0) Absent; (1) Present

Microtherulum = NA

96. Lower molars with multi-rows of cusps, distal end:

(0) Open;

(1) Closed by the ridge;

(2) Closed by the cuspules.

Microtherulum = NA

97. Lower molars, cuspules or ridges of the central basin, presence:

(0) Absent; (1) Present.

Microtherulum = NA

98. Lower molars, fusiform ("spindle-shaped") shearing valley between lingual cusp row and labial cusp row, presence:

(0) Absent; (1) Present.

Microtherulum = NA

99. m1, main lingual row cusp (distribution revised), number:

(0) 4 or fewer; (1) 5; (2) 6 or more.

Microtherulum = NA

100. Lower molar (molariform), cusp shape:

(0) Conical;

(1) Pyramidal;

(2) Symmetrically crescent (with distal face concave);

(3) Asymmetrically crescent (with distal face concave);

(4) Cusp strongly crested;

(5) Cusp lost.

Microtherulum = 0

101. Lower molars, protoconid (cusp a) and metaconid (cusp c), height ratio (on the lower second molar):

(0) Protoconid distinctively higher;

(1) Protoconid and metaconid nearly equal in height.

Microtherulum = 0

102. Lower molars, paraconid (cusp b) and metaconid (cusp c), base, relative size (on the lower second molar):

(0) Paraconid distinctively higher than the metaconid;

(1) Paraconid and metaconid nearly equal in height;

(2) Paraconid lower than metaconid;

(3) Paraconid reduced or absent.

Microtherulum = 2

103. Lower molars, paraconid (cusp b), elevation of the cingulid base, relative to the cingulid base of the metaconid (cusp c), presence:

(0) Absent; (1) Present.

Microtherulum = 0

104. Lower molars, cristid obliqua (sensu Fox, 1975: defined as the oblique crest anterior to, and connected with, the labial-most cusp on the talonid heel, the leading edge of facet 3), presence:

(0) Absent;

(1) Present.

Microtherulum = 1

This character is applicable only to the molar with at least a hypoconid on the talonid or a distal cingulid cuspule.

105. Lower molars, cristid obliqua (sensu Fox, 1975: defined as the oblique crest anterior to, and connected with, the labial-most cusp on the talonid heel, the leading edge of facet 3), orientation:

(0) Contact closest to the middle posterior of the metaconid;

(1) Contact closest to the lowest point of the protocristid;

(2) Contact closest to the middle posterior of the protoconid

Microtherulum = 0

106. Lower molars, talonid heel, medial and longitudinal crest (=‘pre-entocristid’ or ‘pre-hypoconulid’):

(0) Talonid (or cusp d) has no medial and longitudinal crest;

(1) Medial-most cristid (‘pre-entoconid cristid’) of the talonid in alignment with the metaconid or with the post-metacristid if the latter is present (the postmetacristid is defined as the posterior crest of metaconid that is parallel to the lingual border of the crown), but widely separated from the latter;

(2) Medial-most cristid of the talonid (‘pre-hypoconulid’ cristid, based on cusp designation of Kielan-Jaworowska et al., 1987) is hypertrophied and in alignment with the postmetacristid and abuts the latter by a V-notch;

(3) ‘Pre-entocristid’ crest is offset from the metaconid (and postmetacristid if present), and the ‘pre-entocristid’ extending anterolingually past the base of the metaconid.

Microtherulum = 1

This character is only applicable to taxa with talonid or at least a cusp d.

107. Lower molars/molariforms, posterior lingual cingulid, development:

(0) Absent or weak;

(1) Distinctive;

(2) Strongly developed, crenulated with distinctive cuspules (such as the kuhneocone).

Microtherulum = 0

108. Lower molars/molariforms, anterior internal (mesio-lingual) cingular cuspule (e), presence:

(0) Present;

(1) Absent.

Microtherulum = 0

109. Lower molars/molariforms, anterior internal (mesio-lingual) cingular cuspule (e), development:

(0) As an anterior cuspule but not at the cingulid level;

(1) At the cingulid level;

(2) Positioned above the cingulid level;

(3) Hypertrophied cusp e = pseudohypoconulid;

Microtherulum = 0

110. Lower molars/molariforms, anterior and labial (mesio-buccal) cingular cuspule (f):

(0) Absent;

(1) Present;

(2) Hypertrophied to form pseudo-hypoconid.

Microtherulum = ?

111. Lower molars/molariforms, mesial cingulid above the gum, development:

(0) Absent;

(1) Weak and discontinuous, with individualized cuspules below the trigonid
(as individual cuspule e, f, or both, but e and f are not connected);

(2) Present, in a continuous shelf below the trigonid (with no relations to the protoconid and paraconid), without occlusal function;

(3) Present, with occlusal contact to the upper molar.

Microtherulum = 1

112. Lower molars/molariforms, cingulid shelf, wrapping around the anterolingual corner of the molar to extend to the lingual side of the trigonid below the paraconid, presence:

(0) Absent;

(1) Present.

Microtherulum = 0

113. Lower molars/molariforms, cingulid shelf, wrapping around the anterolingual corner of the molar to extend to the lingual side of the trigonid below the paraconid, occlusal function to the upper molars; presence:

(0) Absent;

(1) Present.

Microtherulum = NA

114. Lower molars/molariforms, postcingulid (distal transverse cingulid above the gum level), presence:

(0) Absent;

(1) Present (horizontal above the gum level).

Microtherulum = 0

115. Lower molars/molariforms, interlocking, presence:

(0) Absent; (1) Present:

Microtherulum = 1

116. Lower molars, interlocking mechanisms, morphotype:

(0) Posterior cingular cuspule d (or the base of the hypoconulid) of the preceding molar fits in between cingular cuspules e and f of the succeeding molar;

(1) Posterior cingular cuspule d fits between cingular cuspule e and cusp b of the succeeding molar;

(2) Posterior cingular cuspule d or cingulum of the preceding molar fits into an embayment or vertical groove of the anterior aspect of the succeeding molar (without any involvement of distinctive cingular cuspules in interlocking);

(3) Anterior corner of succeeding lower molar overlapping posterior corner of preceding lower molar.

Microtherulum = 1

117. Last three lower postcanines, size ratio:

(0) Ultimate molar is smaller than the penultimate molar (m1≥m2≥m3; or m2≥m3≥m4; or m3≥m4≥m5; or m4≥m5≥m6; or p4≥m1≥m2);

(1) Penultimate molar is the largest of the molars (m1≤m2≤m3≥m4; or m1≤m2>m3);

(2) Ultimate molar is larger than the penultimate molar (m1≤m2≤m3);

(3) Equal size.

Microtherulum = 1

118. Lower molars, paraconid, position relative to the other cusps of the trigonid (based on the lower second molar):

(0) Paraconid in anterolingual position;

(1) Paraconid lingually positioned (within lingual 1/4 of the trigonid width);

(2) Paraconid lingually positioned and appressed to the metaconid;

(3) Paraconid reduced in the selenodont/lophodont patterns.

Microtherulum = 1

119. Lower molars/molariforms, paracristid (or the crest between cusps a and b), orientation relative to the longitudinal axis of the molar (This is separated from the previous character [“lingual” vs. “labial” position of the paraconid] because of the different distribution of the a-b crest among mammals with non-triangulated molars sampled here):

(0) Longitudinal orientation;

(1) Oblique;

(2) Nearly transverse.

Microtherulum = 1

120. Lower molars, angle of the paracristid (b-a crest) and the protocristid (a-c crest):

(0) > 90°;

(1) 90°~ 50°;

(2) < 35°.

Microtherulum = 2

121. Lower molars, paraconid, mesiolingual vertical crest:

(0) Rounded;

(1) Forming a keel.

Microtherulum = 0

This character is applicable only to taxa with reversed triangulation of the molar cusps.

122. Lower molars, trigonid, anteroposterior shortening at the base of the trigonid relative to the talonid:

(0) Trigonid long (extending over 3/4 of the tooth length);

(1) Swelling on the side walls of the trigonid (taxa assigned to this character state have a trigonid length ratio 45%~50%; but their morphology is different from all other states in that their side walls are convex);

(2) No shortening (trigonid 50-65% of tooth length);

(3) Some shortening (the base of trigonid < 50% of tooth length);

(4) Anteroposterior compression of trigonid (trigonid 40~45% of the tooth length).

Microtherulum = 2

This character is applicable only to taxa with a talonid heel with a distal cusp d; measured at the lingual base of the lower second molar trigonid where possible.

123. Lower molars, width ratio of trigonid/talonid heel (based on the lower second molar measured where possible):

(0) Narrow (talonid ≤40% of trigonid);

(1) Wide (talonid is 40-70% of the trigonid in width);

(2) Talonid is equal or wider than trigonid.

Microtherulum = 1

124. Lower molars, hypoflexid (concavity anterolabial to the hypoconid or cusp d), development:

(0) Absent or shallow (all "triconodont-like" teeth are coded as "0" here as long as they have cuspule d);

(1) Deep (40~50% of talonid width);

(2) Very Deep (>65%);

(3) Pseudohypoflexid (40% to 65% of the pseudo-talonid width).

Microtherulum = 1

125. Lower molars, talonid (or the posterior heel), presence:

(0) Absent;

(1) Present.

Microtherulum = 1

126. Lower molars, talonid (or the posterior heel), development:

(0) As an incipient heel, a cingulid, or cingular cuspule (d);

(1) As a transverse ‘V-shaped’ basin with two functional cusps;

(2)As an obtuse ‘V-shaped’ triangle;

(3) As a basin (rimmed with 3 functional cusps with at leas is a functional crest to define the medial rim of the basin if the entoconid is not already present) with wear occurs only crests but absent from the bottom of the basin (following Martin and Rauhut 2005);

(4) As a functional basin (rimmed by 3 cusps) with wear occurs inside the basin.

Microtherulum = 4

127. Lower molars, talonid, hypoconid (we designate the distal cingulid cuspule d as the homolog to the hypoconid in the teeth with linear alignment of the main cusps; we assume the cusp to be the hypoconid if there is only a single cusp on the talonid in the teeth with reversed triangulation), development:

(0) Present, but not elevated above the cingulid level;

(1) Present (as distal cusp d, *sensu* Crompton, 1971), elevated above the cingulid level, labially positioned (or tilted in the lingual direction);

(2) Present (larger than cusp d, with occlusal contact to the upper molar), elevated above the cingulid level, labially positioned.

Microtherulum = 2

128. Lower molars, talonid, hypoconulid (if there are only two functional cusps on the talonid, we assume that the second and more lingual cusp on the talonid to be the hypoconulid, following the rationale of Kielan-Jaworowska et al., 1987), presence:

(0) Absent;

(1) Present.

Microtherulum = 1

129. Lower molars, talonid, hypoconulid (if there are only two functional cusps on the talonid, we

assume that the second and more lingual cusp on the talonid to be the hypoconulid, following the rationale of Kielan-Jaworowska et al., 1987), position:

(0) Median (near the mid-point of the transverse talonid width)

(1) Placed within the lingual 1/3 of the talonid basin;

(2) Incorporated into the crest of lophodont or selenodont conditions.

Microtherulum = 0

130. Anterior lower molar (preferably the first, or the second if the first is not available), hypoconulid, anteroposterior orientation: procumbent vs. reclined:

(0) Cusp tip reclined and the posterior wall of the hypoconulid is slanted and overhanging the root;

(1) Cusp tip procumbent and the posterior wall of the cusp is vertical;

(2) Cusp tip procumbent and the posterior wall is gibbous.

Microtherulum = 0

This character is applicable to the taxa with at least two cusps on the talonid.

131. Lower molars, hypoconulid, labial postcingulid (shelf) (definition following Cifelli, 1993; non-homologous with the postcingulid coded elsewhere in this list because of the different relationship to the talonid cusps;):

(0) Absent;

(1) Present (as a crest descending mesiolabially from the apex of the hypoconulid to the base of the hypoconid).

Microtherulum = 0

This character is applicable to taxa with identifiable hypoconid and hypoconulid only.

132. Last lower molar, hypoconulid, orientation:

(0) Erect;

(1) Recurved.

Microtherulum = 0

This character is applicable to the taxa with at least a talonid heel; scored on the third molar for *Peramus* and eutherians, the fourth molar for *Kielantherium* and metatherians; justification for separating this character from the character of the anterior molar hypoconulids is that the ultimate

molar shows different morphology and distribution, especially in taxa in which there is posteriorly decreasing size gradient, e.g. *Deltatheridium*)

133. Last lower molar, hypoconulid, size:
(0) Short;
(1) Tall (higher than hypoconid).
Microtherulum = 0

134. Lower molars, talonid, entoconid (if there are three functional cusps on the talonid, we assume that the third and the lingual-most functional cusp on the talonid is the entoconid, following the rationale given by Kielan-Jaworowska et al., 1987), presence:
(0) Absent;
(1) Present.
Microtherulum = 1

135. Lower molars, talonid, entoconid (if there are three functional cusps on the talonid, we assume that the third and the lingual-most functional cusp on the talonid is the entoconid, following the rationale given by Kielan-Jaworowska et al., 1987), position:
(0) About equal distance to the hypoconulid as to the hypoconid;
(1) With slight approximation to the hypoconulid (distance between the hypoconulid and entoconid noticeably shorter than between the hypoconulid and hypoconid);
(2) Twinned with the hypoconulid.
Microtherulum = 1

136. Lower molars, height ratio of the medial side of the crown (apex of the hypoconid to the base of the labial crown) vs. the most lingual cusp on the talonid to the base of the labial crown:
(0) Entoconid absent on the talonid heel;
(1) Entoconid lower than the hypoconid;
(2) Entoconid near the height of the hypoconid;
(3) Entoconid near the height of the hypoconid and linked to the hypoconid by a transverse crest.
Microtherulum = 1
This character can be based either on the entoconid if the entoconid is present or the hypoconulid if the entoconid cannot be scored.

137. Lower molars, paraconid, metaconid, and entoconid, alignment:

(0) Cusps not aligned;

(1) Cusps aligned.

Microtherulum = 0

This character is applicable only to taxa with triangulation of the trigonid cusps and the entoconid present on the talonid.

138. Lower molars, functional talonid basin, ratio of length vs. width (in occlusal view, measured at the cingulid level, and based on the second molar):

(0) Longer than wide (or narrows posteriorly);

(1) Length equals width;

(2) Wider than long.

Microtherulum = 1

139. Lower molars, talonid basin, elevation (measured as the height of the hypoconid from the cingulid on the labial side of the crown) relative to the trigonid (measured as the height of protoconid from the cingulid):

(0) Hypoconid/protoconid height ratio less than 20% (hypoconid or cusp d is on the cingulid);

(1) Hypoconid/protoconid height ratio between 25% and 35% (talonid cusp elevated above the cingulid level);

(2) Hypoconid/protoconid height ratio between 40% and 60%;

(3) Hypoconid/protoconid height ratio between >60% and 80%;

(4) Equal height.

Microtherulum = 1

This character is applicable only to the teeth with reversed triangulation.

140. Penultimate upper molar, labial stylar shelf, presence:

(0) Absent;

(1) Present.

Microtherulum = 1

141. Penultimate upper molar, labial stylar shelf, size (labiolingual width):

(0) Narrow;

(1) Broad.

Microtherulum = 1

This character is scored state (1) for Jehol eutherians, *Acristatherium*, *Ambolestes*, *Cokotherium*, and *Microtherulum*.

142. Upper molars, ectoflexus, gradient along the molar series:

(0) Present on penultimate molar, but weakly developed or absent on the anterior molars;

(1) Present on the penultimate and preceding molars.

Microtherulum = 1

This character is scored (1) for *Juramaia* based on Luo et al. (2011).

143. Upper molars, labial cingulum or stylar shelf (excluding the parastyle and metastyle), morphology:

(0) Indistinctive;

(1) Distinctive cingulum, without cuspules;

(2) Individualized or evenhypertrophied cuspules;

(3) W-pattern on stylar shelf;

(4) Cingulum crenulated with distinctive and even-sized multiple cuspules.

Microtherulum = 2

144. Upper molars, protocone, presence:

(0) Functional cusp and lingual swelling absent;

(1) Functional cusp present.

Microtherulum = 1

145. Upper molars, protocone, lingual swelling, presence:

(0) Absent;

(1) Present;

Microtherulum = NA

146. Upper molars, protocone, labial shift, position (distance from the protocone apex to the lingual border vs. the total tooth width, in %):

(0) Protocone present but no labial shift (10%-20%);

(1) Moderate labial shift (25%-30%);

(2) Substantial labial shift (≥ 40%).

Microtherulum = 0

This character is applicable only to those taxa with reversed triangulation.

147. Upper molars, protocone region, apical portion, morphology:

(0) Anteroposteriorly compressed;

(1) Slightly expanded;

(2) Expanded;

(3) Forming an obtuse triangle with the protoconal cristae;

Microtherulum = 1

This character is applicable only to those taxa with reversed triangulation and a protocone of the upper molar)

148. Upper molars, protocone, height relative to the paracone and metacone (whichever is highest of the latter two):

(0) Protocone markedly lower (less than 70%);

(1) Protocone of intermediate height (70%~80%);

(2) Protocone near the height of paracone and metacone (within 80%).

Microtherulum = 1

149. Upper molars, paracone (cusp B) and metacone (cusp C) (based on the upper second molar if available), height:

(0) Paracone noticeably higher and larger at the base than metacone;

(1) Paracone slightly larger than metacone;

(2) Paracone and metacone of equal size or paracone lower than metacone.

Microtherulum = 1

150. Upper molars, metacone (cusp C), position relative to paracone (cusp B):

(0) Metacone labial to paracone;

(1) Metacone about the same level as paracone;

(2) Metacone lingual to paracone.

Microtherulum = 1

151. Upper molars, base of the paracone and metacone (based on the upper second molar if available):

(0) Merged; (1) Separated.

Microtherulum = 0

This character is applicable only to triangulated molars.

152. Upper molars, centrocrista between the paracone and the metacone, shape:

(0) Straight;

(1) V-shaped, with labially directed postparacrista and premetacrista.

Microtherulum = 0

This character is applicable only to taxa with well-developed metacone and distinctive wear facets 3 and 4.

153. Upper molars, conular region (with or without conules), anteroposterior width:

(0) Narrow (anteroposterior distance medial to the paracone and metacone less than 0.30 of total tooth length);

(1) Moderate development (distance between position of conules = 0.31—0.50 of total tooth length);

(2) Wide (distance between conules greater than 0.51 of total tooth length);

(3) Expanded.

Microtherulum = 1

This character is applicable only to taxa with reversed triangulation and an occluding lingual portion of the upper molar; for the taxa with conules, this is measured between the paraconule and metaconule; for those taxa without conules, this is measured as the length of the tooth medial to the base of paracone; the upper second molar measured where possible).

154. Upper molars, paraconule and metaconule, presence:

(0) Absent; (1) Present.

Microtherulum = 1

155. Upper molars, paraconule and metaconule on the upper first and second molars, position:

(0) Paraconule and metaconule closer to the protocone;

(1) Both positioned near the midpoint of the protocone-metacone;

(2) Paraconule and metaconule labial to the midpoint.

Microtherulum = 0

156. Upper molars, internal conular cristae (conular wing):

(0) Cristae indistinctive;

(1) Cristae distinctive and wing-like.

Microtherulum = 0

157. Upper molars, parastylar groove (on the upper second molar):

(0) Weak or absent;

(1) Moderately to well developed.

Microtherulum = 1

158. Upper molars, stylar cuspule “A”, the parastyle (of the Bensley-Simpson system; cuspule “E” of the Crompton designation), presence:

(0) Present (at least a swelling is present);

(1) Absent.

Microtherulum = 0

159. Upper molars, first molar, preparastyle, presence:

(0) Absent;

(1) Present.

Microtherulum = 1

This character is applicable to molars with triangulation.

160. Upper molars, stylar cuspule “B” (stylocone, opposite the paracone) (based on the upper second molar if available), development:

(0) Vestigial to absent;

(1) Small but distinctive;

(2) Subequal to the parastyle;

(3) Large (subequal to parastyle), with an extra "B-1" cuspule in addition to "B".

Microtherulum = 2

This character is scored state (2) for *Ambolestes*, *Cokotherium*, and *Microtherulum.*

161. Upper molars, penultimate upper molar, stylar cuspule "C" (mesostyle, near the ectoflexus), presence:

(0) Absent;

(1) Present.

Microtherulum = 0

162. Upper molars, penultimate upper molar, stylar cuspule "D" (stylar cusp D, opposite the metacone), presence:

(0) Absent;

(1) Present.

Microtherulum = 1

163. Upper molars, penultimate upper molar, stylar cuspule “E” (Bensley-Simpson designation; not the Crompton cusp E), presence:

(0) Absent or poorly developed;

(1) Present.

Microtherulum = 1

164. Upper molars, penultimate upper molar, stylar cuspule “E” (Bensley-Simpson designation; not the Crompton cusp E), size:

(0) Less developed than or subequal to stylar cuspule “D”;

(1) Better developed than cuspule “D”;

Microtherulum = 0

165. Upper molars, stylar cuspule “E”, position, relative to cusp “D” or “D-position”:

(0) “E” more lingual to “D” or “D-position”;

(1) “E” distal to or at same level as “D” or “D-position”.

Microtherulum = 0

166. Upper molars, metastylar lobe and parastylar lobe (based on the upper first molar if available;

if not, then based on upper second), size:

(0) Metastylar lobe smaller than the parastylar lobe;

(1) Metastylar lobe of similar size and labial extent to the parastylar lobe;

(2) Metastylar lobe much larger than the parastylar lobe;

(3) Metastylar lobe absent.

Microtherulum = 0

167. Upper molars, salient postmetacrista, development:

(0) Absent or weakly developed;

(1) Well-developed but no longer than the metacone-protocone distance;

(2) Hypertrophied and longer than the metacone protocone distance.

Microtherulum = 1

This character is applicable to taxa with reversed triangulation.

168. Upper molars, selenodont molar pattern, presence:

(0) Absent;

(1) Present.

Microtherulum = 0

169. Lower first molar, crown, outline (in crown view):

(0) Laterally compressed;

(1) Oblong with slight labial bulge;

(2) Triangular or tear-drop shaped;

(3) Rectangular (or rhomboidal);

(4) Circular.

Microtherulum = 1

170. Lower second molar, crown, outline (in crown view):

(0) Laterally compressed;

(1) Oblong with slight labial bulge;

(2) Triangular or tear-drop shaped;

(3) Rectangular (or rhomboidal);

(4) Circular.

Microtherulum = 1

171. Upper first molar, outline:
(0) Laterally compressed;
(1) Longer than transversely wide (oval-shaped or spindle shaped);
(2) Transversely wider than long (triangular outline);
(3) Rectangular or nearly so;
(4) Circular.
Microtherulum = 2

172. Premolar and molar, carnassial shearing blades on posterior aspect of the ultimate upper premolar and anterior aspect of the first lower molar, presence:
(0) Absent; (1) Present.
Microtherulum = 0

173. Upper molars, interlocking, presence:
(0) Absent; (1) Present.
Microtherulum = 1

174. Anterior molars, interlocking, morphotype:
(0) Notch interlock (with cingular cusps involved or without);
(1) Tongue-in-groove interlock;
(2) Parastylar lobe of a succeeding molar lumbricated with the metastylar region of a preceding molar.
Microtherulum = 2

175. Posterior molars, interlocking, morphotype:
(0) Posterior end of preceding molar lumbricating anterolabial side of ultimate upper molar;
(1) Parastylar lobe of a succeeding molar lumbricated with the metastylar region of a preceding molar;
(2) Tongue-in-groove interlock.
Microtherulum = 1

Molar Wear Pattern:

176. m1, lingual side of lingual cusps, wear facets, presence:

(0) Absent; (1) Present.

Microtherulum = ?

177. M2, buccal side, wear facets:

(0) On all buccal cusps;

(1) On buccal side of A1, but not on the buccal side of the mesiobuccal cusp.

Microtherulum = ?

178. Molars/molariforms, cusps, functional development of occlusal facets:

(0) Absent;

(1) Absent at eruption but developed later by crown wear;

(2) Present, wear facets match upon tooth eruption (inferred from the flat contact surface upon eruption).

Microtherulum = 2

179. Molars/molariforms, main cusps, topographic relationships of wear facets:

(0) Wear pattern across the entire crown;

(1) Lower cusps a, c support two different wear facets (facets 1 and 4) that contact the upper primary cusp A;

(2) Lower cusps a, c support a single wear facet (facet 4) that contacts the upper primary cusp B (this facet extends onto cusp A as wear continues, but 1 and 4 do not develop simultaneous in these taxa);

(3) Multicuspate series, each cusp may support 2 wear facets.

Microtherulum = 2

180. Upper or lower molars, prevallum/postvallid shearing (based on either upper or the lower molar structures), presence:

(0) Absent;

(1) Present.

Microtherulum = 1

181. Upper or lower molars, prevallum/postvallid shearing (based on either upper or the lower molar structures), orientation:

(0) Obtuse;

(1) Hypertrophied and transverse;

Microtherulum = 1

182. Wear facet 1 (a single facet supported by cusp a and cusp c) and facet 2 (a single facet supported by cusp a and cusp b), presence:

(0) Absent; (1) Present.

Microtherulum = 1

183. Upper molars, facet 1 and the preprotocrista, development:

(0) Facet 1 (prevallum crest) short, not extending to the stylocone area;

(1) Facet 1 extending into the hook-like area near the stylocone;

(2) Preprotocrista long, extending labially beyond the paracone.

Microtherulum = 2

This character is applicable to molars with reversed triangulation.

184. Wear facet 3 and facet 4, differentiation:

(0) Absent;

(1) Present;

Microtherulum = 1

185. Wear facets 3 and 4, hypertrophied on the flanks of the strongly V-shaped talonid, presence:

(0) Absent;

(1) Present;

Microtherulum = 0

186. Waer facet 4 (on the posterior aspect of the hypoconid), orientation:

(0) Oblique to the long axis of the tooth;

(1) Forming a more transverse angle to the long axis of the tooth.

Microtherulum = 0

187. Lower molars, talonid, posterolateral aspect (the labial face of the hypoconid or equivalent area of Crompton facet 4), shape:

(0) Gently rounded;

(1) Angular.

Microtherulum = 0

This character is applicable to taxa with fully basined talonid.

188. Talonid, wear pattern within the talonid basin, present:

(0) Absent;

(1) Present.

Microtherulum = 1

This character is applicable to those taxa with triangulated molars.

189. Talonid, wear pattern within the talonid basin, apical wear:

(0) Not on the crests or lophodont;

(1) On the crests of the talonid;

(2) On the crest and lophodont.

Microtherulum = 0

This character is applicable to those taxa with triangulated molars.

190. Distal metacristid, presence:

(0) Present;

(1) Absent.

Microtherulum = 1

This character is applicable only to taxa with reversed triangulation.

191. Wear facets 5 and 6, labial face of the entoconid, differentiation, presence:

(0) Absent;

(1) Present.

Microtherulum = 1

192. Talonid, occluding surfaces, morphology:

(0) Smooth surface on the talonid heel (or on cusp d);

(1) Multiple ridges within the talonid basin;

(2) Talonid present, but wear occurs apically on the crests of cristid obliqua and hypoconid cristid (V-shaped talonid crests).

Microtherulum = 0

This character is only applicable to taxa with reversed triangulation.

193. Molars, wear facets pseudo-3 and pseudo-4, presence:

(0) Absent;

(1) Present.

Microtherulum = 0

194. Molars, wear facets pseudo-5 and pseudo-6:

(0) Absent;

(1) Present.

Microtherulum = 0

195. Lower molars, pseudo cusp e and f hypertrophied:

(0) Absent;

(1) Present.

Microtherulum = 0

Other Dental Features:

196. Upper incisors, number:

(0) Five;

(1) Four;

(2) Three;

(3) Two;

(4) One;

(5) No incisors.

Microtherulum = 0

197. I2, size, enlargement:

(0) Absent;

(1) Present.

Microtherulum = 0

198. Posterior upper incisors, cusp number:

(0) One;

(1) Two or more.

Microtherulum = 0

199. Lower incisors, number:

(0) Five or more;

(1) Four;

(2) Three;

(3) Two;

(4) One;

(5) No incisors.

Microtherulum = 1

200. Lower incisor, anterior-most incisor, enamel structure:

(0) Covers the whole incisor;

(1) Restricted anteriorly.

Microtherulum = 0

201. Lower incisor, anterior-most incisor, open root, presence:

(0) Absent;

(1) Present.

Microtherulum = 0

202. Upper incisor, anterior-most incisor, enamel structure:

(0) Covers the whole incisor;

(1) Restricted anteriorly.

Microtherulum = 0

203. Upper anterior-most incisor, open root, presence:

(0) Absent;

(1) Present.

Microtherulum = 0

204. Upper canine, presence:

(0) Present;

(1) Absent

Microtherulum = 0

205. Upper canine, size:

(0) Enlarged;

(1) Small;

Microtherulum = 0

206. Upper canine, cusp number:

(0) Peg-like with single cusp;

(1) Two or more cusps.

Microtherulum = 0

207. Upper canine, root number:

(0) One;

(1) Two.

Microtherulum = 0

208. Lower canine, presence:

(0) Present;

(1) Absent.

Microtherulum = 0

209. Lower canine, size:

(0) Enlarged;

(1) Small.

Microtherulum = 0

210. Lower canine, root number:

(0) One;

(1) Two.

Microtherulum = 0

211. Upper premolars, number:

(0) Five or more;

(1) Four;

(2) Three;

(3) Two or less.

Microtherulum = 0

This character is only applicable to taxa with premolar vs. molar differentiation.

212. Lower premolars, number:

(0) Five or more;

(1) Four;

(2) Three;

(3) Two or less.

Microtherulum = 0

213. Lower molars or molariform postcanines, number:

(0) Six or more;

(1) Five;

(2) Four;

(3) Three;

(4) Two or less.

Microtherulum = 3

This character is applicable only to those taxa that do not have multiple dental replacements.

214. Upper molars or molariform postcanines, number:

(0) Six or more;

(1) Five;

(2) Four;

(3) Three;

(4) Two or less.

Microtherulum = 3

This character is applicable only to those taxa that do not have multiple dental replacements.

215. Upper postcanine loci, number:

(0) More than 8 (including the loci plus the alveoli of shed anterior postcanines);

(1) Eight;

(2) Seven,

(3) Six;

(4) Five or less.

Microtherulum = 1

216. Lower postcanine loci, number:

(0) Eight or more;

(1) Seven;

(2) Six;

(3) Five or fewer.

Microtherulum = 0

217. First (functional) upper premolar or postcanine, procumbency in relation to the upper canine, presence:

(0) Absent;

(1) Present.

Microtherulum = 0

218. Diastema between first (functional) upper premolar/postcanine and upper canine, presence:

(0) Absent;

(1) Present.

Microtherulum = 0

219. Diastema separating the lower first and second premolars (defined as the first and second functioning premolar or premolariform postcanine), length:

(0) Absent or short (gap less than one tooth root for whichever is smaller of the adjacent teeth);

(1) Present, subequal to one tooth-root diameter or more;

(2) Present, equal to or more than one-tooth length.

Microtherulum = 0

220. Ultimate lower premolar, bladed or crenulated, presence:

(0) Absent;

(1) Present.

Microtherulum = 0

221. Upper anterior-most incisor, morphology:

(0) Subequal to the remaining incisors, no diastema with the second incisor;

(1) Anteriorly projecting, separated from the second incisor (or any following teeth if posterior incisors are absent) by a diastema;

(2) Absent (as evidenced by a median gap between the mesial-most incisors).

Microtherulum = 0

222. Ultimate and penultimate upper incisors, morphology:

(0) Peg-like;

(1) Spoon-shaped to rhomboid-shaped in lateral view;

(2) Spatulate in lateral view;

(3) Ultimate and/or penultimate upper incisors bicuspate or tricuspate.

Microtherulum = 0

223. Lower incisor, staggered, presence:

(0) Absent;

(1) Present.

Microtherulum = 0

224. Incisors and canines, replacement pattern:

(0) More than one replacement;

(1) One replacement;

(2) No replacement.

Microtherulum = ?

225. Molariform postcanines, replacement, at some posterior functional molariform postcanine loci, presence:

(0) Present;

(1) Absent.

Microtherulum = 1

226. Lower anterior-most incisor, procumbency, presence:

(0) Absent;

(1) Present (at least 50% longer than the adjacent incisor).

Microtherulum = 0

227. Lower anterior-most incisor, size, enlargement:

(0) Absent;

(1) Present (at least 50% longer than the adjacent incisor).

Microtherulum = 0

228. Lower incisor-canine region, enlarged diastema (better developed in older individuals):

(0) Absent;

(1) Present and behind the canine;

(2) Present and behind the posterior incisor.

Microtherulum = 0

229. Lower multi-rowed molars, U-shaped transverse ridge, presence:

(0) Absent;

(1) Present.

Microtherulum = NA

230. Lower multi-rowed molars, U-shaped transverse ridge,position:

(0) At second anterior cusp;

(1) At the anterior rim .

Microtherulum = NA

231. Anterior upper molars, fusuliform (“spindle-shaped”) shearing valley, presence:

(0) Absent;

(1) Present.

Microtherulum = NA

232. Multi-rowed lower molars, lingual row, cusp ratio:

(0) Cusps are of subequal height;

(1) Mesial cusp on the lingual row the highest;

(2) Mesial cusp on the lateral row the highest. (new state)

Microtherulum = NA

233. Multi-rowed lower molars, buccal row, cusp ratio:

(0) All cusps are of equal height;

(1) The middle cusps higher than the mesial and distal cusps;

(2) The mesial cusps higher than distal cusps. (new state)

Microtherulum = NA

234. Lower m1, second cusp of lingual row, enlarged and more centrally placed:

(0) Absent;

(1) Present

Microtherulum = NA

This character is applicable only to molars with multi-rows of multiple cusps.

235. Utimate upper molar, labial row of multicusp rows, cusp ratio:

(0) Distal cusp highest, with a gradient of anteriorly decreasing height;

(1) Cusps in same row of equal height.

(2) Mesial cusp is slightly higher than distal cusp

Microtherulum = NA

236. Last (ultimate) upper molar, alignment of multi-cusped rows, lingual offset from the penultimate molar, presence :

(0) Absence;

(1) Presence.

Microtherulum = NA

237. Lower m2, complete middle valley between lingual cusp row and labial cusp row, presence:

(0) Absent;

(1) Present.

Microtherulum = NA

238. Multi-rowed ultimate lower molar, row length difference:

(0) Labial cusp row about equal as lingual cusp row;

(1) Labial row is shorter at the anterior end (by at least halfcusp length) than lingual row;

(2) Labial row is longer at the posterior end than lingual row (by at least half-cusp length).

Microtherulum = NA

239. Enamel microstructure:

(0) Synapsida columnar enamel (prismless);

(1) ‘Transitional’ (sheath indistinct, ‘prismatic’ crystallites inclined at less than 45o to the ‘interprismatic’ matrix);

(2) Full prismatic enamel;

(3) Enamel absent.

Microtherulum = ?

240. Postcanines, hypsodonty root, presence:

(0) Absent; (1) Present

Microtherulum = 0

241. Postcanines, open root, presence:

(0) Absent; (1) Present.

Microtherulum = 0

242. Postcanines, root, division, morphology:

(0) Single root;

(1) Divided roots connected by dentine sheets;

(2) Two or three complete divided roots;

(3) Multiple roots coalesced;

(4) More than three roots.

Microtherulum = 2

Vertebrae and Ribs:

243. Alas, fuion of the atlas neural arch and intercentrum, presence:

(0) Absent;

(1) Present.

Microtherulum = ?

244. Atlas rib, presence:

(0) Present;

(1) Absent.

Microtherulum = 1

245. Axis, fusion of dens to the axis, presence:

(0) Absent;

(1) Present.

Microtherulum = ?

246. Axis, axis rib, presence:

(0) Present;

(1) Absent (rib fused to form the transverse process).

Microtherulum = ?

247. Cervical vertebrae, postaxial cervical ribs:

(0) Unfused;

(1) Fused.

Microtherulum = ?

248. Vertebrae bearing ribs, number:

(0) 13 or less;

(1) 15 or more.

Microtherulum = 0

249. Overlapping ventral costal plates, presence:

(0) Absent;

(1) Present.

Microtherulum = ?

250. Overlapping lumbar or posterior thoracic ribs, presence:

(0) Present;

(1) Absent.

Microtherulum = 0

251. Anticlinal vertebra, presence:

(0) Absent;

(1) Present.

Microtherulum = ?

252. Anticlinal vertebra, position:

(0) Posteriorly position (within last 4 lumbar vertebrae) (*Megaconus, Monotremes*, *Yanoconodon*, *Repenomamus*, *Gobiconodon*);

(1) Anteriorly positioned (within the anterior 13 dorsal and the thoracic vertebral region if thoraco-lumbar boundary is distinctive) (*Jeholodens*).

Microtherulum = ?

253. Mobile lumbar ribs, presence:

(0) Present;

(1) Absent.

Microtherulum = ?

254. Lumbar ribs or transverse processes, orientation:

(0) Posterolaterally directed;

(1) Laterally or anterolaterally directed.

Microtherulum = 1

255. Xenarthrous articulation in addition to the pre- and post-zygapophyses of lumbar vertebrae, presence:

(0) Absent; (1) Present.

Microtherulum = 0

Shoulder Girdle:

256. Interclavicle, presence:

(0) Present; (1) Absent.

Microtherulum = 0

257. Interclavicle, contact relationships between the interclavicle (embryonic membranous element) and the sternal manubrium (embryonic endochondral element) (assuming the homologies of these elements by Klima, 1973, 1987):

(0) Two elements distinct from each other, posterior end of the interclavicle abuts with the anterior border of manubrium;

(1) Two elements distinct from each other, the interclavicle broadly overlaps the ventral side of the manubrium;

(2) Complete fusion of the embryonic membranous and endochondral elements resulting in a single and enlarged manubrium.

Microtherulum = ?

258. Interclavicle, distal expansion, presence:

(0) Absent; (1) Present

Microtherulum = ?

259. Interclavicle/manubrium, cranial margin:

(0) Emarginated or flat;

(1) With a median process.

Microtherulum = ?

260. Interclavicle to sternal manubrium, length ratio:

(0) Interclavicle twice the length of manubrium;

(1) Interclavicle nearly equal to manubrium in length.

Microtherulum = ?

261. Sternoclaviclular joint (assuming that homologous elements of the interclavicle and the manubrium are fused to each other in therians, Klima, 1973, 1987):

(0) Immobile; (1) Mobile.

Microtherulum = ?

262. Interclavico-manubrial craniolateral process, presence:

(0) Absent; (1) Present.

Microtherulum = ?

263. Acromioclavicular joint, articular degree:

(0) Extensive articulation;

(1) Limited articulation (either pointed acromion, pointed distal end of clavicle, or both).

Microtherulum = ?

264. Clavicle, curvature:

(0) Boomerang-shaped; (1) Slightly curved.

Microtherulum = ?

265. Scapula, supraspinous fossa along the length, development:

(0) Present only in the "acromional region" of the scapula, and on the cranial (dorsal) border of the scapula and positioned anterior to the glenoid);

(1) Weakly developed (present only along a part of the scapula and positioned lateral to the glenoid);

(2) Fully developed (present along the entire dorsal border of the scapula).

Microtherulum = ?

266. Scapula, proportion of supraspinous vs. infraspinous fossae (width measured across the "saddle region" of the spine, or near the mid-length of the scapula):

(0) Supraspinous "fossa" on the cranial aspect of the scapula and much narrower than infraspinous fossa;

(1) Supraspinous width is 50% to 80% that of infraspinous fossa;

(2) Fossae subequal;

(3) Supraspinous over 150% that of infraspinous fossa.

Microtherulum = ?

267. Scapula, acromion process, shape:

(0) Short stump, level with or behind the glenoid;

(1) Hook-like and extending below the glenoid.

Microtherulum = ?

268. Scapula, a distinctive fossa for the teres major muscle on the lateral aspect of the scapular plate, presence:

(0) Absent; (1) Present.

Microtherulum = ?

269. Procoracoid, fused to the sternal apparatus (Klima 1973), presence:

(0) Absent; (1) Present.

Microtherulum = ?

270. Procoracoid foramen, presence:

(0) Present;

(1) Absent (assuming the procoracoid is fused to the sternal apparatus in living therians, Klima, 1973).

Microtherulum = ?

271. Coracoid, size:

(0) Large, with posterior process;

(1) Small, without posterior process.

Microtherulum = ?

272. Coracoid, anterior process of the coracoid, development:

(0) Indistinctive;

(1) Distinctive;

(2) Distinctive and forming a broad plate.

Microtherulum = ?

273. Coracoid process bridging over posteriorly toward the vertebral border of scapula (or fused with the latter), presence:

(0) Absent; (1) Present.

Microtherulum = ?

274. Anterior-most element (‘manubrium’) relative to the subsequent sternebrae in the sternal apparatus, size:

(0) Large; (1) Small.

Microtherulum = ?

275. Glenoid, orientation (‘facing’ of the articular surface), relative to the plane or the long axis of the scapula):

(0) Nearly parallel and facing posterolaterally;

(1) Oblique and facing more posteriorly;

(2) Perpendicular.

Microtherulum = ?

276. Glenoid, shape:

(0) Saddle-shaped, oval;

(1) Uniformly concave and more rounded in outline.

Microtherulum = ?

277. Glenoid, curvature:

(0) Elongate;

(1) Rounded in outline.

Microtherulum = ?

278. Scapula, medial surface, shape:

(0) Convex; (1) Flat.

Microtherulum = ?

279. Suprascapular incisure (defined as the prominent emargination on the cranial border of the supraspinous fossa), presence:

(0) Absent; (1) Present.

Microtherulum = ?

Forelimb and Manus

280. Humerus, humeral head, shape:

(0) Subspherical, weakly inflected;

(1) Spherical, strongly inflected.

Microtherulum = ?

281. Humerus, intertubercular groove of the humerus, outline:

(0) Shallow and broad;

(1) Narrow and deep.

Microtherulum = ?

282. Humerus, lesser tubercle, size relative to the greater tubercle:

(0) Wider;

(1) Narrower.

Microtherulum = ?

283. Humerus, torsion between the proximal and distal ends, degree:

(0) Strong (≥30°);

(1) Moderate (30°–15°);

(2) Weak.

Microtherulum = ?

284. Humerus, ventral extension of the deltopectoral crest or the position of the deltoid tuberosity, development:

(0) Short and limited to the proximal part of the humeral shaft;

(1) Extending ventrally (distally) at least 1/3 the length of the shaft.

Microtherulum = ?

285. Humerus, teres tuberosity on medial side of humerus, presence:

(0) Absent; (1) Present;

(2) Hypertrophied.

Microtherulum = ?

286. Humerus, teres tuberosity on medial side of humerus, hypertrophied:

(0) Absent; (1) Present;

Microtherulum = ?

287. Ulna, articulation on the distal humerus, morphology:

(0) Bulbous ulnar condyle;

(1) Cylindrical trochlea in posterior view with a vestigial ulnar condyle in anterior view;

(2) Cylindrical trochlea without an ulnar condyle (cylindrical trochlea extending to the anterior/ventral side).

Microtherulum = ?

288. Radius, radial articulation on the distal humerus, morphology:

(0) Distinct and rounded radial condyle in both anterior (ventral) and posterior (dorsal) aspects (that does not form a continuous synovial surface with the ulnar articulation in the ventral/anterior view of the humerus);

(1) Rounded radial condyle anteriorly but cylindrical posteriorly;

(2) Capitulum (forming a continuous synovial surface with the ulnar trochlea; cylindrical in both anterior and posterior aspects).

Microtherulum = ?

289. Humerus, entepicondyle and ectepicondyle, development:

(0) Robust; (1) Weak.

Microtherulum = ?

290. Humerus, sigmoidal shelf for the supinator ridge extending proximally from the Ectepicondyle, presence:

(0) Absent; (1) Present.

Microtherulum = ?

291. Ulna, coronoid process of semilunar notch of ulna, presence:

(0) Absent;

(1) Present.

Microtherulum = ?

292. Ulna, coronoid process of semilunar notch of ulna, height:

(0) At the level to olecranon process;

(1) Higher than olecranon process;

Microtherulum = ?

293. Radius, styloid process of the radius:

(0) Weak; (1) Strong.

Microtherulum = ?

294. Scaphoid, enlargement:

(0) Not enlarged (scaphoid ≤150% of the lunate);

(1) Enlarged (scaphoid twice the size of the lunate);

(2) Enlarged with a distolateral process.

Microtherulum = ?

295. Hamate (unciform), size:

(0) About equal size to the triquetrum;

(1) Hypertrophied, much larger than the triquetrum.

Microtherulum = ?

296. Hamate (unciform), shape:

(0) Anteroposteriorly compressed;

(1) Mediolaterally compressed.

Microtherulum = ?

297. Trapezium, morphology:

(0) Elongate to cuboidal, larger than or subequal to the trapezoid;

(1) Bean-shaped or fusiform, smaller than the trapezoid.

Microtherulum = ?

298. Triquetrum-lunate, proportion:

(0) Triquetrum nearly twice the size of the lunate;

(1) Triquetrum subequal to the lunate.

Microtherulum = ?

299. Metacarpals, relative length to proximal phalanx of digit III:

(0) PP (proximal phalanx) shorter than MC (metacarpal);

(1) PP longer than MC.

Microtherulum = ?

Pelvic Girdle:

300. Ilium, anterior process, length:

(0) Short (less than the diameter of the acetabulum);

(1) Long, 1-1.5 times the diameter of the acetabulum;

(2) Elongate, more than 1.5 times the diameter of the acetabulum.

Microtherulum = 2

301. Ilium, posterior process, presence:

(0) Present;

(1) Reduced or absent.

Microtherulum = 1

302. Acetabulum, acetabular dorsal emargination:

(0) Open (emarginated);

(1) Closed (with a complete rim).

Microtherulum = 1

303. Acetabulum, sutures of the ilium, ischium, and pubis within the acetabulum, presence:

(0) Present;

(1) Fused.

Microtherulum = ?

304. Ischium, ischiatic dorsal margin and tuberosity:

(0) Dorsal margin concave (emarginated) and ischiatic tuberosity present;

(1) Dorsal margin concave and ischiatic tuberosity hypertrophied;

(2) Dorsal margin straight and ischiatic tuberosity small.

Microtherulum = 2

305. Ischium, posterior spine, morphology:

(0) Short and pointed;

(1) Expanded with oblique posterior spine;

(2) Expanded and truncated.

Microtherulum = 2

306. Epipubic bone, presence:

(0) Present; (1) Absent.

Microtherulum = 0

307. Epipubic bone, width:

(0) Narrow; (1) wide.

Microtherulum = 0

308. Sacral vertebrae, fusion with the proximal caudal vertebrae, presence:

(0) Absent; (1) Present.

Microtherulum = 0

309. Ischium, fusion with the caudal vertebrae, presence:

(0) Absent; (1) Present.

Microtherulum = 0

310. Ilium, preacetabular tubercle for M. rectus femoris, presence:

(0) Absent; (1) Present.

Microtherulum = 0

311. Acetabulum, fully encircled synovial surface, presence:

(0) Absent; (1) Present

Microtherulum = 0

312. Pubis, lesser psoas tuberosity or process, presence:

(0) Absent; (1) Present.

Microtherulum = ?

Hind limb and Pes:

313. Femur, inflected head of the femur set off from the shaft by a neck:

(0) Neck absent and head oriented dorsally;

(1) Neck present, head spherical and inflected medially.

Microtherulum = 1

314. Femur, femoral head, fovea for the acetabular ligament, presence:

(0) Absent; (1) Present.

Microtherulum = ?

315. Femur, greater trochanter, orientation:

(0) Directed dorsolaterally;

(1) Directed dorsally.

Microtherulum = 1

316. Femur, level of greater trochanter relative to the femoral head:

(0) Mid-level of femoral head;

(1) Top level of femoral head

Microtherulum = 0

317. Femur, lesser trochanter, position:

(0) On medial side of the shaft;

(1) On the ventromedial or ventral side of the shaft.

Microtherulum = ?

318. Femur, lesser trochanter, size:

(0) Large;

(1) Small to absent.

Microtherulum = ?

319. Femur, third trochanter, presence:

(0) Absent;

(1) Present.

Microtherulum = 0

320. Femur, third trochanter, as a continuous ridge connected to the greater trochanter:

(0) Absent;

(1) Present;

(2) Present as a continuous ridge connected to the greater trochanter.

Microtherulum = NA

321. Femur, patellar facet (‘groove’), development:

(0) Absent;

(1) Shallow and weakly developed;

(2) Well-developed.

Microtherulum = ?

322. Tibia, proximo-lateral tubercle or tuberosity, mrophology:

(0) Large and hook-like;

(1) Indistinct;

(2) Fused to fibula

Microtherulum = 1

323. Tibia, distal end, tibial malleolus (medial malleolus), development:

(0) Weak;

(1) Distinctive.

Microtherulum = 0

324. Tibia, lateral tibio-astragalar condyle and medial tibio-astragalar condyle, differentiation, presence:

(0) Absent;

(1) Present.

Microtherulum = 1

325. Fibula, contacting the distal end of the femur, presence:

(0) Present;

(1) Absent;

(2) Fibula contacting through fusion with the tibia.

Microtherulum = 0

326. Tibia and fibula, distal portions, fused:

(0) Absent; (1) Present.

Microtherulum = 0

327. Fibula, enlarged parafibular structure, presence:

(0) Absent; (1) Present

Microtherulum = 0

328. Parafibula types:

(0) Separate bone and unfused to the fibular;

(1) Fused to fibula as an enlarged process:

Microtherulum = ?

329. Fibula, distal end, fubular malleolus (lateral malleolus), development:

(0) Weak or absent;

(1) Distinct.

Microtherulum = 0

330. Fibula, contacting the calcaneus (= 'tricontact in upper ankle joint' of Szalay, 1994), articulation:

(0) Extensive contact;

(1) Reduced;

(2) Absent.

Microtherulum = 1

331. Astragalus, superposition (overlap) over the calcaneus (lower ankle joint), degree:

(0) Little or absent;

(1) Weakly developed;

(2) Present.

Microtherulum = ?

332. Astragalo-navicular articulation, symmetry to the neck:

(0) Articulating facet indistinctive;

(1) Asymmetrical: present only on the lateral side of the "neck region", or Szalay's [1994] comment on "necklessness");

(2) Symmetrical with regard to the astragalar neck.

Microtherulum = ?

333. Astragalar neck, basal width (justification for separating this character from the navicular facet expansion is that the latter concerns symmetry, whereas this character deals with proportion; the distributions of these two character are different in some stem eutherians and crown

marsupials):

(0) Neck narrower than the head (constriction posterior to navicular facet);

(1) Neck about same width as the head (with parallel sides posterior to navicular facet);

(2) Widest point of neck at mid-length (widening is not developed near the base of the neck);

(3) Astragalar neck widest at the base.

Microtherulum = ?

334. Astragalo-navicular contact, ratio:

(0) Navicular contact transversely wider than dorsoventrally thick;

(1) Navicular contact dorsoventrally thicker than transversely wide.

Microtherulum = ?

335. Navicular contact, expansion in the astragalar head region, degree:

(0) Restricted anteriorly;

(1) Asymmetrical spread only to the medial side of the astragalar "head-neck region";

(2) Symmetrical spread of the navicular facet to both the lateral and the medial sides of the neck (symmetrical with regards to the main axis of the neck).

Microtherulum = ?

336. Astragalo-navicular contact, shape:

(0) Flat to convex;

(1) Crest-in-groove: transverse groove on astragalar head to receive crest from navicular.

Microtherulum = ?

337. Astragalar trochlea (defined as a saddle-shaped upper ankle joint), presence:

(0) Absent;

(1) Present.

Microtherulum = ?

338. Astragalar trochlea (defined as a saddle-shaped upper ankle joint), development:

(0) Weak (defining crest on the medial astragalo-tibial facet weakly developed);

(1) Distinct with clear separation of the medial and lateral tibial facets.

Microtherulum = ?

339. Astragalus, well-defined medio-tibial crest (more or less parallel to the tibio-fibular crest), presence:

(0) Absent; (1) Present.

Microtherulum = ?

340. Astragalus, astragalar medial plantar tuberosity, development:

(0) Absent;

(1) Weakly developed;

(2) Ventrally flaring or protruding.

Microtherulum = ?

341. Calcaneus, calcaneal tubercle, distal end, shape:

(0) Short, dorso-ventrally compressed, without a terminal swelling;

(1) Dorso-ventrally compressed, with a terminal swelling;

(2) Elongate, vertically deep, and mediolaterally compressed, with terminal swelling.

Microtherulum = 2

342. Calcaneus, peroneal process, morphology:

(0) Laterally expanded shelf, larger than the combined length of the sustentacular and astragalar facets, lateral to the astragalar facet;

(1) With a distinct and long peroneal process, laterally projecting;

(2) With a distinct peroneal process, demarcated by a deep peroneal groove at the base;

(3) Laterally directed, small peroneal shelf demarcated from the anterior (cuboidal) edge of the calcaneus;

(4) Anterolaterally directed, hypertrophied peroneal process/shelf;

(5) Peroneal structure laterally reduced (lateral surface is straight from the calcaneal tubercle).

Microtherulum = ?

343. Calcaneus, base of the peroneal process, placement relative to the level of the cuboid facet of the calcaneus:

(0) Peroneal structure posterior to the level of the cuboid facet;

(1) Peroneal structure developed anteriorly at the same level as the cuboid facet;

(2) Peroneal structure hypertrophied, extending anteriorly beyond the level of the cuboid facet.

Microtherulum = ?

344. Calcaneus, peroneal groove, development:

(0) Indistinct, on the anterolateral aspect of the lateral shelf;

(1) Distinct, deep separation of the peroneal process;

(2) Weakly developed, with shallow groove on the lateral side of the process; (3) Distinct, on the anterolateral corner of the peroneal process.

Microtherulum = ?

345. Cuboid, alignment to the main axis of the calcaneus (horizontal plane):

(0) On the anterior (distal) end of the calcaneus (the cuboid is aligned with the long axis of the calcaneus);

(1) On the anteromedial aspect of the calcaneus (the cuboid is skewed to the medial side of the long axis of the calcaneus):

Microtherulum = ?

346. Calcaneocuboid joint, orientation in dorso-ventral plane:

(0) Calcaneocuboid facet on the calcaneus oriented ventrally (more visible in the plantar view than in dorsal view);

(1) Calcaneocuboid facet oriented anteriorly (distally);

(2) Calcaneocuboid facet oriented ventromedially or medio-obliquely.

Microtherulum = ?

347. Calcaneocuboid joint, articular facet, shape:

(0) Calcaneocuboid facet on the calcaneus relatively flat to slightly concave;

(1) Saddle-shaped (differentiation of dorsal vs. proximal calcaneocuboid “facets” so that the whole calcaneocuboid joint is saddle shaped).

Microtherulum = ?

348. Lower ankle joint, sustentacular facet of the calcaneus, orientation, in relation to the horizontal plane:

(0) Nearly vertical;

(1) Oblique (≤70°) to nearly horizontal.

Microtherulum = ?

349. Calcaneus, sustentacular facet, antero-posterior placement relative to the astragalar facet:

(0) Directly anterior to the astragalar facet and vertically oriented on the medial edge of the calcaneus;

(1) On the dorsal aspect and positioned anteromedial to the astragalar facet on the calcaneus;

(2) On the dorsal aspect, medial to the astragalar facet;

(3) On the dorsal aspect, anterior to the astragalar facet.

Microtherulum = ?

350. Calcaneus, confluence of the sustentacular facet and the astragalar facet, presence:

(0) Absent; (1) Present.

Microtherulum = ?

351. Calcaneus, sustentacular process, ventral outline:

(0) Indistinctive;

(1) Medially directed shelf, with rounded outline;

(2) Protruding triangle, posteromedially directed;

Microtherulum = ?

352. Sustentacular facet/process (using the most salient point of the facet/process in ventral view as landmark), antero-posterior position relative to the length of the calcaneus:

(0) Near the mid-point;

(1) Near the anterior (proximal) one-third.

Microtherulum = ?

353. Calcaneus, posterior calcaneo-astragalar process/protuberance and its contiguous fibular contact (if the fibula contact is present in medial view), shape:

(0) Indistinctive (boundary not defined and confluent with fibular contact);

(1) Well defined, and oblong to ellipsoidal;

(2) Nearly spherical and bulbous, more transversely developed than character state 1;

(3) Transversely confluent with the sustentacular facet.

Microtherulum = ?

354. CAF structure (structure of the calcaneoastragalar contact), placement:
(0) On the medial side of the body of the calcaneus;
(1) On the dorsal side of the body of the calcaneus, but bordering on the body's medial margin (without a protruding outline);
(2) On the dorsal side of the body of the calcaneus and protruding beyond the body's medial margin;
(3) Withdrawn and separated from the medial margin and placed along the lateral margin of the body of the calcaneus.
Microtherulum = ?

355. Calcaneus, anterior ventral (plantar) tubercle, presence:
(0) Absent;
(1) Present, at the anterior edge (just lateral to the cuboid facet);
(2) Present, set back from the anterior edge.
Microtherulum = ?

356. Calcaneus, anteroventral groove or depression, presence:
(0) Absent; (1) Present.
Microtherulum = ?

357. Calcaneus, cross-sectional shape at the level of the posterior calcaneoastragalar facet:
(0) Dorso-ventrally compressed;
(1) Mediolaterally compressed.
Microtherulum = ?

358. Calcaneal tubercle, ventral curvature, presence:
(0) Present; (1) Absent.
Microtherulum = ?

359. Navicular and cuboid, proportion (transverse width measured in dorsal view):
(0) Navicular narrower than or subequal to cuboid;

(1) Navicular wider than cuboid.

Microtherulum = 1

360. Entocuneiform, mesocuneiform, and ectocuneiform, proportion (in ventral view):

(0) Mesocuneiform and ectocuneiform small, their combined width smaller than the width of the entocuneiform;

(1) Mesocuneiform and ectocuneiform large, their combined width (in dorsal view) exceeding the width of the entocuneiform.

Microtherulum =0

361. Entocuneiform and proximal end of metatarsal 1, saddle-shaped contact, presence: (0) Absent; (1) Present.

Microtherulum = 0

362. Cuboid, medio-plantar aspect, deeply notched by the peroneus longus tendon, presence:

(0) Absent; (1) Present.

Microtherulum = 0

363. Prehallux, presence:

(0) Absent; (1) Present.

Microtherulum = 0

364. Metatarsal V and the peroneal process of the calcaneus, end-to-end contact, presence:

(0) Absent; (1) Present.

Microtherulum = 0

365. Metatarsal V, relationships of the proximal end to the cuboid:

(0) Metatarsal V is off-set from the lateral side of the cuboid;

(1) Metatarsal V is so far off-set to the side of the cuboid that it contacts the calcaneus;

(2) Metatarsal V is level with (not offset from) the anterior end of the cuboid.

Microtherulum = 2

366. Metatarsal V, ventrolateral tubercle at the proximal end, development:

(0) Absent or indistinctive;

(1) Present, at or anterior to the anterior edge of the calcaneus;

(2) Present, off-set posteriorly from the anterior edge of the calcaneus.

Microtherulum = 1

367. Metatarsal III, angle to the calcaneus (which indicates how much the sole of the foot is 'bent' from the long axis of the ankle):

(0) Metatarsal III aligned with (or parallel to) the long axis of the calcaneus;

(1) Metatarsal III arranged obliquely from the long axis of the calcaneus.

Microtherulum = ?

368. Metatarsal II and metatarsal III, proximal ends, placement:

(0) II and III even or II more proximal than III;

(1) III more proximal than II.

Microtherulum = 0

369. Opposable hallux, presence:

(0) Absent; (1) Present.

Microtherulum = 0

370. Metatarsals and proximal phalanx of digit III, length:

(0) PP (proximal phalanx) shorter than MT (metatarsal); (1) PP longer than MT.

Microtherulum = 0

Other Postcranial Characters:

371. Ossified patella, presence:

(0) Absent; (1) Present.

Microtherulum = 1

372. Sesamoid bones in the digital flexor tendons:

(0) Absent;

(1) Present.

Microtherulum = 1

373. Sesamoid bones in the digital flexor tendons, paired:

(0) Absent;

(1) Present.

Microtherulum = ?

374. External pedal (tarsal) spur, presence:

(0) Absent; (1) Present.

Microtherulum = 0

375. Pes digital grouping:

(0) Didactylous; (1) Syndactylous.

Microtherulum = 0

376. Epiphyses in long bones, presence:

(0) Absent; (1) Present.

Microtherulum = 1

Basicranium:

377. Squamosal, cranial moiety, external size:

(0) Narrow;

(1) Broad;

(2) Expanded posteriorly to form the skull roof table.

Microtherulum = ?

378. Cranial moiety of the squamosal in the endocranial wall of the braincase, participation, presence:

(0) Absent; (1) Present.

Microtherulum = ?

379. Squamosal and parietal, multiple vascular foramina (for rami temporales), presence:

(0) Absent; (1) Present.

Microtherulum = ?

380. Frontal, dorsal surface, multiple vascular foramina (for branches of external ethmoidal artery), presence:

(0) Absent; (1) Present.

Microtherulum = ?

381. Dentary-squamosal contact (or glenoid) and the cranial moiety of the squamosal, topographic relationships:

(0) Contact on the internal aspect of the zygoma, without a constricted neck;

(1) Contact on the zygoma, with a constricted neck;

(2) Contact on the cranial moiety of squama;

(3) Contact on zygoma, without a constricted neck.

Microtherulum = 2

This character is only applicable to taxa with the dentary-squamosal joint; this character is best seen in ventral view.

382. Squamosal, anterior to its zygomatic root, cross-section profile:

(0) Rounded or triangular and tapering anteriorly;

(1) Dorsoventral expanded and mediolaterally compressed, and not tapering anteriorly.

Microtherulum = ?

383. Squamosal, postglenoid depression, development:

(0) Present as the post-craniomandibular joint sulcus ("external auditory meatus" on the zygoma);

(1) Absent;

(2) Present on the skull base.

Microtherulum = 2

384. Squamosal, entoglenoid process, development:

(0) Absent or vestigial;

(1) Present, but separated from the postglenoid process;

(2) Present, enlarged and connected to the postglenoid process.

Microtherulum = ?

385. Craniomandibular joint, position:

(0) Posterior or lateral to the level of the fenestra vestibuli;

(1) Anterior to the level of the fenestra vestibuli.

Microtherulum = ?

386. Squamosal, glenoid, orientation:

(0) On the inner side of the zygoma and facing ventromedially;

(1) On the platform of the zygoma and facing ventrally.

Microtherulum = 1

387. Squamosal, postglenoid process, development:

(0) Absent;

(1) Postglenoid crest raised below the fossa, but without a distinctive process;

(2) Distinctive process;

(3) Distinctive process buttressed by ectotympanic.

Microtherulum = 2

388. Postglenoid foramen, position:

(0) Posterior to the glenoid area;

(1) Medial to the postglenoid process;

(2) Anterior to the postglenoid process.

Microtherulum = ?

389. Postglenoid foramen, presence:

(0) Absent;

(1) Present.

Microtherulum = ?

390. Postglenoid foramen, composition:

(0) In the squamosal;

(1) Between the squamosal and petrosal;

(2) Between the squamosal and ectotympanic.

Microtherulum = ?

391. Glenoid fossa, medial margin, composition:

(0) Formed by the squamosal;

(1) Formed by the alisphenoid.

Microtherulum = ?

392. Squamosal, epitympanic recess, composition :

(0) No contribution to the "epitympanic area" of the petrosal;

(1) Small contribution to the posterolateral wall of the epitympanic recess;

(2) Large contribution to the lateral wall of the epitympanic recess;

(3) Squamosal forming a large part of enlarged epitympanic sinus.

Microtherulum = ?

393. External bony housing of the cochlea, contribution of the basisphenoid wing (parasphenoid ala):

(0) Participates in the rim of the fenestra vestibuli;

(1) Does not reach the rim of the fenestra vestibuli;

(2) Absent or excluded from the cochlear housing.

Microtherulum = ?

394. Cochlear housing, relationship to the lateral lappet of the basioccipital:

(0) Entirely covered by the basioccipital;

(1) Medial aspect covered by the basioccipital;

(2) Partially (~about half width on the medial side) covered by the basioccipital;

(3) Fully exposed as the promontorium.

Microtherulum = 3

395. Fenestra vestibule, thickened rim, presence:

(0) Present; (1) Absent.

Microtherulum = 1

396. Cochlear housing, fully formed by the petrosal, presence:

(0) Absent; (1) Present.

Microtherulum = 1

397. Ppromontorium, ventromedial surface, shape:

(0) Flat; (1) Inflated and convex.

Microtherulum = 1

398. Promontorium, lateral wall and overall external outline:

(0) Triangular, with a steep and slightly concave lateral wall;

(1) Elongate and cylindrical;

(2) Bulbous and oval haped.

Microtherulum = 2

399. Cochlea, morphology:

(0) Cochlear recess (without a canal);

(1) Short canal;

(2) Elongate canal, to the fullest extent of the promontorium;

(3) Slightly curved;

(4) Elongate and partly coiled;

(5) Elongate and coiled to at least 360°.

Microtherulum = ?

400. Internal acoustic meatus, cribriform plate, presence:

(0) Absent; (1) Present.

Microtherulum = ?

401. Internal acoustic meatus, depth:

(0) Deep with thick prefacial commissure;

(1) Shallow with thin prefacial commissure.

Microtherulum = ?

402. Cochlear canal, primary bony lamina, presence:

(0) Absent; (1) Present.

Microtherulum = ?

403. Cochlear canal, secondary bony lamina for the basilar membrane, presence:

(0) Absent; (1) Present.

Microtherulum = ?

404. Crista interfenestralis, morphology:

(0) Horizontal, broad, and extending to the base of the paroccipital process;

(1) Vertical, delimiting the back of the promontorium;

(2) Horizontal, narrow, and connecting to the caudal tympanic process.

Microtherulum = 2

405. Post-promontorial tympanic recess, presence:

(0) Absent; (1) Present.

Microtherulum = ?

406. Petrosal, rostral tympanic process, development:

(0) Absent or low ridge;

(1) Tall ridge, but restricted to the posterior half of the promontorium;

(2) Well-developed ridge reaching the anterior pole of the promontorium.

Microtherulum = ?

407. Petrosal, caudal tympanic process, presence:

(0) Absent; (1) Present;

Microtherulum = ?

408. Petrosal, caudal tympanic process, notched:

(0) Absent; (1) Present;

Microtherulum = ?

409. Petrosal, caudal tympanic process, hypertrophied and buttressed against the exoccipital paracondylar process:

(0) Absent; (1) Present;

Microtherulum = ?

410. Rear margin of the auditory region:

(0) Marked by a steep wall;

(1) Extended onto a flat surface.

Microtherulum = ?

411. Prootic canal, presence:

(0) Absent; (1) Present.

Microtherulum = ?

412. Prootic canal, orietation:

(0) Vertical;

(1) Horizontal and reduced.

Microtherulum = ?

413. Sulcus for the anterior distributary of the transverse sinus, position, relative to the subarcuate fossa:

(0) Anterolateral;

(1) Posterolateral.

Microtherulum = ?

414. Lateral trough floor anterior to the tympanic aperture of the prootic canal and/or the primary facial foramen:

(0) Open lateral trough, no bony floor;

(1) Bony floor present;

(2) Lateral trough absent.

Microtherulum = ?

415. Cavum epiptericum, anteroventral opening, morphology:

(0) Present;

(1) Present, with reduced size (due to the anterior expansion of the lateral trough floor);

(2) Present, partially enclosed by the petrosal;

(3) Present, enclosed by the alisphenoid and petrosal;

(4) Present, as large piriform fenestra;

Microtherulum = ?

416. Geniculate ganglion, enclosure by the bony floor of the petrosal in the cavum supracochleare, presence:

(0) Absent; (1) Present.

Microtherulum = ?

417. Hiatus Fallopii:

(0) Present, in the petrosal roof of the middle ear;

(1) Present, at the anterior end of the petrosal;

(2) Absent (applicable only to those taxa with a cavum supracochleare);

(3) Endocranial.

Microtherulum = ?

418. Foramen ovale, composition:

(0) Between the petrosal and alisphenoid;

(1) Secondary foramen partially or fully enclosed by the alisphenoid, in addition to the primary foramen between the petrosal and alisphenoid;

(2) In the petrosal (anterior lamina);

(3) Between the alisphenoid and squamosal;

(4) Within the alisphenoid.

Microtherulum = ?

419. Foramen ovale, position:

(0) On the lateral wall of the braincase;

(1) On the ventral surface of the skull.

Microtherulum = ?

420. Exit(s) for the mandibular branch of the trigeminal nerve (V3), number:

(0) One;

(1) Two.

Microtherulum = ?

421. Alisphenoid, quadrate ramus:

(0) Forming a rod underlying the anterior part of the lateral flange;

(1) Absent;

(2) Present, mostly laminar process in vicinity of foramen ovale.

Microtherulum = ?

422. Alisphenoid, alisphenoid canal (for the ramus inferior and/or ramus infraorbitalis), presence:

(0) Absent;

(1) Present.

Microtherulum = ?

423. Anterior lamina, exposure on the lateral braincase wall, presence:

(0) Present; (1) Reduced or absent.

Microtherulum = ?

424. Anterior part of the lateral flange, orientation:

(0) Horizontal shelf;

(1) Ventrally directed;

(2) Medially directed and contacting the promontorium;

(3) Vestigial or absent.

Microtherulum = ?

425. Lateral flange, vertical component ('L-shaped' and forming a vertical wall to the pterygoparoccipital foramen), presence:

(0) Present; (1) Absent.

Microtherulum = ?

426. Lateral flange, vascular foramen in the posterior part (and anterior to the pterygoparoccipital

foramen), presence:

(0) Present; (1) Absent.

Microtherulum = ?

427. Lateral flange, relationship to the crista parotica (or the anterior paroccipital process that bears the crista):

(0) Widely separated;

(1) Narrowly separated;

(2) Continuous.

Microtherulum = ?

428. Pterygoparoccipital foramen (for the ramus superior of the stapedial artery):

(0) Laterally open notch;

(1) Foramen enclosed by the petrosal or squamosal;

(2) Absent.

Microtherulum = ?

429. Pterygoparoccipital foramen, position, relative to the level of the fenestra vestibuli:

(0) Posterior or lateral;

(1) Anterior.

Microtherulum = ?

430. "Bifurcation of the paroccipital process", presence:

(0) Absent; (1) Present.

Microtherulum = ?

431. Petrosal, posterior paroccipital process:

(0) No ventral projection below the level of the surrounding structures;

(1) Projecting below the surrounding structures.

Microtherulum = ?

432. Anterior paroccipital region, morphology:

(0) Anterior paroccipital is bulbous and distinctive from the surrounding structures;

(1) Anterior paroccipital region has a distinct crista parotica.

Microtherulum = ?

433. Epitympanic recess lateral to the crista parotica, presence:

(0) Absent; (1) Present.

Microtherulum = ?

434. Tympanohyal contact with the cochlear housing, presence:

(0) Absent; (1) Present.

Microtherulum = ?

435. Relationship of the squamosal to the paroccipital process:

(0) Squamosal covers the entire paroccipital region;

(1) No squamosal cover on the anterior paroccipital region;

(2) Squamosal covers a part of the paroccipital region, but not the crista parotica (the squamosal wall and the crista parotica are separated by the epitympanic recess).

Microtherulum = ?

436. Squamosal, medial process, reaching toward the tympanic cavity, presence:

(0) Absent;

(1) Present (near or bordering on the foramen ovale).

Microtherulum = ?

437. Petrosal, stapedial artery sulcus, presence:

(0) Absent; (1) Present.

Microtherulum = 0

438. Cochlear housing, transpromontorial sulcus for the internal carotid artery, presence:

(0) Absent; (1) Present.

Microtherulum = ?

439. Promontorium, deep groove on the anterior pole, presence:

(0) Absent; (1) Present.

Microtherulum = 0

440. Perbullar canal or sulcus for the internal carotid artery, presence:

(0) Absent; (1) Present.

Microtherulum = ?

441. Epitympanic wing medial to the promontorium, presence:

(0) Absent; (1) Present.

Microtherulum = ?

442. Alisphenoid, ectopterygoid process, presence:

(0) Absent; (1) Present.

Microtherulum = ?

443. Alisphenoid, tympanic process, development:

(0) Absent;

(1) Present, but limited to the "piriform" region of the basicranium;

(2) Intermediate;

(3) Well-developed, extending to near the jugular foramen

Microtherulum = ?

444. Hypotympanic recess in the junction of the alisphenoid, squamosal, and petrosal, presence:

(0) Absent; (1) Present.

Microtherulum = ?

445. Fenestra cochleae, separation of the from the jugular foramen:

(0) Absent;

(1) Separate but within the same depression;

(2) Separate (not within the same depression).

Microtherulum = 1

446. Channel of the perilymphatic duct:

(0) Open channel and sulcus;

(1) At least partially enclosed channel.

Microtherulum = ?

447. Jugular foramen, size relative to the fenestra cochleae:

(0) Jugular subequal to the fenestra cochleae;

(1) Jugular larger than the fenestra cochleae.

Microtherulum = ?

This character is applicable only to those taxa with a jugular foramen fully separated from the fenestra cochleae.

448. Jugular forame, relationship to the opening of the inferior petrosal sinus:

(0) Confluent; (1) Separate.

Microtherulum = ?

449. Stapedial muscle fossa, presence:

(0) Absent;

(1) Present.

Microtherulum =1

450. Stapedial muscle fossa, size:

(0) Small;

(1) Large (twice the size of the fenestra vestibuli).

Microtherulum = ?

451. Stapedial fossa, alignment, relative to the crista interfenestralis:

(0) Aligned with crista interfenestralis;

(1) Lateral to the crista interfenestralis

Microtherulum = 1

452. Hypoglossal foramen:

(0) Indistinct, either confluent with the jugular foramen or sharing a depression with the jugular foramen;

(1) Separated from the jugular foramen;

(2) Separated from the jugular foramen; the latter has a circular, raised external rim.

Microtherulum = 1

453. Hypoglossal foramina, number:

(0) Single; (1) Double.

Microtherulum = ?

Middle Ear Ossicle Characters:

454. Incudomallear contact, shape:

(0) Strongly convex surface on the incus;

(1) Strongly convex surface on the incus with trough perpendicular to it;

(2) Weakly convex or flat surface on the incus;

(3) Saddle-shaped contact on the incus;

Microtherulum = 3

455. Incus and the malleus, alignment:

(0) Posterior-anterior;

(1) Posteromedial to anterolateral;

(2) Dorsoventral.

Microtherulum = 1

456. Incus, incudal body, shape (including the mallear articular surface):

(0) Cylindrical;

(1) Plate-like;

(2) Globular.

Microtherulum = 2

457. Incudomallear contact, orientation:

(0) Primary axis of the articular surface on the incus is perpendicular to the plane of the mallear body;

(1) Primary axis of the articular surface on the incus is in the same plane as the mallear body.

Microtherulum = 0

This character was scored as “state (2)” for many therian taxa in Wang et al. (2021) and here these scorings are changed to state (0) based on personal communication to Dr. John Wible.

458. Incudal articular surface and mallear body, overlapping, extent:

(0) Complete; (1) Partial.

Microtherulum = NA

This character is applicable only to taxa with the incudal articular surface in the same plane as the mallear body.

459. Quadrate/incus, dorsal plate (= crus brevis), shape:

(0) Broad plate; (1) Pointed triangle; (2) Reduced.

Microtherulum = 1

460. Incus, angle of the crus brevis to crus longum of the incus (this is equivalent to the angle between the dorsal plate and the stapedial process of the quadrate):

(0) Alignment of the stapedial process (crus longum) and the dorsal plate (crus brevis) (or an obtuse angle between the two structure) (distinctive process is lacking, stapes/incus contact is on the medial side of the quadrate trochlea);

(1) Perpendicular or acute angle of the crus brevis and crus longum (“A-shaped” incus).

Microtherulum = 1

461. Quadrate/incus, primary suspension on the basicranium:

(0) By quadratojugal in addition to at least one other basicranial bone;

(1) By squamosal only;

(2) By petrosal (either by the preserved direct contact of the incus or by inference from the presence of a well-defined crista parotica).

Microtherulum = ?

462. Quadratojugal, presence:

(0) Present; (1) Absent.

Microtherulum = 1

463. Stapes, morpholoty:

(0) Columelliform-macroperforate;

(1) Columelliform-imperforate (or microperforate);

(2) Bicrurate-perforate;

(3) Bicrurate-perforate with a large posterior process (or the process for insertion of the stapedius muscle [PISM]).

Microtherulum = 2

464. Stapes, stapedial ratio (length/width of oval window or footplate):

(0) Less than 1.4;

(1) 1.4-1.8;

(2) ≥1.8.

Microtherulum = 2

The stapedial ratio is about 1.82 in *Microtherulum*.

465. Stapes, bullate stapedial footplate, presence:

(0) Absent;

(1) Present.

Microtherulum = 0

466. Malleus, malleolar neck, presence:

(0) Absent;

(1) Present.

Microtherulum = 1

467. Malleus, length of the malleus manubrium:

(0) Shorter than the combined width of the surangular and prearticular anterior to the incudo-malleolar joint;

(1) Longer than the combined width of surangular and prearticular.

Microtherulum = ?

468. Malleus, malleolar manubrium, thickness:

(0) Robust;

(1) Gracile.

Microtherulum = ?

469. Meckel's bone (=anterior portion of postdentary rod), distinctive angle or bending anterior to the level of ectotympanic (angular) bone, presence:

(0) Absent;

(1) Present.

Microtherulum = NA

470. Meckel's cartilage, articulation with the posterior (pterygoid) region of mandible (medio-lateral contact vs. separation of Meckel's element, either independent or as an ossified component of the "postdentary rod", from the posterior (pterygoid) region of mandible):

(0) Presence of medio-lateral contact either in adult or in embryonic stage until Meckel's cartilage re-absorption;

(1) Embryonic Meckel's cartilage medio-laterally separated from the posterior part of mandible;

(2) Ossified Meckel's cartilage mediolaterally separated from the posterior part of mandible:

Microtherulum = ?

471. Ectotympanic, size/shape:

(0) Plate-like;

(1) Curved and rod-like;

(2) Ring-shaped;

(3) Slightly expanded (fusiform);

(4) Expanded;

(5) Tube-like.

Microtherulum = 3

472. Ectotympanic arc:

(0) About 70 degrees:

(1) $\leq 90 - 135$ degrees;

(2) ≥ 135 degrees.

Microtherulum = 2

473. Ectotympanic, anterior process, presence:

(0) Present; (1) Absent.

Microtherulum = 1

474. Incisura tympanica, position/orientation:

(0) Posteroventral;

(1) Posterior;

(2) Postero-dorsal;

(3) Dorsal.

Microtherulum = ?

475. Ectotympanic, fusion to other cranial bones, presence:

(0) Absent;

(1) Fused to other bones.

Microtherulum = 0

476. Entotympanic, contribution to the bullar structure, presence:

(0) Absent; (1) Present.

Microtherulum = ?

Other Cranial Characters:

477. Bony secondary palate, posterior extent:

(0) Anterior to the posterior end of the tooth row;

(1) Level with the posterior end of the tooth row;

(2) Extending posterior to the tooth row;

(3) Extending to the basisphenoid-basioccipital suture.

Microtherulum = ?

478. Palate, posterior median spine, presence:

(0) Absent;

(1) Present.

Microtherulum = ?

479. Pterygopalatine ridges, presence:

(0) Present; (1) Absent.

Microtherulum = ?

480. Pterygoid, transverse process, development:

(0) Present and massive;

(1) Present but reduced (as the hamulus);

(2) Greatly reduced (with a vestigial crest on pterygoid) or absent.

Microtherulum = ?

481. Pterygoid, contact on midline on pharyngeal roof, presence:

(0) Present; (1) Absent.

Microtherulum = ?

482. Minor palatine foramen, ventral opening, composition:

(0) Encircled by the pterygoid (and ectopterygoid if present) in addition to the palatine;

(1) Encircled by the palatine and maxilla, separated widely from the subtemporal margin;

(2) Encircled completely by the palatine (or between palatine and maxilla), large, with thin bony bridge from the subtemporal margin;

(3) Large, posterior fenestration;

(4) Notch.

Microtherulum = ?

483. Transverse canal foramen, presence:

(0) Absent; (1) Present.

Microtherulum = ?

484. Carotid foramen, position:

(0) Within the basisphenoid;

(1) Within the basisphenoid/basioccipital suture;

(2) Within the basisphenoid/petrosal suture;

(3) Through the opening of the cavum epiptericum.

Microtherulum = ?

485. Orbit, overhanging roof, presence:

(0) Absent;

(1) Present, formed by the frontal.

Microtherulum = ?

486. Infraorbital canal, exit(s), number:

(0) Single; (1) Multiple.

Microtherulum = ?

487. Infraorbital canal, posterior opening (maxillary foramen), composition:

(0) Between the lacrimal, palatine, and maxilla;

(1) Exclusively enclosed by the maxilla;

(2) Enclosed by the maxilla, frontal and palatine.

Microtherulum = ?

488. Lacrimal, shape:

(0) Small, oblong-shaped on the facial part of the rostrum;

(1) Large, triangle-shaped on the facial portion of rostrum;

(2) Crescent shaped on the facial portion of the rostrum;

(3) Reduced to a narrow strap;

(4) Absent from the facial portion of the rostrum.

Microtherulum = ?

489. Lacrimal foramen, location:

(0) Within the orbit;

(1) On the facial side of the lacrimal (anterior to or on the anterior orbital margin).

Microtherulum = ?

490. Lacrimal foramina, number:

(0) One;

(1) Two.

Microtherulum = ?

491. Lacrimal foramen, composition:

(0) Within the lacrimal;

(1) Bordered by or within the maxilla.

Microtherulum = ?

492. Zygomatic arch, maximum vertical depth, relative to the length of the skull (this character is designed to indicate the robust vs. gracile nature of the zygomatic arch):

(0) Between 10-20%;

(1) Between 5-7%;

(2) Zygoma incomplete.

Microtherulum = ?

493. Ultimate upper molar, implanted in the anterior root of zygoma, presence:

(0) Absent;

(1) Present.

Microtherulum = 0

494. Frontal and alisphenoid, contact:

(0) Present;

(1) Absent.

Microtherulum = ?

495. Frontal and alisphenoid, contact:

(0) Dorsal plate of the alisphenoid contacting the frontal at the anterior corner;

(1) Dorsal plate of the alisphenoid with more extensive contact with the frontal (~50% of its dorsal border);

(2) Absent.

Microtherulum = ?

496. Frontal and maxilla, facial contact, presence:

(0) Absent;

(1) Present.

Microtherulum = 0

497. Nasal and frontal, suture, medial process of the frontals wedged between two nasals, presence:

(0) Absent;

(1) Present.

Microtherulum = 1

498. Nasal, posterior width:

(0) Narrow;

(1) Broader than the width at the mid-length of the nasal.

Microtherulum = 0

499. Pila antotica, presence:

(0) Present;

(1) Absent.

Microtherulum = ?

500. Orbitosphenoid, medial orbital wall fully ossified, presence:

(0) Absent;

(1) Present.

Microtherulum = ?

501. Orbitosphenoid, medial orbital wall fully ossified, contribution to the braincase floor and the medial orbital wall:

(0) Forming the ventral floor of the braincase but not the entire orbital wall;

(2) Forming both the braincase floor and the medial orbital wall.

Microtherulum = ?

502. Optic foramen, separation from the sphenorbital fissure, presence:

(0) Absent;

(1) Present.

Microtherulum = ?

503. Exit for maxillary nerve, separation from sphenorbital fissure, presence:

(0) Present;

(1) Absent (confluent with sphenorbital fissure).

Microtherulum = ?

504. Exit for maxillary nerve, separation from sphenorbital fissure, placement:

(0) Separate from sphenorbital fissure, behind alisphenoid;

(1) Separate from sphenorbital fissure, within alisphenoid;

Microtherulum = ?

505. Orbitotemporal canal, separate anterior opening, presence:

(0) Absent;

(1) Present.

Microtherulum = ?

506. Minor palatine nerve, orbital opening, presence:

(0) Absent;

(1) Present.

Microtherulum = ?

507. Zygoma, anterior part of the jugal, contribution to the orbit:

(0) Anterior part of the jugal extends to the facial part of the maxilla and forms a part of the anterior orbit;

(1) Anterior part of the jugal does not reach the facial part of the maxilla and is excluded from the anterior orbit margin.

Microtherulum = 0

508. Jugal, posterior part, contribution to the squamosal glenoid:

(0) Contributes to the squamosal glenoid;

(1) Borders on but does not contribute to the squamosal glenoid;

(2) Terminates anterior to the squamosal glenoid.

Microtherulum = 2

509. Maxilla in the sub-temporal margin of the orbit:

(0) Absent;

(1) Present and not extensive;

(2) Present and extensive.

Microtherulum = 2

510. Frontal, orbital process of the frontal borders on the maxilla within orbit, presence:

(0) Absent;

(1) Present.

Microtherulum = ?

511. Anterior ascending vascular channel (for the arteria diploëtica magna) in the temporal region, presence:

(0) Present;

(1) Absent.

Microtherulum = ?

512. Anterior ascending vascular channel (for the arteria diploëtica magna) in the temporal region, form:

(0) Open groove;

(1) Partially enclosed in a canal;

(2) Completely enclosed in a canal or endocranial;

Microtherulum = ?

513. Posttemporal canal for the arteria and vena diploëtica, :

(0) Present, large;

(1) Small;

(2) Absent.

Microtherulum = ?

514. Nuchal crest:

(0) Overhanging the concave or straight supraoccipital;

(1) Weakly developed with convex supraoccipital.

Microtherulum = ?

515. Sagittal crest, development:

(0) Prominently developed;

(1) Weakly developed;

(2) Absent.

Microtherulum = 1

516. Tabular bone, presence:

(0) Present; (1) Absent.

Microtherulum = ?

517. Occipital slope, orientation:

(0) Occiput sloping posterodorsally (or vertically oriented) from the occipital condyle;

(1) Occiput sloping anterodorsally from the occipital condyle (such that the lambdoidal crest is leveled anterior to the occipital condyle and condyle is fully visible in dorsal view of the skull).

Microtherulum = ?

518. Occipital, occipital artery groove extending dorsal to the posttemporal foramen, presence:

(0) Absent;

(1) Present.

Microtherulum = ?

519. Nasal, foramina on the dorsal surface, presence:

(0) Absent;

(1) Present.

Microtherulum = 0

520. Septomaxilla, presence:

(0) Present;

(1) Absent.

Microtherulum = 0

521. Septomaxilla, ventromedial shelf, presence:

(0) Present;

(1) Absent.

Microtherulum = ?

522. Premaxilla, internarial process, presence:

(0) Present;

(1) Absent.

Microtherulum = 1

523. Premaxilla, posterodorsal process:

(0) Does not extend beyond canine ("short or absent");

(1) Extends beyond canine ("intermediate");

(2) Contacts frontal posteriorly (“long”).

Microtherulum = 0

524. Premaxilla, facial part of the premaxilla borders on the nasal, presence:

(0) Absent;

(1) Present.

Microtherulum = 1

525. Premaxilla, palatal process, relative to the canine alveolus:

(0) Does not reach to the level of the canine alveolus;

(1) Reaches the level of the canine alveolus.

Microtherulum = 0

526. Incisive foramina, size:

(0) Small (one or two incisors);

(1) Intermediate (three or four incisors);

(2) Large (more than half the palatal length).

Microtherulum = ?

527. Palatine, palatal vacuities:

(0) Absent;

(1) Present, near palatomaxillary border;

(2) Present, either positioned near or extended to the posterior edge of bony palate.

Microtherulum = ?

528. Major palatine foramina, presence:

(0) Absent;

(1) Present.

Microtherulum = ?

529. Nasal cavity, ossified ethmoidal cribriform plate, presence:

(0) Absent;

(1) Present.

Microtherulum = ?

530. Nasal cavity, posterior excavation of the nasal cavity into the bony sphenoid complex, presence:

(0) Absent;

(1) Present;

(2) Present and partitioned from the nasal cavity.

Microtherulum = ?

Cranial Vault and Brain Endocast Characters:

531. Braincase, external bulging of the braincase in the parietal region, development:

(0) Absent;

(1) Expanded (the parietal part of the cranial vault is wider than the frontal part, but the expansion does not extend to the lambdoidal region);

(2) Greatly expanded (expansion of the cranial vault extends to the lambdoidal region).

Microtherulum = ?

532. Vermis, anterior expansion of the vermis (central lobe of the cerebellum) , presence:

(0) Absent;

(1) Present.

Microtherulum = ?

533. Vermis, overall size:
(0) Small;
(1) Enlarged.
Microtherulum = ?

534. Lateral cerebellar hemisphere (excluding the paraflocculus), presence:
(0) Absent;
(1) Present.
Microtherulum = ?

535. Endocast, external division between the olfactory lobe and the cerebral hemisphere (well-defined transverse sulcus separating the olfactory lobes from the cerebrum), development:
(0) Absence of external separation of the olfactory lobe from cerebral hemisphere;
(1) Enlarged olfactory lobes;
(2) Clear division of transverse sulcus.
Microtherulum = ?

536. Encephalization quotient:
(0) Below 0.13;
(1) Between 0.15-0.25,
(2) Above 0.26.
Microtherulum = ?

537. Expansion of the posterior cerebral hemisphere (for each hemisphere, not the combined width of the posterior hemispheres), presence:
(0) Absent;
(1) Present.
Microtherulum = ?

Soft-tissue Characters:

538. Trophoblasts in the placenta, presence:

(0) Absent;

(1) Present.

Microtherulum = ?

539. Mullerian ducts (oviduct and uterus) pass in between the ureters, presence:

(0) Absent;

(1) Present.

Microtherulum = ?

540. Placenta, presence:

(0) Absent;

(1) Present.

Microtherulum = ?

541. Placenta, types, with vascularized chorioallantois :

(0) Present (placenta with vascularized chorioallantois);

(1) Absent (placenta without vascularized chorioallantois).

Microtherulum = ?

542. Stapes, stapes length:skull length, ratio (based on Wible, 1991):

(0) greater than 7.5%;

(1) less than 5.5%

Microtherulum = 1

543. Stapes, process for insertion of the stapedius muscle (see the discussion on the dorsal process of the stapes), presence:

(0) Absent;

(1) Present.

Microtherulum = 0

544. Stapes, process for insertion of the stapedius muscle, development:

(0) Large (approaching the width or the stapes);

(1) Small or reduced

Microtherulum = 1

545. Lower postcanines, teeth differentiated into premolar (iform) and molar (iform) teeth, presence:

(0) Absent;

(1) Present;

Microtherulum = 1

This character is modified by deleting the state (2) [(2) Secondarily lost the tooth difference or teeth]. Instead, taxa that were scored as state (2) are coded either as inapplicable because of complete loss of teeth (monotremes and *Tamandua*), or state (0) because of lack of differentiation among lower postcanines.

546. Upper postcanines, teeth differentiated into premolariforms and molariforms, presence:

(0) Absent;

(1) Present;

Microtherulum = 1

This character is modified by deleting the state (2) [(2) Secondarily lost the tooth difference or teeth]. Instead, taxa that were scored as state (2) are coded either as inapplicable because of complete loss of teeth (monotremes and *Tamandua*), or state (0) because of lack of differentiation among lower postcanines.

547. Posterior upper molars, triangulation of cusps, types:

(0) Absent;

(1) Two primary rows of multi-cuspate (not counting the secondary cusps);

(2) Three primary multi-rows of multi-cuspate;

(3) Posterior molars triangulated.

Microtherulum = 3

548. M1 cusp, size:

(0) Cusps on all rows subequal;

(1) Primary lingual cusps larger;

(2) Primary labial cusps larger

Microtherulum = NA

This character is applicable to those with multi-cusp rows of multi-cuspate.

549. M2 cusp, size:

(0) Cusps on all rows subequal;

(1) Primary labial cusps larger

Microtherulum = NA

This character is applicable to those with multi-cusp rows of multi-cuspate.

550. M1, primary lingual row, secondary ridge or cusp addition on the primary lingual side:

(0) Absent;

(1) Ridge added;

(2) Cusps added

Microtherulum = NA

This character is applicable to those with multi-cusp rows of multi-cuspate.

551. M2, primary labial row, secondary ridge or cusp addition on the labial side:

(0) Absent;

(1) Ridge;

(2) Cusps

Microtherulum = NA

This character is applicable to those with multi-cusp rows of multi-cuspate.

552. Upper molar (molariform), cusp, shape:

(0) Conical;

(1) Pyramidal;

(2) Crescent (with mesial face concave);

(3) Cusp strongly crested;

(4) Cusp lost.

Microtherulum = 0

553. Lower molariforms, lingual and labial cusp, number:

(0) Equal;

(1) Unequal

Microtherulum = ?

This character is applicable to those with multi-cusp rows of multi-cuspate.

554. Upper premolar-molar series, stepwise pattern ("en echelon" pattern of Jenkins et al., 1997), presence:

(0) Absent;

(1) Present.

Microtherulum = ?

This is applicable only to molars with more than one rows of multiple cusps.

Characters from Mao et al. (2021)

555. Surangular bone in adult individual:

(0) Present;

(1) Absent.

Microtherulum = 1

556. Craniomandibular articulation, composition:

(0) Quadrate/articular;

(1) Primarily quadrate/articular, secondarily surangular/squamosal;

(2) Incipient dentary/squamosal;

(3) Primarily dentary/squamosal;

(4) Exclusively dentary/squamosal.

Microtherulum = 4

557. Molar(iform), three distinct main cusps (A, B, C or a, b, c), development:

(0) Absent;

(1) Initial;

(2) Present. (verified and complete states)

There is no state (2) in Mao et al. (2021) and some non-mammaliaform taxa are coded as state (1) in the character matrix. Here the complete state series is listed based on personal communications to Jin Meng.

Microtherulum = 2

558. Lower molar(iform), two rows of cusps, development:

(0) Absent;

(1) Present.

Microtherulum = 0

559. Upper molar(iform), primary cusp row, number:

(0) Two rows;

(1) Three rows.

Microtherulum = NA

This character is applicable to those with two or three cusp rows.

560. m1 (or one of the distal lower postcanines), cusps, total number:

(0) 4 or fewer; (1) 5-8; (2) 9-12; (3) 13 or more.

Microtherulum = NA

This character is only applicable to taxa with multi-rowed postcanines.

561. m1-2, protoconid (cusp a), outline:

(0) Buccal curvature at base level relative to curvature of paraconid (cusp b) and metaconid (cusp c) cusps have same degree of labial bulging;

(1) Protoconid far more bulging than paraconid and metaconid.

Microtherulum = 1

562. m1-2, protocristid crest between protoconid (cusp a) and metaconid (cusp c), orientation relative to long axis of lower molars:

(0) Parallel to lower jaw axis;

(1) Oblique;

(2) Transverse.

Microtherulum = 2

563. m1, main cusps of trigonid, alignment:

(0) Single longitudinal row;

(1) Obtuse angle;

(2) Acute angle.

Microtherulum = 2

This character is not applicable to taxa with multi-row cheek teeth.

564. m1-2, paraconid (cusp b), presence:

(0) Present;

(1) Absent.

Microtherulum = 0

This character is not applicable to taxa with multi-row cheek teeth.

565. Crest connecting main cusp a to lingual cingulid cusp g or the cusp g position, presence:

(0) Absent;

(1) Present.

Microtherulum = 0

566. m1-2, talonid, hypoconulid, mediolateral position:

(0) At median position;

(1) At more lingual position.

Microtherulum = 0

This character is only applicable to taxa with multicuspidate talonid developed.

567. m1-2, prehypoconulid, crest connecting metaconid with hypoconulid along lingual edge of tooth, presence:

(0) Absent;

(1) Present.

Microtherulum = 0

568. M1-2 (or distal postcanines), lingual cingulum, presence:

(0) Presence;

(1) Vestigial or absent.

Microtherulum = 1

569. M1-2 (or distal postcanines), lingual cingulum, development:

(0) Continuous cingulum;

(1) Discontinuous cingulum.

Microtherulum = NA

570. M1, primary buccal cusp row, wear facet distributions:

(0) On buccal side of all buccal cusps;

(1) On buccal side of A1, but not on the buccal side of the mesiobuccal cusp; (2) On top of cusps.

Microtherulum = NA

This character is for those with multicusp rows and with molar-premolar differentiation.

571. Lower incisor, i1, position of distal end of root, relative to postcanines:

(0) Mesial to level of premolars;

(1) Opposite level of premolars;

(2) Opposite level of molars.

Microtherulum = 0

572. Lower postcanine, root, curving distally, presence:

(0) Absent;

(1) Present.

Microtherulum = 0

573. Cervical vertebrae, atlas neural hemiarches:

(0) Unfused;

(1) Fused.

Microtherulum = ?

574. Humerus, ectepicondylar foramen, presence:

(0) Present;

(1) Absent.

Microtherulum = ?

575. Humerus, supratrochlear foramen, presence:

(0) Absent;

(1) Present.

Microtherulum = ?

576. Ulna, olecranon process, ossification:

(0) Unossified or poorly ossified;

(1) Well ossified.

Microtherulum = ?

577. Ulna, olecranon process, length relative to semilunar (=trochlear, sigmoid) notch:

(0) Olecranon shorter than notch;

(1) olecranon roughly equal to notch;

(2) olecranon longer than notch.

Microtherulum = ?

578. Ulna, olecranon process, posterior inflection, presence:

(0) Absent;

(1) Present.

Microtherulum = ?

579. Ulna, styloid process, presence:

(0) Absent;

(1) Present.

Microtherulum = ?

580. Phalanges in manual digit III, number:

(0) Four;

(1) Three;

(2) Two.

Microtherulum = ?

581. Phalanges in manual digit IV, number:

(0) Four;

(1) Three;

(2) Two.

Microtherulum = ?

582. Obturator foramen, size:

(0) Smaller or equal in size to acetabulum;

(1) Intermediate, larger than acetabulum but less than twice size;

(2) Large, more than twice size of acetabulum.

Microtherulum = 1

583. Femur, femur neck, degree of development:

(0) Incipient and short;

(1) Distinct and long.

Microtherulum = 1

584. Femur, greater trochanter, separation:

(0) Continuous with femoral head;

(1) Separated from femoral head by distinct notch.

Microtherulum = 1

585. Femur patellar groove mediolateral contour, shape:

(0) Flat;

(1) Concave.

Microtherulum = ?

586. Squamosal, glenoid fossa for dentary condyle, outline:

(0) Outline subcircular;

(1) Oval, long axis anteroposterior;

(2) Oval, long axis mediolateral.

Microtherulum = 2

587. Prootic and opisthotic, separation:

(0) Separated;

(1) Fused at early ontogenetic stage to form petrosal (=periotic).

Microtherulum = ?

588. Quadrate, articulation with stapes:

(0) Stapedial contact restricted to medial end of trochlea;

(1) Via projection from medial margin of dorsal plate;

(2) Via medial vertical ridge on neck of quadrate;

(3) Via projection from neck of quadrate;

(4) Via stapedial process of incus.

Microtherulum = 4

589. Interparietal (postparietal) in adult, presence:

(0) Present (separate bone);

(1) Absent or fused with other bones.

Microtherulum = 1

590. Prefrontal, presence:

(0) Present;

(1) Absent.

Microtherulum = 1

591. Postorbital bone, presence:

(0) Present;

(1) Absent.

Microtherulum = ?

592. Incisive foramen, vomer exposure (at anterior ends of maxillae on palate), presence:

(0) Present;

(1) Absent.

Microtherulum = ?

593. Internal auditory meatus:

(0) Open;

(1) Walled.

Microtherulum = 1

594. Ectopterygoid bone, presence:

(0) Present;

(1) Absent.

Microtherulum = ?

595. Lacrimal facial process, contact with septomaxilla:

(0) Absent;

(1) Present.

Microtherulum = ?

596. Occipital condyle, shape (in lateral view):

(0) Bulbous;

(1) Ovoid to cylindrical.

Microtherulum = 1

597. Septomaxilla, facial process, contact with maxilla, presence:

(0) Present; (1) Absent.

Microtherulum = ?

Characters from Zhou et al. (2019)

598. Clavicle, contact of medial ends of two clavicles at the midline, presence:

(0) Present (point-contact or abutting contact);

(1) Absent (two clavicles respectively contact the interclavicle/sternal manubrium).

(Note: All extant marsupials and placentals should be scored 1)

Microtherulum = ?

599. Urogenital sinus and vagina, morphological differentiation, presence:

(0) Absent (presence of cloaca, no differentiation of vagina fromurogenital sinus, the latter confluent with rectum);

(1) Present (differentiation of vagina from urogenital sinus).

Microtherulum = ?

600. Vas deferens looping over ureter in adult male, presence:

(0) Absent (in *Ornithorhynchus*, *Tachyglossus*, all extant marsupial species);

(1) Present (in all extant placental species)

Microtherulum = ?

601. Descent of testis and location of testes in adult males:

(0) No descent of testis (testes abdominal, or testicondy) (in *Ornithorhynchus*, *Tachyglossus*);

(1) Testis descended either ascrotal (testes in pelvic or inguinal position, but ascrotal and not in an external scrotum)

Microtherulum = ?

602. Scrotum, development:

(0) Acrotal-testes in abdominal, pelvic, or inguinal position;

(1) Scrotal-testes in scrotum.

Microtherulum = ?

This character is not observable in fossil mammals.

603. Basihyal, ossification, presence:

(0) Absent;

(1) Present.

Microtherulum = ?

604. Basihyal, shape:

(0) Rod-like;

(1) Antero-posteriorly wide.

Microtherulum = ?

605. Hyoid apparatus, anterior cornu, composition:

(0) Formed by a single, elongate hyoid rod without internal segmentation;

(1) Jointed short segments of anterior cornua (scored for this character state where the ceratobasihyal joint is preserved, and scored also if the ceratoepihyal joint or the dorsal end of ceratohyal is preserved.

Microtherulum = ?

606. Thyrohyals, ossification, presence:

(0) Not ossified;

(1) Ossified.

Microtherulum = ?

607. Thyrohyals, shape:

(0) Rod-like along the shaft;

(1) Strap-like in mid shalf;

(2) Oblong plate.

Microtherulum = ?

608. Thyrohyal, dorsal end, expansion:

(0) Absent;

(1) Present.

Microtherulum = ?

609. Thyrohyal, dorsal end, expansion, shape:

(0) Club-like or fan-like;

(1) Broad and rhomboidal or semicircle-like.

Microtherulum = ?

610. Basihyal and thyrohyals, fusion:

(0) Unfused;

(1) Fused.

Microtherulum = ?

611. Fused basihyal and thyrohyals, forming an angled “V-bone”, presence:

(0) Absent;

(1) Present.

Microtherulum = ?

612. Ceratohyal, shape:

(0) Rod-like;

(1) Strap-like;

(2) Plate-like;

(3) Shortened and block-like.

Microtherulum = ?

613. Epihyal, either cartilaginous or ossified in adult, presence:

(0) Absent;

(1) Present.

Microtherulum = ?

614. Ossified stylohyal in adult, presence:

(0) Absent;

(1) Present.

Microtherulum = ?

615. Hyoid apparatus, anterior cornu, morphotypes:

(0) Discreto-cornuate

(1) Integro-cornuate

Microtherulum = ?

Character matrix for mammaliaform phylogeny

Thrinaxodon

0000000?0000000000000?00000000??0?0?0?0100000???????????????????????
?????????????????????0?0??????????????000000?00???0??????????????????????????
?????????????????????????????????00?????????????????000000000000000???00??0?
00000001?????????0000000001000000000000?0?00000?0000000000000000000?0000
0?0??????000000??000000000000?0000000?0000?0000?00000000000?000000011
0000000???00?0000000?00?????????0000??000??00?0??00??000?0000010?100000
0??0????0?00?00??0??0?0000?00000000000000000000000000000000000000000?
000000?000000000000000000100000000000????0??000????????010?????????????00
000??000??0??000000000000????0?00?????????

Massetognathus

0000010?000000000000100?0000000???0?0?000100000???????????????????????
?????????????????????0?13??????????????000000?00???????????????????????????????
?????????????????????????????????11?????????????????000000000000000???00??0?
000000010?0?????0?0000??????000?000??0?0?????0?????00?????0000000?00000?
0??????1000?0??00000???0??0????0?00???????????????????????????????????????
???0????0??00?00?????????1000??000??00?0??00??000?000001001000000??0????0
?00?00??0??0?1000?000000000000010000000000000300000000000000001000000?00
000000000??000000000000000010????0??000??????0?100????????????0??00??00??0
?0??00000101?0?0?????????????????

Probainognathus

0000000?0000000000100?0000000???000?000100000???????????????????????
?????????????????????0?0??????????????000000?00???0??????????????????????????
?????????????????????????????????00?????????????????000000000000000???00??0?
0000000???????????0000??????010?000??0?0?0???0?????00????????????0?????0???
?????10???0??000?0???0??0????0?00???
?????0??00?00?????0???1000??000??00?0??00??000?000001001100000??0????0?0
0?00??0??0?0000?00000?0000001000000000000000000000000000001000000?0000
0000000??000000000000000101???????000????????110?????????????0???00000????0?
?000001010000????????????????????

Tritylodontids

0000010?0000000000000000?0000010???000?30002000000??????????0??????????

????????????????????1113000?00???????000000?00???????????????????????????
??????????????????????????????????23????????????????2?030000l???1????0000??
0?10??11120?000000l0000100000?010?0000000000000000000000000000[0
1]1000000?0?????0200001 0?000000000000?0000000?0000?00?0?00000000000000
0000001??000?0?00?00?0000000?00?0???0??01111??1000000?0??00?0000?000001
000000100??000000?00000?01000?0000?000000000000100000000[0
1]010301000000000000021000100?100000000000000[0
1]00000100?0111???????0020000200?0011[0 1]???????0??010101[0
1]0011101??1111111100[0 1]0?????????????????
Pachygenelus
0000000?0000001000000?0000000?0?000?200001000??????????????????????
????????????0????????0?0??????????????000000?00???0???????????????????????
????????????????????????????????00???????????????300300000100000??0000??1
?20000001??????????0000000?0??10???0000100?00000?000000000000000100000
0?010000?210001????00?0000000??????00??????????????????????????????????
??????????0000000100?0?0??01111??1?00000?0??00??000?00000101100000 0??00
0000?00010000??1?0000?0011000000000100000000100000101010100000000100000
00?10000000000000000000000????????????000????????200??????0??0??0?00010
00??1?1?0021111111001?????????????????
Adelobasileus
??
??????0????????????0??????????????000000?00??000?????????????????????????????
????????????????????????????00??????????????????0????????????????????????????
??????????????0??
??????0???0000
0??????????0221100?????0000??010?11??1010000100l0000???00000000010000??
10??????????0????????????0??0?0?000????????000??011000??????00001100???????
?????0?????????????????????????20?????????????????????????????1???????????????
?????????????
Sinoconodon
00000010000000000000?000000010100100000001000??00??????00?11000110
0?00?00?0?0??00?????????0?0????????0?20???00[0
2]000?00?1000???00?00??????0??????0??000???0?????????0??????????000000????000?

0?00???????0000000000000000003311220020000000001??????????100100??0?01??
???0010000000000000000000000000001000000?0??????????????????000?00??0???
00??000000010000?0?00221100
100000000??01001110101000000000100?000000000000100010010101000?001100000
000100000010010100010100010000000011000010020000000000100000000000100001 0
2????1??110????0???320??00000??10?00??0??????????21?111??100?0????????????
?????
Morganucodon 000010100000000000000?00000011[0
1]?0100000001000??00?0000001?010001101100?00?0?0??00??0?????1010??????0?
20???200000?0110000???00?00?????0?????0??400???0????????0?????????0000010
0??110?0?00???????000[0 1]00[0 1]0000000000000[2 3][1
2]00000000011001??????????000[1
2]00000??11?1?00??1???00100?00000?00000000001000000?0??????2100010???00
00100000?10?0000??000?0000?000000000000000000000???0????????0???00000010
100[0 1][0
1]?0?00231111201000000??0100111010100011010011100100000000001000100100
000?002100000000100000100101000101010100000001100001002000001100101000
0000001000011 2????1??110????0???320??00000??0??000101000??1?102?3111110
01??????1????????????
Megazostrodon
0000100?00000000000000?0000001 10?020010000100 0??00?0000?01?00000110
1100?00?0?0??00??0?????1011??????0?20???200000?0100000??00?00?????0?????
0??400???0????????0?????????00000100??110?0?00???????000100100000000000
01100000000010001??????????20?200000?0110100???????0?100000000?00000000
001000000???00??0210001 0?000000100000??000000?0000?00?0?00000000?00??0
??0?0?1?000000000?00?0000?0010100???0?0023110120 10?0000??0100111010000
011?1001110?10000000001000 1001?0000?0?21???0??001?000010?1??0001010101
000000?11?0001002000001?00????????00??0?????????????110????0????20??00000
??0??00??01000??1??????1??1??0????????????????????
Haldanodon
000001100001000010010?0000000111001001000110 00??00?0000000?01000110
110101010?0??00??0?????1010?0????0020???200031100?0000???00?00?????0????
?0??11????0????????0?????????03320121??110?0?00???????000000100000010012

200000000000011000??????????0002?????????????????????100101000?000?00000
1210000100??????21?0???????000?00000??0?0?0??????????????????????????????
?????????????0????0000010200 11?0?0023110020100000 0??010?1210100?001????0
1010010000000001001100100000???21000???00??00002011?1000101010100000000
1100001002001001110101000000000 0???2?????????110????0????20??01201??1??0
0??011001???1?0????11[0 1]0????????????????????

Castorocauda

00?001100001???01?000?0000001110010010000 1000??00?0???0???10001101
10101000????00??0?????1010?0????002????200031100?0000???00?00?????0?????0
??11????????????????????????030?0?1???110?0?00???????000???100??????011?00?
?0??10??011000???????????002??????000?000???????????????????????000012100
0010000000021000000000000000000?1011000?0100?01?1010100000000000000?0
0?0??0?00000?010100???010???
????????????????????????????????1110010000?????????0??????10??000?11?????0
?2?????1???101?00????????????????????110????0????20??01201??1???0???11?011
??100?????1???????????????????????

Docofossor

0000010?0000000011?00?0000001110010030001 1000??00?00?0000?01000110
110101000?0??00??0?????1010?0????0020???201031100?0000???00?00?????0????
?0??11????0??????????????????03320121??110?0?00???????0001001000000010012
23231110000011000???????????002??????010?000??????????????????????????????
?000010001101021000?0?0000?0000000?1011001 10100?010101010000000000000
00001000000010100 10001????1???
???000???????????
?????????????01000????????????????????110????0????20??01201??10??0??011002
2[0 1]?100?????1??????????????????????

Agilodocodon

000001100001000011000?000000111001002000 11000??00?0000000?01000110
111112010?0??00??0?????1010?0????0000???201031100?0000???00?00?????0????
?0??11????0??????????????????03320121??110?0?00???????000100100000001011 0
002001100000011000???????????0020????1010?0000??????111001????0????0?00?0
10?0011101000010210000000000000?00000?1011000?0100?01010101000000000000
000000100000011010010001????102001 1???0?231100?????0000??010?12????????

1??21011100??00000???1001100100000?0?21???1110010000?2??1???0???????100
0???????????002???0??????01000???????????????????110????0????20??01201??10
??00?01100111?100????11??????????????????????

Microdocodon

000001100001100011?0??0100001110010020001100??00?000000??12000110
111112010?0??00??0?????1010?0????0000???201031100?0000???00?00?????0????
?0??11????0??????????????????03320121??110?0?00???????000?00100??????0110
02200???0?????00?????????????0020???01010?00000000001111 0011?10000000?0010
1100011100??????2100000?001000000000?1011001101 00??10101?110000000000
0??0?1?00000?000?0??00[0
1]??001?2??11?????23110????????00??????????????????????????????00???????????
????0??????1???1110010000????1????0???????0?????????????0?2?????1??????????
???????????????????110????0???420??01201??1???0??01100111?100????11????0?
???101100?0?00??

Adalatherium

1111?10?????2301?1?121101000111014011000310 1???0?????????????????????
?????0?0???1??????0?1115?????????????0???0??00?1???????????????????????????
?????????????????????0333?0????23????????????????3104111101?01??333243???
001?1?112??????????10020???0101101101 1???1?1112??1?010?2001000011102210
1011000101 1?1100000?1011101110111100101010110111112112120020111010111
000002110000[0
1]0101?1??1010?10?0?0???????31?11??1????????????????0?????????????00???????
???????????????????????????????????????01?110000??0?0????????0120????????10
0011?1???1?????????????110??????014?0??????0??11?2000111011120101 1?11?11
?1????????????????????

Vintana

???????????????????????????????????????3??????????????????????????????
???0??????????0?1114??
??????????????????0??3?0????2?????????????????3???????1??????3??24???????????
????????????2102???
???1100
1010110???0123111131111101000110 1?130001?0101211111111000000100200?1
0211???????21?????1????????0000???201110000000010?1111?1?01200200000110 0

0?0?000111110221????????10?????????????????0??11????????????????11?11?11?1
1??????????????????

Eleutherodon

??2???1????0??????????????020??????
???11221????????1113112112 0??????0???0??01?0?????????????????????????????
???????????????????0333011?1113?????????????????1??????????????????????0????
????0?1110??01?103???
??
??
??
?????????????111210201???01?????????1?1???????????????????????????????????
????????

Sineleutherus

??2???111?1???????21????2?020??0??
??1????21????????111311211[0
2]0??????0???0??01?0??033?0?
??1113?????????????????11??????????????????????????????0?1110??01?103??????
??
??
??
???11?????01???
01?????????1??

Millsodon

???
??001021???????????????10100???
??????????????????03?3??????13??
??0?11??0????103??
??
??
??
?????????????2?0?0????01?????????1??
????

Thomasia

??0????????0?????00?0?00???
01000021????????11131110100??????0???0??0??????????????????????????????
??????????????????0333011?1?13??????????????????1?000?????????33?????0?3?
??11?10?110?001??03??
??
??
??
?????????????111210001???010[1
2]???????1??

Haramiyavia

0?000?0?000000000??0??000000111000000000000100?????????????0??0?00000?
00??200000021????????1113?010100??????0???0??0120????????????????????????
????????????????????????0333011???13???????????????1001000000100010?133?1
???0000??1121001100000?00???
??
??
???0???????????????????????????
?????????????????????111210201???0102???????1?000????????????????????????
?????????????????

Kalaallitkigun

0?0001??0000000000?0??0000?????????000001?0????????????????????00???
?????????21????????1113?0101?0??????0???0??0??0000????0?111????0??????????
???????????????????????3???????13???????????????????????????????13??1??????
??????0??22???10?012??
??
??
??
???????????????1?1?????1???2102???????????0???????????????????????????????
??????????

Vilevolodon

1111?11200002301?1?1101120?32111240010002101111110?????021??2?30010
?00????1111121????????111311201[0

1]0??????0???0??01?2??044401
1?1113?????????????????41140000 1???1??334443???0?30111120?11100001?103011
11001??1000100111110000111100111 00[0
1]001110000110011100121002 01?000001?00110?1000000?001?00000?0000001200
0?000?0001101000101001 00??011?00101??10???0?2?1?????????????????????????
??????????????????????10???????2211021?1???011??11000?2???????01?301010??
001????????0121???021??001101??????1??0[1
2]?1???????11121000111401 01???????1?120110?00011??1??1???????0??0????101?
???0?00??
Maiopatagium_furculiferum
??000100?????02???????????????
????00021????????1113???
??????????????????0??3011??013????????????????41???0001??????3??44???????2?
11?????0???00???10?01?110010?100010011111000000110011100[0
1]00110?00011000110012100201?000000?00000?1000000?000?00000?0?0000??00
0?00??00000?000000100100?10????0???
???20?012??0013010?0??011
????????0?2?????2???00000??000????????????????1121000?0?4010????????1??2?
110100011101???????1??0??0?????????????????
Cryoharamiya
??
???010??
?????????????????0??3??????13???
???0???2????103??
??
??
??
????????????2?0?0????010????????1???
???
Cifelliodon
???????????????????????????11??
??00??
?????????????????0??3???????1?????????????????31??????01?0???????4?????2?????

????????2?0???0??
??0000
101?111?0?0?231100???????????010?11?0000??01???11?????1000000001?00100?
?????????0??????????????00000???0013000100001011????0?0221???0001002?101
1000000??1121????????11????0????0?0?????????1??????????????????????????????
????????????????
Arboroharamiya_jenkinsi
1111?11200001301?1?1101120?311112400100021011111110????1?21??2?30010
?00????1110221????????1113112120??????0???0??01?2????????????????????????
?????????????????????????0333011?1113????????????????411400001???1??33444
3???0?30111120?111000011103?????001??10?????????1???????????????????????
?100?????121??00???00??1?00????100?000?00????????????0??????????????????0?
???001?0??01???
????????????2?1??2???3100????1100??
??????????????????????1101112100001?140101???????1?120110100011?0101141?
111??1???????????????????
Arboroharamiya_allinhopsoni
1111?11200001301?1?1101120?311112400100021011111110????1021??2?3001
0?00????1111021????????1113112110??????????0??01?2??????????????????????
??????????????????????????0333011?1113????????????????411400001???1??3344
43???0?30111120?11100001?103?1111001??100?????1?11000???????111??[0
1]??1110??0?100100[0 1]11210?001?00???1?00?10??00?000?00[1
2]00???????00??120?????0[1 2]1101?[0
1]?00010100100??0??1?01???110???01231112 3????2?????????1????????????????11
102?10?0????100?110??221102?21300011??110000???????????????10????????????
?????????2???0???????????1??????????1101112100011140101???????1?120110100
011?0101141?111??1???????????????????
Xianshou_linglong
1111?11200001301?1?1101120?311112400100021011111110????1021??2?3001
0?00????1111021????????11131120100??????0???0??01?0????????????????????????
??????????????????????????0444011?1113????????????????311400001???1??3344
43???0030111120?111000011103?1111001??100????????????????????????[0
1]001110000?1001110012100201?0000?1?0011???00?000?001?????0?0?000?120??

???01??011[0
1]1000?0100100??01?1???????10???0???????3?????????????????????????????????
???????????????????????????21?????????????????????????????0????????????????
????????0????????????????????????111210001 1?40101???????1?12011010001110
1011?1?111?????????????????????
Xianshou_songae
1111?1120000?301?1?1101120?31111240010002101111110????1021??2?30010
?00????1111021????????1113112010 0??????0???0??01?0???????????????????????
?????????????????????????0444011?1113?????????????????1?400??1???1??334443
???0??0111120?111000011103?1111001??1??01???1111??????????111??[0
1]0011100001100????012100201??0?001????10?100?000?001?0???0?0?000?120???
??01??0??[0
1]1000101001 00?101??
???????????????????????????1??
???????0?????????????????????????1112100011?40101???????1?120110100011101
011?1?111????0?????????????????
Shenshou
1111?11200001301?1?1101120?31111240010002101101??????????1??2?20020
?00????1000021????????1113111110 0??????0???0??01?0???????????????????????
?????????????????????????0333011?1113????????????????411400001???1??33444
3???0?30111120?011000010103?11110011010001001111100001 10?0011100[0
1]00111000011001110012?00201?00?001?00110?100?000?001?0???0?0?000?120??
???01??011[0
1]1000101001 00?10111??101?110???01231??23??????????????1?????????????????
????00?0????1????????????????21??????????????20??????0??????10??00???????????
?????02?0?0011?1??????1?????????????1112100010?40101???????1?020110100011
101011?1?1????1?0?????????????????
Qishou 1111?11200001301?1?1101120?31111240010002101100000????102[0
1]??2?30020?00????1000021????????1113111110 0??????0???0??01?0??????????????
?????????????????????????????????????0333011?1113????????????????411400001?
??1??334443???0?30111120?11100001?103?1111001101000100 11?1100001?0?001
1100[0
1]00111000011001110012?002?1?00?001?00110?10??000?001?????0?0?000?120??

???01??011[0
1]100010100100?10111??101?110???012311123????2?????????1????????????????
?????00?0????10???????22110??21???0?1????????20??????0??????10??00?????????
????????????0????1??????1?????????????111210001014010З???????1?0201101000
11101011?11111??1??????????????????

Rugosodon

1111?10???00?301???10?2120101111240010002101100 01??????030??2?00001
0?0????1?10121????????11130111000??????0???0??00?0???????????????????????
??????????????????????????033300??0023????????????????2014000001101??01442
2000103?1111211010101 00?0021111 10?1111100000110111100111100111001 00?0
0?0010101000010 21?011000000?1?00100?20110010001210010?00100012001 0001
100010110001110000111 01?0??
???????????????????????????????????1???
???????????????????????????????????????1111101010?40102???????11010110100
011001??1???????????????101100?0?00??

Kuehneodon 1111?10???001301?1?10?21201011112400100021011000 11????[0
1]030??2?00001020??311?10021????????11130101000??????0???0??00?0?????????
??033300??0023????????????????211400
0001101??11443200010 3?11112110001010 0?0021??????????????????????????????
??
??
??
??111110?010??010????????110???????????
???????????????????????????????????

Jeholbaatar

1111?10???002301?1?10?21201011112400100021011000 11????1030??2?00000
?10??201?10121????????11130101001??????0???0??00?0???????????????????????
??????????????????????????033300??0023????????????????21140000 1???1??02442
30001030111120?00001112?002????1001111100000110111 10?11110?111??100200
?0010101000??0210??100000???????????01100100012100 10?0010001200100 01100
0?01100011100001110100001010110?0?00??1?11???????????????????10??????????
???1?10??0?????1?0???1??22110???1???10???211?0??????1??11?10??101?011?????

???1220???0110?101?010010?????????????10?1111111110140101???????11010?10
100011?111014111????1???????????????????
Plagiaulacids 1111?10???00[1
2]301?1?10?2120101111240010002101100011????1030??2?00001020??211[0
1]10121????????111301[0
1]1001??????0???0??00?0??033
300??0023?????????????????211400001???1??0244[2
3]30001030111120?00001112000 2???
???????????0?????????????????1??
?????0???????????????????????231111301??0000??01001211101100211???1111?10?
00000001000101111???????21?0???????????0?000201001002000010??00001101??112
2002000100[0
1]011010000?21??????????????1111111110?40101???????11010?10100011?1??01?1
11?????????????????????????
Cimolodontans 1111?10???002301?1?10?2120101111[1 2]40[0 1][1
2]00021011000[0 1]0????1030??2?00000?00??20[0 1][0 1][0 1]0[1
2]21????????111301[0 1]10[1 2][1
3]??????0???0??00?0??033300?
?0023?????????????????3[0 1]04[0 1]0001???1??1[2
3]44330001200111120?0000111220020111100111110000011011110011110011100
100[1 2]00?00[0
1]0101000??02101110000000111?100?201100100012100 10?00221112001001110 01
101100011100001110100001010[0 1]10?0?0023111130100[1 2]00[0
1]00010012111011002 1121[0 1]111[0 1]010[0 1][0 1]0000001[0
1]001011022?1?2?21100111??211100000[1 2]01001002000101?000011[0
1]1?111220020011001 1?10100000211102 23???11?11111[1 2][1 2][1 2]10?4010[2
3]???????11[0 2][1 2]0[0 1]1[0 1]1[0 1]0[0 1]110111[0
1]1411111 1?1??????1?1?????????1?
Hadrocodium
0??0?1120010?001?1?00?000000110?0100200?01000??00?000010101000011 00
?00?0??0????00??0?????1011??????0?20????00000000?0000??00?00?????0?????0
????????0????????0?????????00000100??110?0?00???????0000000000000001000334
443000000011001???????????002???

??0??
??????????10000020101?0?0023111230100000??010?111010?1001102101011010
00000000100?10010???????21?0??????1????02012?10301010001000000111?1??10
02001012110001?00??000?2??1213???????110????0????20??00?00?????00????????
?????01????1??????????????????????

Shuotherium

0010110???000001?1?00?0?00?0??????0?000101000??????????00111110000?
11??0?0????121?11100?1011??????00100??103231110?01110000100?????0?????1
0?1010?20000?0??0000000??10011200????22101000??0?100111?????????????????
3??????0???????????????????002???
???
???
???
???????????????????????1??????0????20?????00??1????????????????????????????
?????????????????

Pseudotribos

0?100110000?00001??00???????1110010100011000???0??0???000111110000
??1110?0????121?11100?1011??????00100??103231110?0111000?1000?????0?????
10?1010?20000?0??0000000??10011200????22101000??0?100111??01?0??0100??0
0033100000??011000???????????002?????101??00?0010001???????????0?????0???
12100000?0??????211???00000?10?00000?00?00?????????????????????????????????
?????????????0????0???
???0???????????????2??
??1??????????????????????????????113????0???420??11200??1??????????????????
???????????????????????????

Asfaltomylos

0011111111000001?1?00?2200001110310120010??00?????????????11011?0???
??????0????121?111???1012??????0??112?002100?00??1110221131100000101112?
??????????????????????????011?0?????22101?101011??0000??????????????????
????0????????????????????0?2??
???
???
???

?????????????????????1??????0???420??1220000????0???????????????????????????
????????????????

Ambondro

?????1????00?????1????????0???????0???0?0??00?????????????12131?0???110
???0????121?111???1012??????00?1123000121000??11102221311020??101112????
????????????????????????011?0?????22101?101011010000???????????????????????
?0???????????????????0?2???
???
???
???
??????????????????1??????0????20??1220001????0??????????????????????????????
?????????????

Ausktribosphenos

0010011101000001?1?00?22000011???101200101000?????????????121310000
?11011?0????121?111???1012??????0021123000121000?0221022113111200012212
3????????????????????????????033?0?????22111?101111111000?????????????????3
?????00???????????????????002??
???
???
???
???????????????????????1??????0????20??1220011????0?????????????????????????
??????????????????

Bishops

01?101110100?001?1?00?2200001110310120010?000?????????????121310000
??1011?0????121?111???1012??????0021123000121000?1221022113111200012212
3????????????????????????????033?0?????22111?101111111000?????????????????0
3??0??00???????????????????002???
???
???
???
????????????????????????1??????0???420??1220011????0????????????????????????
???????????????????

Teinolophos

1100111101002301?1000?22000?1110310120010??00???0?0????????0??0000?? ?0????0????121?111???1012??????011110200012 0?10??2220422121111 0??0?2?23? ????????????????????????????3?0?????22111?111112102000???????????010?11? ?0??10??0??00[1 2]???????????0?2?? ??? ??? ??? ??????????1??????0???420??1220011????0???????????????????????????????????? ?????

Steropodon

011001????0??00??1?????????????????????10??????????????????????0??????? ??0????121?111???1012??????0111102000121010?0222042212111 10??0?2?23????? ??????????????????????033?0?????22111?111112102000????????????????3??3??? ????????????????????0?2?? ??? ??? ??? ?????????????????1??????0????20??1220011????0????????????????????????????? ????????????

Obdurodon

11?1?11101002301?1?110220001111031112111010?0??0?????????1013100?? ?00????0????121?111???1012??????0111102000120?10?0222042212111 10??0?2?2 311?1??????????????0????????033300????22111?1111131020005??5????1???1??3 33443???0????1??????????????002??? ????????????21???1????11??? ???????????1000021101 10?0?0023 1111?????0000??010?111?110100111210 1110?1 00000000000?00??0?????????1?0???????????03012?10000?40?1101?11?11101?011 22?000121100011?0??00002????2????????113????0???420??1221 0110???0???????? ?????11???????????????????????????

Ornithorhynchus 11?1?10?01002301?1?110220001111031112111[0 2]1000???????????????????????????????????21??11???10????????01?1???????20?1

0?022204221211110????2?2311?1???????????????????????033300???????????11?1
??1?2???5??5????1???1??334443???0????1?????????????0???1101111000000100
0100000101000000000000012100000?010001021111100001110000000112011002100
000?0000?00010110000000000001100001101000?110[0
1]0100002110010?0?0023111141100000 0??0100111011010011121011101100000 0
00000?00??0?2211021211001 11?12101003011010000240011 01?11211101?011221
00012110001 1?0?10000211122 3000?10???3????0???4???????1???0????1101100110
??1114?1?11????0000?11111100?1101
Tachyglossus
11?1?10???002401?1?00?0000001110 3111??1?01000????????????????????????
???
???5??5????1???2??334443??????
????????????????????1110111110000001000100000101000000000000012100000?0
1000102111110100111000000112011002100000?0000?000101100000000000000 11000
0110100?110[0
1]0100002110010?0?0023111141100000 0??0100111011010011121011101100000 0
00010?00??0?2211021211001 11?1210100301211020024001 1?1?11211101?1112210
0012110001 1?0?[1
2]0010211122 3000?10????????????4????????????????1101100110??1114?1?11????
0000?11111100?1101
Fruitafossor
11?0011300002001?1?1100000111110010110 01?1000???0?0000000?10?00100
0?00?0??0???????????????100????????0??????0???00?00?0??????????????????????0?
?0???????????????????????055400????0????????????????????02?0??010001022333
20000?0011000???????????3010??????010?001???????0?00001 1000?0001000001210
0010?010001021??????0000???00??0?100?100?0?0?????????00000000000?00000?
??????????0010??0????10???11???
??0??????????????2?1??
????????????????????????????????1??????0???420??????????0??0????121111???10??
???????????????????????????
Gobiconodon 11?0010???00[1
2]101?1?1112100111110140010010100 0??00?0000001?100101100?10000?0????00
??0?0???1021??????0010???100100?0100?00???00?00?????0?????0??100???01????

???0?????????000100????120?1?00???????0002?030000010001011120000000000101
10??????????2002????0101100000000?1011211111110011110000010?10000?00000
?0210021000000000000000?100?000?0000000?0?000000100000000000000???????
???00?1000000102001 1?0?002311113010000 00??010?121120000011110011100200
000000011??10011????????1?1????12????002002110000110000000000111100011 12
210210110000 1?000001??0??0?00???????110????0???420??10000??0??00?10100??
?1?1121?1111110??1?????????????????
Repenomamus

11?0010???002101?1?11121001111101400100101010??00?0000001?11010110
0?10000?0????00??0?0???1011??????0010???100100?0100?00????0?00?????0?????
0??100???01???????0?????????000100????110?1?00???????000200300000 1000101
1220000000000101 10???????????0020000010110000000001101121111111001111000
0010?10000?00????02100210000000000000000?1000000?000000000?0000001000000
00000000?0?0??0??0?0??1000000102001 1?0?002311113010000 00??010?12112000
0011110011100200000?000011??1001????????21?1????12?????02002110000110000
000001111000111221021011000 01?000001010??0100???????110????0???420??100
00??0??00110110011 1?1121?1111110??1????10110??0?0???
Amphilestes

11?01?0???002101???1112??0??1110140020 0101?00??00?????????11010?100?
00000?0????00??0?0???1011??????0010???100100?0100?00??00?00?????0?????0?
?????????????????????????000?0?????120?1?00???????000???????????000?21??0
0000???????0??????????002???
??????????????????????0???
??
???1?????????????????????????????
????????????????????110????0???420??10000??0??0??????????????1???????????
???????????????
Yanoconodon

11?0010???002101?1?11121001?11211400100001000??00?0000001?00000110
0?00000?0????00??0?0???1010??????0010???002000?0121?00????0?00?????0?????
0??000???01???????0?????????00000121??110?0?00???????00030030000000000003
33343000010011110???????????0020000010100000000001101121111111001111000
0010?10100?00000102100210000000000000000?1000000?000000000?000000100000

000000000?0?0?00??0?00?00000001020?11???00???????????????????????????0??
????????????????????????????????????21???110??210100?00?????0??????11?????
?11?????122??????????????0???????????????????110????0???420??10000??1??001
10100011??1121?11111?????????10110??0?0???

Liaoconodon 11?0010???002101?1?111210014111014001000010[0
1]0??00?0000101?000000000?0010000????00??0?0???1010??????0010???002110?0
[0
1]?1?00????0?00?????0?????0??100???21?????????????????000000????1?0?0??????
????000200200000[0 1]010003323420000[0
1]001?000???????????002?1?111010?000?????1?1121111?11001???000?01??10100
?0000010210022000000?0000000?1100000?0000?0000???00?01?0??00?00??0?0?0?
000?1?0000?0000?0010200111100[1 2]23111[1
2]3??00200???????1?????????????????1110??00000???????100??2?1112121???10?12
22010010??????01?1???11??001?11?????12?????111???1?110?00?????????????????
110????0??0420??10000??1??001101000111?11214111111????????????????????

Jeholodens

11?0010???00?101?1?111??001?11111400100001000??00?0000001?010101100
?00000?0????00??0?0???1010??????0010???002000?0121?00???00?00?????0?????0
??000???01???????0?????????00000121??110?0?00???????00010010000010001033
2232000000011111????????????00200000101110000000011011211111100111100??
010?10100?000001021002100000000000000?1000000?000000000?0000001000000
000000000000000100000?0000000102?011?0?002311?130100??0????100??1??????
?????????????0??0?0???????????????????????????1221?????????????00?10??11????0
0???????122????011??001??0??????1??????????????110????0???420??10000??0??00
?10100011??11?1?111?1????1????10?????0?????

Spinolestes

11?0010???112101?1?11121001111101401?0?101000??00?000000??00000100?
?????0?0????00??0?0?????11??????0010???000000?013??00???00?00?????0?????0?
?100???11???????0??????????000000????110?1?00???????000200200000100010212
2320000000010110????????????002??1101010?0010?00110112111111100111101000
1101000100??????21002100001001?111110110000000000?0000?001000100000000
0??0???????0?1????0100?10???1120111?0?00231111?????0000??010?12?1??????11?
11111?1020000?0000111110011???????21?1????12??????2??2???0???????00??????

12??????22????????????????????????????????????110????0???420??10000??1??00?1
0100011??11?1?111????????????????????????
Jueconodon 11?0010???002101?1?11121001411101400100001000??00?000?00[0
1]?0?1000000?0010000????00????????1011??????0010???101000?00?2?00???00?0
0?????0?????0???00???11?????????????????000000????110????????????000300200
0000000103323420000000010000???????????002?1?11101??00??????11112121??11
0?1111000001??1[0
1]0010?000010210021??00?0?0?00000?1100000?0??????00?000000???0????00??0?
00??0??0000000??000???1?????????0?23?1[2
3]?1?1??2??1???????2??
????20?2????0??????11??001???????????????1211?0??1???00?????????????????110
????0???420??10000??1??001101200110?1121?111111?????????????????????
Trioracodon
11?00?0???002101?1?11121001011111400100001000??00?00000?1?010101100
?00100?0????00??0?0???1010??????0110???10[0
2]000?0121?00???00?00?????0?????0??100????1???????1?????????00000112??110?
0?00???????000??0300??000000012332100000??011000???????????002????????????
???
???0???????????????02311?????????0??
?0??0??1010???0???????????????0??0???1??100????????????0???????????0???????
0????????1???????????????????0??????????????????????????????110????0????20?
?10000??0??00???
Priacodon
11?0010???002101?1?11121001011111400100001000?????0000001?000101100
?10100?0????00??0?0???1010??????0110???102000?0121?00???00?00?????0?????0
??100???01???????1?????????00000112??110?0?00???????000???300??00?0000122
2210000??011001??????????1002???????????????0??????????????????????????????
???
??????????0?????0????1???00231111301??0000??01001?13100??0111100??100??0
00??0??11001001????????2??0???????????020?????000??????1?????????[0
1]1??122?110???????????0??0??1?????????????110????0???420??10000??0??00???
???????????????????????????????????????

Volaticotherium 1111?1101000?101?1?1????000?1110??0010010[0
1]010??00????100?020000000?000?000????00????????11????????0???????1?000?
00??000000????1???????????
??????2013000001000001143220000[0
1]00??000????????????002?????????????????????0??0??????????????0?[0
1]???01010?00????0??????????????0?01010?0000100?0?1?????????000????????????
???????0????00001 1001???
??
??????????????????????????????????????110????0???420??00000??1??00??000001 1??
??????????????????????????????????

Tinodon 1??01??????0[0
1]101?1?1111?00????1004?130?10??00?????????????01010?000?00?00?0????12001
00???1011??????0?100?0100100?0100?1100001000?????0?0??010?101????????0??
?0????0??0002210?????22101?00???????000?????????????01??22??1???0??????????
?????????0?2??
??
??
??
??????1??????0????20??11100??0???0???
?

Akidolestes

1????10???00?20????111210010111014011001 11000??00?00000?1?110100000
?00120?1????1210111010101 1??????00100?0100000?013012200?01000?????0?0??
011?101???000??0??0000000??0002220121??22111000??0?00000010 0100??010001
100010000000001 1110????????????002?????0010?00001001101121111111100111101
11110?111010000001?21111201000011?00000?21001021001000000?00100201001
00011000010000121000?0111010?001020??1?????????????????????????????????????
??0???????10??0???
11??????12??????????0????0?????????????????????113????0???420??11100?????00?1
0100111???1?1????11????1?????????????????

Spalacotherium

1110010???001201??????2100101110140110011 1000??00?00000?1?110100000
?00100?1????121111100?1011??????00100?0100000?013011200?01000?????0?0??

011?101???000??0??0000000??0002220121??22111000??0?000000?0?????????010
?000?00000000??110???????????002?????????2????????????????????????????????
?????????????????????????????1???????0????????????????????????????????????
??
???0?????????????????????
????????????????????????????113????0???420??11[1
2]00?????00??
Zhangheotherium
11?0010???001201?0?11121001?111014010101110000??00?0000011?11010000
0?01?00?0????121110000?1011??????00100?0100000?013011100?01000?????0?0?
?011?101???000??0??0000000??0002220121??22111000??0?000000200200??01000
10220000001000011110??????????2002?11000011211001001101121111111001111
0111110?11101000000102111220000001?11100?2100100?001000000?001302010
0100011000010000020000001 1101????1020111?0?012311112????110100010?1?1?
10???0??1210111102000000001?0?101?1???????21??????1??????0??????0??1????
?10????0????22???12???2?????????00???????????????????113????0???420??11100
?????0011010011 11??121???11????1????10?????0?????
Origolestes
11?0110???002201?1011121001111101411010111000??00?1000011?110[0
1]00000?0111000????121110000?1011??????00100?0000000?0[0 1]3011[0
1]00?01000???????0??0111101???000?????100?000??000[1 2][1 2]10121??1[1
2]10?010??0?0?0000300300000101010 2212[1 2]0000000011[0 1][0
1]0???????????002?1?10001121 1010??11?10101?111100[1
2]1110010110?11101000000102111220000000?1011100?1110000?0011[0
1]1020?0013021[1 2]1[0
1]11101100000010002000000110011?001?2?1110100?2311112000111????010?1?1
1????0??1?????1110??00000000?1??111??0?211??21310001021 2000?110?1?0?011
01001001001110?????0121??21210?0011011001111001[1
2]2?????111113????0??0420??11[1
2]00??0??0011010011 11??1214???11?0???????????????????
Maotherium
11?0010???001201?0?1112100101110140101011100 0??00?0000011?11010000
0?01?00?0????121110000?1011??????00100?0100000?013011100?01000?????0?0?

?011?101???000??0??0000000??0002220121??2221000??0?000000200200??01000
10220000001000011110???????????002?1?00001??1100?0011011211?11110011110
111110?11101000000102111220000001?1?100?21[0
1]?100?001000000?0?13020100????1100001000002[0
1]00000111010?001020111?0?012311112????110100010?1???10?100?11000111??2
0000000001???101?????????????????12?????0201??20?0??22??1000000???12???1
2?002?1???0???00??????1?????????????113????0???420??11200??1??00110100111
???1?1????1??0??1????101100?0?00??

Dryolestes

11?0011010001201?0?110210?0111101301200111000??00?0000011?00000110
0?01?00?0????121010000?1012??????00200?0002000?00?011100001120???000?0?
?110?001???000??0??0001000??0002220121??22101000?00?100000100100??00000
001100000000030111110??????????2002?????????????????????????????????????
???
????????????????1???
??1???10?0???????????????????
?????????00??0?????????????????113????0???420??12200??1??00??0?000???????1?
???????????????????????????

Henkelotherium

11?0011010001201?1?1102101??11101301200111000??00?00000?1?00000110
0??0?00?0????121010000?1012??????00200?0002?00?00?011100001120???000?0?
?110?001???000??0??0001000??0002220121??22101000?00?100000??0????000?0
0?1100000000??0????0????????????0?2??????010?110??0???01121111?110?111101
1?100?111011100001?21?02200000000111000 0?2110000?10??????????1???????????
??????????????0????0??1011???1020?11?0?0?23111?3???????????????????????????
????????0??1?????20001????1??????
?????20???????????????????????????????113????0???420??12200??1??00??0100
1112??121???????????????????????????

Amphitherium

11?0111010001201?1?110210?0111100301200111000??00?11?0?0??00000????
?01?00?0????121010000?1012??????0000100002000?00?001100001110??0000?0??
1101001???000??0??0001000??00022101????22101110000?000000???100??????00
??000?0??00????????????????????0?2???

???
???
???
????????????????????????????113????0???420??1[1
2]200??1??0??

Peramus

11?0111010001201?1?1102101011110030120011100??0??1100201?00020110
0?00?00?0????121010000?1012??????000100000100?0110011[0
1]0001111000000?[0
1]?01111201???010000??00010011000011110121??22101110000?000000???2??????
00000003310??00??0????0???????????0?2??????????????????????????????????????
???
???
???
?????????????????????????????????113????0???420??1[1
2]100??1??0??

Vincelestes 11?1?1101000[1
2]201?1?1101101011110130120011100??00?0000101?100000000?00?00?0???12
1010000?1012??????00000?0002000?00?001100001110???000?0?0111?101???0100
00??000101110000111100?1??22101000?00?000000100300000000100033334200000
30??000???????????0?2011110010?11012?1?101?2110111100111010100?1110??
000001?21012200000001110000?2110000?1020?00?10001302010110001101000??0?
??210???0??00111002020011?0?0123?112400?11101000100111010010011[0
1]21111110201100000011001111??????21?0???????????0101101000102010 0000
010111000010020020001001011000000??1??????????1??113????0???420??11100?
?1??0001010?0?????1?1????11?0????????????????????

Nanolestes

11?0011010001201?1?1102?0??1111003012001110000?????????????00000?100?
00?00?0????121010000?1012??????000010000210 0?011001100001110?00??0?0?01
11?101???0?0000??101201110000111 0121??22101110000?00000010?1???????0001
?02??0??00??0????0???????????002??
???
???

???
????????????????????????????113????0???420??12200?????0?????????????????????
??????????????????????

Kielantherium

11?0011010?012?1?1?1101101?2??????0130011?000??00?????????00000?100?
00?00?0????121010010?1012??????0000101002110?0110111020114110000000?[0
1]00111?11?0?00100?0??10020110101011?0121??2211111000100000000?????????
??00??12??0???0???????0???????????002??????????????????????????????????????
???
???
???
???????????????????????????????113????0????20??1220000????0????????????????
???????????????????????????

Aegialodon

???
??????1??0100???1012??????0000101002110?011?1110201141 1000??101001??????
??????????????????????011???21??22111?1000100000000?????????????????????????
????????????????????002??
???
???
???
????????????????1??????0????20??1??0000?????????????????????????????????????
???????????

Montanalestes

11?1?11210001401?1?11012010311100301300111000?????????????12020?100
?01000?0????121?111???1012??????0010111002110?0112012021114110001110100
2?????????????????????????????0???0?21??22111?100010110000???????????????0
3??0??00???????????????????0?2??
???
???
???
??????????1?????????????1??????0???420??12200001??00??????????????????????
????????????????????

Prokennalestes 11?111121000110 1?1?11012010211100301200111000??00?1[0
1]112?1?120200100?00020?0????121011120?1012??????0020101001110?01120120
2111411000111010021102 1?010010011001011010?0000112012 1??2211121000101
10000?????????010010033?00000?00?1??0??????????002????????????????????
??
???231112511111101[0
1]001002?10?????13?1?111011???11001???110?111?????????2??1???????????????
?????0?????????????1?????????????1?????????????0?????????????????1?3????0???4
20??12200001??00???

Murtoilestes

???00??????
??????121011120?1012??????0?20111001110?011?012021114110 00??10100?11021
?0100?0011001011000?0000112?121??22111210 0010?10000??????????0?????????
??????????????????????002??
???
???
???
????????????????????1?3????0????20??12200??1??????????????????????????????
???????????????

Eomaia

11?1?11210001101?1?11012010211100301200111000??00?1011111?12010010
0?00020?0????121011120?1012??????0020??1001110?0112??202?1141100011101?
?211021?0100100110010??000??0001120121??2211121000101??000000100000000
00000331000100 00?1000???????????00211?1100102110120 0?1011221011110 0211
10111200?2210111000010211122000000 01?10110?2111000?01221000111223 0201
0130111101000001002100001110 0111?0?1????2???0????1??51?11?????????????1??
??????????????????????????11?????????????????????????????0????????00??0??10?
?0???11????10?20?????????01???1??????1?????????????113????0???420??1?200001
??00?10110111???1?1???????????????10110100?001?

Juramaia

1?1???101000??????????12010211??0301100111000??00?1011201?120100120?
0?020?0????121011130?1012??????002???????11??0112?1??2?1141100011?????21
1121?0100100110010110110101120121??2211121000 10?1000000010?0?000100

00033100000000?1000???????????0021111001??1?????????112210??110?211?01?
???0?22101100????0??
????????????0??
??
??????????????????????????????????113????0???420??12200001??00?101???1??????
????????????????????????????

Sinodelphys

11???1121010??01?1?110110??311100301?00111000???0?10??211??10100100
??0?1000???1210111???1012???????000??1000110?0113111?2111421110001111??
1??121?????????????1?[0
2]111000?011?0121??22111?10001001000010010?0000000000133110020?10?1000
??????????002?????001021?0120??10112210111002??111112?0???1010?1111002
1????00??0001?11??0??10?000?01211010???125120201 2?21?21000110110210000 0
110011??
??????????????????????????????????00?????10??????11???????2?????????0?????
??????1?????????????113????0???420??12200001??00?1011??11?????1???????????
?????????????????

Acristatherium

???1????????????????????????11?????120011??00??00?1???201?????????????
????????????????????1??????????????????????11????????14210??????????11??????
?????????????????????0?120121??2211121000 10???000???????0000????0??31??????
???????????????????002??
??
??
???0????????????????????????????????????
???????????????11?????0???420??12200001??00??????????????1?11??????????????
???????????

Ambolestes

111111101010?101?1?110110102111003012001110 00??00?1000211?11010010
0?0001000????121011120?1012???????0001010001 10?011211102111421110001 11
10111121?000110010??1112111000101120121??2211121000100 10000??0?????000
1???0133111110????1??1????????????002?1?110010211 01201?101121101111002 11
10111210?111?1001110002111220000 00?1?11100?01110011112??000111?2412010

1???1011100?00?0021?000111001????3?2?112???0?23?112??????1??????????????
?????????????0??0???1?????????????????????????????321?0?21??????00?????10???
???11?????022?????1?1?0????????????1?????????????113????0??1420??1220001??
00?1011??11????????????????????10110100?001?

Cokotherium

11111110100014 01?1?1101100031110030120011 1000??00?1110201?11010010
0?0001000????12101112111012??????0020101000?10?0113112021114210001 1101
00211121?0000100110010120 00??0001120121??2211121000101100001002000000
00000003310000 0000?1000????????????002???1100???1101??????112?1011110?211
??111?0??1???100000000??????????????????????????0??????????????????????????
??????????????????11?01??002?201121100?2311125??1121?11211[0
1]????0??????3?????????0?0000??1??11001 1110????????1???????2321?0??1???2??
00?1?0?10??010?11?????022????01?1?01??010?0?????????????????11?????0??1420
??1220001??00??0110?1?????21?11??1??1??????10110100?001?

Microtherulum

1111??1010002??1?1??101100021110000120011 1000??00?1111201?11010010
0?0001000????121011120?0012??????0020101000110?0111112021114210000 0111
01111121?0011100110010120110001011201 21??221112100010110000000 1000000
00000003310000 0000?1000????????????002?1???0?1???100?????????????????????
?????????????????????211?22000000?1?10??0??101000?01??????????2???????????
??????10000021?00011?001????2?2??12??????31112?????2??????????????????????
??????????0?0?????1???1?11?3120?11?13201????321?0?????????????????0??010??
??????022?????1???00?1010????????????????101113????0??1420??1220001??00?
????????111?2?411??1??1???????????????????

Kennalestes

11?1?1121000140 1?1?1101200021 1????0?200111000??00?1111201?100200120
?00100?0????121011120?1012??????0020111002110?0112222031114110001 11010
0211021?0010100110110 10000??0001120121??221112100010110000100 2????0001
0011[0
1]3320001000011001???????????00201???
??2?????????????????
??????????????1100212211221002231112511??21011010?02[3
4]1?2001013?????1011020100011001100111 10???????21?2??????321300?0110200

000200??0??01011111?11002002201110 01?1010?00??1011223??????113????0???
420??1220000 1??00??
Asioryctes

11?1?11210001401?1?1101200021110030130 0111000??00?1112201?11020012
0?00010?0????121011120?1012??????00201110021 10?0112012031114110001110 1
00211021?0010100110010 00000??000112012 1??2211121000101100000001000000
01001113321001 0000?1001????????????00201111??10?110??????????????????????
0?????0????0??110000??????2?0??00???????0???1?000?01221000111223020 10130
1111010000 00?020000?1??0011100212211221002231 1125?1??21011010??241?200
1013???1110110201000110011001 1110???????21?2??????32130020110200000200?
1011010?1111?110020022011 1001?1010000??1011223???????113????0???420??12
200001??00??????0??????2?????11??????????????????????
Ukhaatherium

?1????????00?40???????????0211????0??00?11000??00?1112201?11020012 0?0
0020?0????121011120?1012??????0020??1002110?0112012031114110001110 1?02
11021?0010100110010 00000??000112012 1??221112100010110000000100000000 0
001133210010030?1001????????????002?1??1?010?110??0???0?1221011110?21110
111200?2210111000?1?210?2200000001?10110?2111000?0122100011122302010 1
30111101000 00???200???1???01??00?1?2??2210???3?????????2??110?????4?0????
0???????1???????0?0?1??????111?1??????????2???????21??0?0?????0????????0??0
10????1?1??0?????????0???????0??????????????????113????0???420??1220000 1??0
0?10121???2??12?????11??????????????????????
Zalambdalestes 11?1?11[0 2]1000[1 2]401?1?110120[0
1]021110030120011 1000??00?1112211?021220120?00120?0????121011120?1012?
?????0120121002110?011022204201411000001011 0211?11?002011011000000000?
?000112012 1??2211121000101 10000[2 3]00211?00001010[1 2][1 2]33[2
3]10010200?1111????????????002??1110010?110??0???01?221011110?2111011120
0?2210111000010211122000 00001?10110?2111210??1221000111223?201013?011
300000000002000001??00111003122112210022311125?1??21011010?024112001 0
13???11101102010001100110011111???????21?2??????32130020110200000201?10
11010111101110[0
1]20021011 0001?1010000??1011223???????113????0???420??12200001??[1
2]0?1112?1??2??121????11???????????????????????

Daulestes

11?1?11210002401?1?11012000211100301200111000??00????1??1??????0120
????00?0????121011120?1012??????0020111002110?011?112021114110001110100
211?11?011010011111010000??0001120121??2211121000101110000?00100??00000
1011332000?0??0?1000???????????002???
??
???????????????1100212011201002231112511???10???????2?????01013??????11
?201100??001???????????????2??1??????3???00?01?1??000?100?10??000?1111?11
0[0
1]20????1??00???????00??2??????????????113????0???420??12200001??00????????
??????1?????1??????????????????????

Aspanlestes

?????1?????0??0???????????1????????0???0?1?0?0??00?11112?1?1?010?100?00
0?0?0????12101113101012??????0110111002110?011?1120121141100000101 0021
1?11?0221110312100?0000??100112012 1??221112100010110000???????????????
033?0???0????1??0???????????002???
??
?????????????????????????????3?????????1?0????10????0?????1??????????????00?
1?????????1?????????????1?????????????????????0?????????????????????????????
???????????????????????????113????0???420??1220000???00???????????????????
??????????????????????

Eoungulatum

?????1???????????????????1????????0???0?1?????????10110?1???????100?????
??0????121011141110112??????0110121002110?011?112012114110100010200311?
11?2221110312100100000??10011200????22111210001 0110000????????????????13
32????0????1??0???????????002???
??
???????????????????????????3??????????1??????10????0??????????????????000???
????????1?????????????1???
?????????????????????????1?3????0???420??12200001??00??????????????????????
?????????????????????

Cimolestes

1??1?10?10?02401?1?1101200?2111003012?0111000??00?10122?1?110100120

?00010?0????12101113101012??????0020111002110?011221203111411000111020
0211011?10101001111000000 0??0001120121??2211121000101100 00???200??00?0
000113321??00????1??0??????????2002??
???
???
0??
??????????????????????????????113????0???420??12200001??00???????????????
??????????????????????????

Gypsonictops

1??1?10?10002401?1?1101200?2111003012?0111000??00?11122?1?121200120
?00110?0????12101113101012??????0120111002110?011221204111411000111020
0211011?10101001111000000 0??0001120121??2211121000101100 00???????00?0
000113320??00????1??0??????????2002??
???
???
???
??????????????????????????????113????0???420??12200001??00???????????????
??????????????????????????

Protungulatum

11?1?10?10002401?1?1101200021110030120011 1000??00?10120?1?12110010
0?00010?0????12101114111012??????0110111002110?0112112012114110100 0102
01311?11?2221110310000000 00??10011200?1??2211121000101100 00???????00?
0000113321??00????1000??????????2002??????????????????????????????????????
??222003?11122312?1013011132
011??????????????????????????????????2311125?1??1?0????0?0??[0
1]0?????1?????????????11001????1??111????????????2?????????????????????????
??????????????????????1???????????????????????????????113????0???420??122000
01???0???

Erinaceus

11?1?11210002411?1?1101200021110030130011 1000??00?1011011?12122002
0?00100?0????121011120?1012??????0110121002110?01101120221141101000102
11211?11?112111020??0000000??10033300?1??2211121011101100002103000 0010
00103333420000200111 10??????????20021111100102110 1200?11112[1

2]1011110121110111200?2210111000010211122100000111011102101210?12220
0301112251201013011132011000000200100112001110031211120100223111 25111
121011010?023113001113???1110110201100110011001111131 20?1121220111?14
2130021111300000200110011011111 1?11?2210210111001?11100201220112231 11
0111113????0??1420??12200001??0011112011 12??1014???11?????111010110100?
0011
Leptictis
11?1?11210002401?1?1101200021110030130011 1000??00?1112211?12122002
0?00100?0????121011121110 12??????01101210021 10?01121120411141100000101
01211?11?101110011110000000??10011200?1??22111210111111000021020 00000
0001011332100002 00?1000???????????002??????010?1101200?101?22??111?0121
110111200?2210111?????02111221?00000111011102211210?1222003?1112231201
0130111320110 0???0200?001???01110031211120100223111251111 11011010?0231
13001113???1110110201100110011001111 03?20???21?20?????3213012111120000
030001001101111111?1110200210111001?101000012201 1223???????113????0??14
20??12200001??00110110?11???1214???11??????????????????????
Canis
1111?11210102401?1?11011000211101301300111000??00?0100011?00110100
0?00010?1????121011130?1011??????00010?1000000?00?000000101110??0001021
000??00101021101100011 0000??31011210?1??21111000?01110000020020 0000000
000233231000000001000??????????20121111110102110120??1011212011 1101211
11111200?2211111000000211 1221?000001100110?1111110?22212020111 2251001
0130111?30110?0?0020000011100111003021113110022311125111?11?101?0??231
11101113?????10110200001000 0110?1012[0
1]3120?1121220111?1421311201114010013000100110111110101 0220022 1210001
?1111100122001223111011 1113????0??1420??1??????1??001111101112??1214???
11?????1111101100?0?0011
Felis
1111?11210102401?1?11011000211101301300111010??00?0100011?00110100
0?00010?1????121011130?1011??????00010?1000000?00?000000001110??0001021
000??001010211000?00110000??31011210?1??21111000?01?100???200200000000
00034434300?000001000??????????20121111110102110120??1011212011 1101211
11111200?2211111000000211 1221?000001100110?1111110?22212020111 2251001

0130111?30110?0?0020000011100111003021113110022311125111?11?101?0??231
11101113?????10110200001000011 0?101203120?1121220111?1421311201114010 0
13000100110111110101022002212100 01?1111100122001223111011111 3????0??1
420??1??????1??0011111011 12??1214???11?????1111101100?0?0011
Rattus
1111?1101000241 1?1?1101120001 1212?0110002101 0???????????0???????????
??????0????121?????11101 2??????413?0?001?000?00?0????4201311000001 03?140
???1?0122110 20?????????????0033100????2??????????0?1?0???31?411111???1??333
343????1?021112???????????2002111110010211 01200?1011221011100011101 0002
10?2211110000010211121 1?0010111101110211 1100?122100301112250001011011
?31011010100010000111001110020101100110123111251111 11010000?024110101
1131??2?1011010100111002111101103120?1121220111?142130020111212001400
110011001111 1?0112211?21210001?121?2001211112231110111111 3????3??14?0??
???????1??2?1111101112??1114???11?????1111101100?100000
Oryctolagus
1111?1101000240 1?1?110111000 11212?011?1121010??00?0012000?00100000
0?001???0????121?????0?1012??????413?0?001?000?00?0????2101410??000??3?1
30???1?002111030?????????????0133200????2??????????0?1?0???31?411111???1??3
33343???0??021112???????????211311111001021101201?10112210111000 2000101
1200?22101100000102111201?0010111101110210111 0?0?21113011122500010130
10131011010100010 00?111001110020101100110?23111251111 11010000?0241101
011131??2?101101000011100111010 1103120?1121220111?152130000111 1121003
0011?1?11111111?0112211?21210001?121?20012111122311101111 13????3??14?0
?????????1??1?1011101112??1114???11?????1111111100?113?00
Bradypus
1111?112101024 11?1?1101100021110130 13?0111000???????????0??????0000?
??????0??????????????1014??????5??????????????0?3??????????????????????????
??????????????????????05540??????????????????????5??5????01??1??????00????
???01??????????????301?1111110102111120??1011212011121021111111200?22111
1100000021113 21?110001100110?1201100?1221201011122510010130111?30110?
0?0020000?1110011100302111311002231112511 1?11?101?0??23021101013?????1
011020000[0
1]0000110?101203120?1121220111?1421311301111030010100 1?01102111101011

220??21210001?1011001122001223111011100?????4??14?0?????????1????11?110?
112??0[0 1]14???11?????1110111010113111

Tamandua

1111?11210102411?1????????0?1110?30???1??10?0????????????????????????
????0????????????????0??
???5?05????01??1???????????????0
1?????????????3???1111110102111120??101121201112102111101200?221111100
00002111321?110001100110?1101100?2221201011122510010130??1?30110?0?002
0000?11100111003121113110022311125111?11?101?0??231?1101013????101102
001000000110?101203120?1121220111?1421311301111030010100 2?01102111101
011220??21210001?101100112200122311101111?????????14?0?????????1????11?1
101112??0?14???11?????1110111010113111

Glyptotherium

1111?11210102411?1?1101100021110130131011100???????????0??????0000?
??????0??????????????1014??????5??????????????0?3??????????????????????????
??????????????????????03330??????????????????????5?05????010?1??????00????
???01?????????????301011111010211 1120??101121201112002111101200?22111
110000002111321?110001100110?1201210?2221201011122510010130111?30110?
0?0020000?11100111103021113110022311125111?11?101?0??230?1101013?????1
011020000?0000110?10120?????11??220111?14213113011110300101001001 10211
111?111220??21210101?1011?01122???22311??11100?????4??14?0?????????1????
11?1101112????14???11?????????111100?113???

Dasypus

1111?11210102411?1?1101100021110130131011100???????????0??????0000?
??????0??????????????1014??????5??????????????0?3??????????????????????????
??????????????????????05540??????????????????????5?05????010?1??????43????
???01?????????????3010111111010211 1120??101121201112002111101200?22111
110000002111321?110001100111011 01210?2221202011122510010130111?30110?
0?0020000?11100111103021113110022311125111?11?101?0??230[1
2]1101013?????10110200000000110?101203120?1121220111?142131130111 1130
0101002001101111 11?111220??21210101?1011001122001223111011100?????4??1
4?0?????????1????11?120?1?2???[0 1]14???11?????1110111100?113111

Chaetophractus

1111?11210102411?1?11011000211101301310111100???????????0??????0000?
??????0??????????????1014??????5??????????????0?3???????????????????????????
???????????????????????05540???????????????????????5?05????010?1??????43????
???01?????????????30101111110102111120??1011212011120021111101200?22111
1100000002111321?110001100111011012102?22212020111225100101301111?30110?
0?0020000?11100111113021113110022311125111?11?101?0??23021101013?????1
011020000[0 1]0000110?1012[0
1]312??112122011 1?142131130111113001010020011021 1111?111220??21210101?
1011001122001223111011100?????4??14?0?????????1???11?110?112???14???11
?????1110111100?113111

Euphractus

11?1?11210102411?1?11011000211101301310111100???????????0??????0000?
??????0??????????????1014??????5??????????????0?3???????????????????????????
???????????????????????05540????????????????0??????5?05????010?1??????43????
???01?????????????30101111110102111120??1011212011120021111101200?22111
1100000002111321?110001100111011012102?22212020111225100101301111?30110?
0?0020000?11100111113021113110022311125111?11?101?0??23021101013?????1
011020000[0 1]0000110?1012[0
1]3120?112122011 1?142131130111113001010020011021 1111?111220??21210101?
1011001122001223111011100?????4??14?0?????????1???11?110?112???14???11
?????1110111100?113111

Holoclemensia

??????????????????????????????????????0?1???0??????????????????110???????
??????121011110?1012??????002010100211 0?011111102111411000??11100111?21
?001110001001001111111100112?121??221112100010000000????????????????????
????0???????????????????002???
??????0??
???
???
?????????????????????113????0???420??1220000????0???????????????????????????
????????????????

Deltatheridium

11?1?11310102201?1?11011000411100301300111000??00?0000111?00010110 0?00000?0????121011110?1012??????000010100[0 2]110?0111111021114110000010100111121?000110001001001001002201120121? ?221112100010000000100100??000000022222111100?121000??????????2002????? ?? ?????12??????????2??????110212???00?????????????????11100?020112????231112 510??11010001112312???1013???2?1011?2?00001???1101101 1????????2??1?????? ?????02?11?2?000?201010??0001111????0[0 1]201?1????001??011000?????????????????113????0???420??12200001??00???????1 1????21????1????????????????????????

Atokatheridium

????????????2???????????????????????0????????00?????????????????????? ??????121011110?1012??????000010100 0110?011?1110211141 1000??0???0111?21 ?00011000100100100100220112?12???22111210001000000 0???????????????????? ?????????????????????002??? ?? ?? ?? ????????????????????113????0???420??12200001??????????????????????????????? ???????????????

Sulestes

11?1011310102201?1?11011001411??1?013101110?????0?000011??000101100 ?00000?0????121011110?1012??????00001110001 10?0111111121114110001111 21 1211121?000110001001001111002201120121??221112100010010000???????????? ??0222221??10????1??????????????002?? ?? ?????????????????????????????????231112510??1?0??????12?1???????????????????? 000??????1?1???0?2????? ??????????????0??????????????????113????0???420??122?0001???0????????????????? ???????????????????????????

Asiatherium

11?1?11310102401?1?11011001411??0?01300111000??00?0000111?00010110

0?00000?0????121011120?1012??????0010121002110?01101111221141100111112
11211021?0111111211?0001001001001120121??221112100010110000??0?????000
0??02222210010?????00????????????002????100102110??0????11231011110?2111
0111200?221111111110?21012200000001?10110?1100100?01??????????2????????
????????????????????00??001110?30201120100?2311125????1111000???23??4101
013?????10?1?2??0?0101111????11????????2??????????????02011?2?00??21??10??
0?0?11?????0[0
1]20????11000???????10??2?????????????113????0???420??12200101???0?1?1????
????021????1??????????????????????
Kokopellia ?1?1?11310?022???1?1??1100?[3
4]11??????3?0111000?????????1???0001011???00000?0????121011110?1012??????
0010111002110?0110011122114110011111210211?21?0101100111?100111100100
1120121??221112100010110000???1????00?0000?222?1??10??0?1000??????????20
02??
???0?????????????????????????
???
??1?3?
???0???420??122?000???00???
Anchistodelphys
????????????????????????????????????0?????????????????????????1????????
?0????121011110?1012??????0020121002110?011?01112201411001111221021112
1?012121021010101111002001120?????22111110001?110000?????????????????????
????????????????????????002???
???
???
???
??????????????????????1?3????0???420??12200101??????????????????????????????
????????????????
Albertatherium
????????????????????????????????????0?????????????????????????1????????
?0?????21011120?1012??????0???????????????1???1????????????????????11121?01
221102101100111101200??2?121??22111110???0???000?????????????????????2??????
????????????????????002???

??
??
??
?????????????????3????0???420??122?0?01??????????????????????????????????
??????????

Didelphodon

11?1?11310102101?1?1101100141110130 1?00111000??00?001???1?000110100
?00000?0????121011120?1012??????000012100211 0?01121111220541 1101??1221
1211121?012221020?0100201100210112012 1??2211111000101 10000??0???????0?
000222221??00????1??0??????????2002??????????????????????????????????????
??21201?100123110101102122 10
11???????????????????10?3020112010022311 12??0????1???0??1??11?????1??????1
?????0?00?1?????01101??????????1?1???????????0????????????????????????????
?0????1????0????????1????????????????113????0???420??122?0101???0????????
?????????????????????????????????

Pediomys

???1?11310102101?1?1101100141110130 1?00111000??00???????1???????100?
????0?0????121011120?1012??????00101210021 10?0111111122014111011112 2112
11121?01222102110000111100100112012 1??2211111000101 10000???????????00
02222211100???????0??????????2002???
???21201?100124220201102122 1011
?????????????????????????????????123111???0????0???0??1???1?????1??????1????
???00?1?????01101?????????????1??????????????????2???0????????????????????????
??1??????????????1?????????????????113????0???420??12200101???0?????????????
??????????????????????????????

Turgidodon

???1?11310102101?1?1101100?4??101301?00111000??00???????1?00010?100?
00000?0????121011110?1012??????001012100211 0?011111113211411101111 2211
211121?0122110211000011110020011201 21??2211111000101 10000???????????0
00222221??00???21??0??????????2002???
???2?????????
??????????????????????????????223111???0????0???0??1??11?????1??????1??????
?00?1?????0?101?????????????1??

1?????????????????????????????????3????0???420??122?0101???0????????????????
??????????????????????????

Mayulestes

11?1?113101024?1?1?110110014111003013001110000??00?0000111?00010110
0?00000?0????121011110?1012??????00101210021 10?0111111122114111011112 1
10211121?01222002110000101100210112012 1??221111100010110000000 1000000
000002222211100111?1000???????????0021111 0010?1101201?1011231011 11012
11111 11200?2211111?11??0211122000000011 10110?1110000?012?????????24220
201102121 1011??????210???0??00111003020112010112311125???1101000???241
10101013?????10110210010100111111011 1???????21?????????????020110200000
21101001000?11001010020???0110001?1111100??2?????????????113????0??1420?
?122?0101??00?10110?112??021????11??????????????????????

Pucadelphys

11?1?1131010240 1?1?110110014111003013001110000??00?0000111?00010110
0?00000?0????121011110?1012??????012012100211 0?0110111122114111011112 2
10211021?01222112111000211101100112012 1??221111100010110000000 1000000
0000022222111101112 1000???????????00211111001021101201?1011231011 11012
11111 11210?221111??????0211122000000011 10110?1110000?012130101001 24220
201102122101 1?101?0210???0??0011100302011201011231112511??110100011124
110101013?????10110210010100111111011 1???????21?1??????2????02011?20000
02110100111011100101002 01?10110001?1011100?21?????????????113????0??142
0??12200101??00?101101112??021????11??????????????????????

Andinodelphys

11?1?1131010240 1?1?110110014111003013001110000??00?0000111?00010110
0?00000?0????121011110?1012??????012012100211 0?0112111122114111010012 2
11211021?01222112111000211101100112012 1??221111100010110000000 1000000
00000222221111 0111?1000???????????002??????0????????0???0????????????????
??????????????????????????????????????0???????????????????2???????????????
??????????????0????111003?201120101223111 25????11010001 1?241101 01013????
?10110210010100011111011????????21?1???????????02?111?1000021?010011001
11001?100201?10110001?101111??21?????????????113????0??1420??122?0101??0
0????????????????????11??????????????????????

Didelphis

11?1?11310102411?1?11011001411100301300111000??00?0000211?00010110
0?00000?0????121011110?1012??????0020121002110?01101111221141110100122
11211021?012221120?00002111012001120121??2211111000101100000001000000
000002222211110111121000??????????2002111110010?1101201?101123101111012
11111112[0
1]0?221111121110021112200000001110110?1110000001213010100124220211102
123201111011021001001100111003020112010112311125111111110001112311[0
1]101013?????1011020000010111101101113120?1121210111?02212002011121000
0210010011001110010100201?10010001?11111101210112230011111113????0??14
20??12200101??001101201112??0214???11?????101111113110?2000

Marmosa

11?1?11310102411?1?11011001411100301300111000??00?0000211?00010110
0?00000?0????121011110?1012??????0020121002110?01111111211141110100122
12211021?012221120?00002111011001120121??2211111000101100000001000000
00010222221000011121000??????????00211111001021101201?101123101111012
1111111200?221111121110021112200000001110110?111000000121301010012422
021110212220111101102100100110011100302011201011231112511111111000111
23111101013?????1011020000010111101101113120?1121210111?0221200201112
10000210010011011110010100201?11211001?111111012101122300111111113????
0??1420??12200101??001101101112??0214???11?????1011?????????????

Caenolestes

11?1?113101024?1?1?11011001411100301300111000??00???????1??????0100?
????0?0?????21011110?1012??????0020121002110?01111111321141110100122122
11021?012221120?00102101001001120121??22111110001011?0001001000000000
00222221001002021000???????0???00211111001021101201?101123101111012111
1111200?221111121110021112200000001110110?1110000?01213020??012422010
11021221011110000210100011001110030201120??1123111251111111301011?231
10101013?????10?1020000000211101101113120???21110111?03212002011131000
021?010011?01110010100201?2??10001?111?220121???22300111111113????0??14
20??12200101??001101101112??0014???11?????1011?????????????

Dasyurus 11?1?11310102401?1?11011001411100301300111000??00?00[0
1]0211?000101100?00000?0????121011110?1012??????0020121002110?011111112

21141110100122122111121?012221120?0010210100200112012 1??22111110001011
000010020000000000033223111000112100 0??????????0021111100102110120 1?1
0112310111101211111112 00?22111112111002111220000000 1110110?1110000?01
2131201001251001012121 3?1011110000210100011001110030201120101323 11125
11111113010111231011010 13?????10110200000102111011012 1?120???21110111?
04212002011111200021101001101111001010020 1?20110001?1111110121011 2230
011111113????0??1420??12200101??001101101112??0214???11?????1011?111211
0?2000
Perameles
11?1?1131010241 1?1?1101100141110030130011 1000??00??????????????0000?
????0?0?????21011110?1012??????0020111002110?011211113211411100001 22122
11021?012221120?001021010020011201 21??22111110001011?00000020000000 00
00222221001002121001???????????0021111100102110120 1?1011231011110121 11
1111200?2211111211100211122000000 001110110?1100100?01213020??112510020
121213?10111101002001000110111100302011201111231112511111113 01011?231
14101013?????101102000001021110110111312 0???21110111?0421200201112 1000
0210010011011110010100201?21210001?111011012 1???2230010111113????0??14
20??12200101??001101101112??0?14???11?????1011111121 10?2000
Dromiciops
1111?11310102401?1?110110014111003013001110 00??00?000021??00010000
0?00000?0????121011110?1012??????002012100211 0?0111011132014111 0100122
12211121?012221120?00000001011001120121??22111110001?1100000001000000
00010222221001002121000???????????002111110010211 01201?1011231011110 12
1111111200?22111112111002111220000000 1110110?1110000?01213020??112510
020121213?2011110100210010011001110030201120101123111251111111301011?
231?4101013?????10110200000102111001011031 20???21220111?0321300201 1111
000031101001100111 0??0100201?21211001?1011110121011223001111111 3????0?
?1420??12200101??001101101112??0?14???11?????1011?????????????
Thylacomyidae
1111?113101024?1?1?1101100141110030130011 1000??00???????1??????1000
?????0?0?????2101???????1???????4???????????0?011??11132???1??????12???4???
?1?032??1120?001000010??011120121??22111010????110000000 20000000????222
221???0?2?2100????????????0021111100102110120 1?1011231011110121 11?11120

0?22111111111100211122000000001110110?1110000?012130?0????2510020121213?
??11??0?002?0?00011011110030 2?112?10132311125111111130101 1?2310?101013
?????101102?0000102?1???101??3120???21??0?11?0421?0020111?1000021?01001
1?0111?0101?020??2??1?001?1?1???0121???22300??111113????3??1420??1220010
1??001101101112??0?14???11?????1011?????????????
Macropus
1111?11310102401?1?110110014111003013001110 00??00??????????????0000
?????0?0?????21?1115121012??????41301200020 00?0113301132013112100013312
40??31?03221112 0?00100000??3001120121??2211101011 0?110???2004000001???
??332232??1102021112????1?10???0021111100102110120 1?101123101111012111
1111200?221111111111002111220010000111011 0?1210010?01213120??112510010
121213?1011110100200000011011110030211131120323111251111111301011?231
14101013?????101102000001031110010111312 0???21220111?0521300201111110 0
1311010011001110010100301?21211001?101?0[0 1
2]0121???2230011111113????3??1420??12200101??001101101112??0?14???11????
?101111112110?2000
Acrobates
1111?11310102411?1?110110014111003013001110 00??00??????????????0000
?????0?0?????21?1015121012??????01301110020 00?01101011321141110000 13212
411?31?0322111 20?00100001011001120121??2211101000 1?110???0003000000000
1022223100000202111 0????????????0021111100102110120 1?101123101111012111
1111200?22111111111100211122000000001110110?1110000?01213020??112510020
121213?2?111101002?0?1?011011110030211131120323111251111111301011?231
14101013?????101102000001011110110120312 0???212?1111?0521310201113100 0
031101001100111001010020 1?21211001?1111120121???223001111111 3????0??14
20??12200101??001101101112??0?14???11?????1011?????????????
Phascolarctos
1111?11210102401?1?110110014111003013001100 00??00??????????????0000
?????0?0?????21?1015121012??????41301220020 00?0113301132013112000013312
411?31?03221112 0?00100100??3011120121??2211101011 0?110???20040000010??
??332233??200102111 1????????????0021111100102110120 1?101123101111012111
1111200?221111111111002111220010000111011 0?1110000?01213020??112510020
121213?1011110100210 01?011011210030211100100323111251111111301011?231

14101013?????1011020000010211101101113120???2112011 1?03213002011131100
14100100111011100101003 01?20110001?1111020121???2230011111113????3??14
20??12200101??001101101112??0?14???11?????1011?????????????
Vombatus
1111?11310102401?1?11011001411100301300110000??00??????????????0000
?????0?0?????21?1015121012??????4130122002000?0113301132013112100013312
411?31?022211120?00100000??3011120121??22111010110?110???30040 1011?0??
??332233??201?021112???????????0021111100102110120 1?10112310111101 21111
111200?2211111111100211122001000011101 10?1100100?01213020??1125100201
21213?1011110100210010011011210030211 10?0?0323111251111111301011?2302
3101013?????1011020000010111100101113120???2112011 1?05213002011131000 1
411010011001110010100301?20210001?1111001121???2230011111113????3??142
0??12200101??001101101112??0?14???11?????1011?????????????
Phalanger
1111?11310102401?1?11011001411100301300111000??00??????????????0000
?????0?0?????21?1015121012??????0130122002000?0113300132114111000013312
411?31?032211120?00100100??300112012 1??22111010110?110???2003000000000
1022222200210202 1111???????????00211111001021101201?1011231011110121 11
1111200?2211111111100211122000000001110110?1110000?01213020??112510020
121213?2011110100210010011011210030211131120323111251111111301011?231
14101013?????1011020000010311100101113120???2122111 1?05213102011131100
131001001100111001010030 1?20210001?1111120121???2230011111113????0??14
20??12200101??001101101112??0?14???11?????1011?????????????
Pseudocheirus
1111?11310102401?1?11011001411100301300111000??00??????????????0000
???????0?????21?1015121012??????0130122002000?0113300132013112000013212
411?31?032211120?00100100??3011120121??22111010110?110???2003000001000
103322320000020211 11???????????00211111001021101201?1011231011110121 11
1111200?2211111111110?211122000000001110110?1110000?01213020??112510020
121213?2011110100210010011011210030211131120323111251111111301011?231
14101013?????1011020000010311100101213120???2122111 1?05213102011121000
031101001100111001010120 1?21210001?1111120121???2230011111113????0??14
20??12200101??001101101112??0?14???11?????1011?????????????

Petauroides

1111?11310102401?1?110110014111003013001110000??00??????????????0?00?
??????0?????21?1015121012??????0130122002000?01133001320131120000132124
11?31?032211120?00100100??3001120121??221110101100110???20030000000001
0332232000002021111???????????00211111001021101201?101123101110121111
111200?22111111111100211122000000001110110?1110000?01213120??1125100201
21213?20111101002100100110112100302111311203231112511111113010111?2311
4101013?????10110200000103111001012131200???21221111?0521310201112100000
211010011001110010101201?21211001?1011120121???22300111111113????0??142
0??12200101??00110110111112??0?14???11?????1011?????????????

Supplementary References

1. Allin EF. 1975. Evolution of the mammalian middle ear. J Morphol 147:403-437.
2. Allin EF, Hopson J. 1992. Evolution of the auditory system in Synapsida "mammal-like reptiles" and primitive mammals) as seen in the fossil record. In: Webster DB, Fay RR, Popper AN, editors. The Evolutionary Biology of Hearing. New York: Springer-Verlag. p 587-614.
3. Anthwal N, Urban DJ, Luo ZX, Sears KE, Tucker AS. 2017. Meckel's cartilage breakdown offers clues to mammalian middle ear evolution. Nat Ecol Evol 1:93.
4. Bi S, Zheng X, Wang X, Cignetti NE, Yang S, Wible JR. 2018. An Early Cretaceous eutherian and the placental–marsupial dichotomy. Nature 558:390-395.
5. Cifelli RL. 1999. Tribosphenic mammal from the North American early Cretaceous. Nature 401:363-366.
6. Goloboff P, Catalano SA. 2016. TNT version 1.5, including a full implementation of phylogenetic morphometrics. Cladistics 32:221-238.
7. Goloboff PA. 1999. Analyzing large data sets in reasonable times: solutions for composite optima. Cladistics 15:415-428.
8. Graboyes EM, Chole RA, Hullar TE. 2011. The ossicle of Paaw. Otol Neurotol 32:1185-1188.
9. Han G, Mao F, Bi S, Wang Y, Meng J. 2017. A Jurassic gliding euharamiyidan mammal with an ear of five auditory bones. Nature 551:451-456.
10. Han G, Meng J. 2016. A new spalacolestine mammal from the Early Cretaceous Jehol Biota and implications for the morphology, phylogeny, and palaeobiology of Laurasian ‘symmetrodontans’. Zool J Linn Soc-Lond 178:343-380.
11. Harper T, Rougier GW. 2019. Petrosal morphology and cochlear function in Mesozoic stem therians. Plos One 14:e0209457.
12. He HY, Wang XL, Zhou ZH, Wang F, Boven A, Shi GH, Zhu RX. 2004. Timing of the Jiufotang Formation (Jehol Group) in Liaoning, northeastern China, and its implications. Geophysical Research Letters 31:1-4.
13. Ji Q, Luo ZX, Yuan CX, Wible JR, Zhang JP, Georgi JA. 2002. The earliest known eutherian mammal. Nature 416:816-822.
14. Ji Q, Luo ZX, Zhang XL, Yuan CX, Xu L. 2009. Evolutionary development of the middle ear in Mesozoic therian mammals. Science 326:278-281.

15. Kermack KA, Mussett F, Rigney HW. 1981. The skull of *Morganucodon*. Zool J Linn Soc-Lond 71:1-158.
16. Kielan-Jaworowska Z, Cifelli RL, Luo ZX. 2004. Mammals from the age of dinosaurs: origins, evolution, and structure. New York: Columbia University Press.
17. Krause DW, Hoffmann S, Hu Y, Wible JR, Rougier GW, Kirk EC, Groenke JR, Rogers RR, Rossie JB, Schultz JA, Evans AR, von Koenigswald W, Rahantarisoa LJ. 2020. Skeleton of a Cretaceous mammal from Madagascar reflects long-term insularity. Nature 581:421-427.
18. Kusuhashi N, Tsutsumi Y, Saegusa H, Horie K, Ikeda T, Yokoyama K, Shiraishi K. 2013. A new Early Cretaceous eutherian mammal from the Sasayama Group, Hyogo, Japan. Proceedings of the Royal Society B: Biological Sciences 280:20130142.
19. Lillegraven JA, Krusat G. 1991. Cranio-mandibular anatomy of *Haldanodon exspectatus* (Docodonta; Mammalia) from the Late Jurassic of Portugal and its implications to the evolution of mammalian characters. Contributions to Geology, University of Wyoming 28:39-138.
20. Lopatin AV, Averianov AO. 2018. The stem placental mammal *Prokennalestes* from the Early Cretaceous of Mongolia. Paleontol J+ 51:1293-1374.
21. Luo Z-X, Manley GA. 2020. Origins and early evolution of mammalian ears and hearing function. In: Fritzsch B, editor. The senses: a comprehensive reference. Cambridge, Massachusetts: Elsevier Academic Press. p 207-252.
22. Luo ZX. 2007. Transformation and diversification in early mammal evolution. Nature 450:1011-1019.
23. Luo ZX. 2011. Developmental Patterns in Mesozoic Evolution of Mammal Ears. Annual Review of Ecology, Evolution, and Systematics 42:355-380.
24. Luo ZX, Ji Q, Wible JR, Yuan CX. 2003. An early Cretaceous tribosphenic mammal and metatherian evolution. Science 302:1934-1940.
25. Luo ZX, Meng QJ, Grossnickle DM, Liu D, Neander AI, Zhang YG, Ji Q. 2017. New evidence for mammaliaform ear evolution and feeding adaptation in a Jurassic ecosystem. Nature 548:326-329.
26. Luo ZX, Yuan CX, Meng QJ, Ji Q. 2011. A Jurassic eutherian mammal and divergence of marsupials and placentals. Nature 476:442-445.
27. Mao F, Hu Y, Li C, Wang Y, Chase MH, Smith AK, Meng J. 2020a. Integrated

hearing and chewing modules decoupled in a Cretaceous stem therian mammal. Science 367:305-308.

28. Mao F, Liu C, Chase MH, Smith AK, Meng J. 2020b. Exploring ancestral phenotypes and evolutionary development of the mammalian middle ear based on Early Cretaceous Jehol mammals. National Science Review 8:1-10.
29. Mao F, Zhang C, Liu C, Meng J. 2021. Fossoriality and evolutionary development in two Cretaceous mammaliamorphs. Nature 592: 577-582.
30. Mckenna MC, Kielan-Jaworowska Z, Meng J. 2000. Earliest eutherian mammal skull from the Late Cretaceous (Coniacian) of Uzbekistan. Acta Palaeontol Pol 45:1-54.
31. Meng J. 2014. Mesozoic mammals of China: implications for phylogeny and early evolution of mammals. National Science Review 1:521-542.
32. Meng J, Bi S, Zheng X, Wang X. 2018. Ear ossicle morphology of the Jurassic euharamiyidan *Arboroharamiya* and evolution of mammalian middle ear. J Morphol 279:441-457.
33. Meng J, Hu YM, Wang YQ, Li CK. 2003. The ossified Meckel's cartilage and internal groove in Mesozoic mammaliaforms: implications to origin of the definitive mammalian middle ear. Zool J Linn Soc-Lond 138:431-448.
34. Meng J, Mao F. 2021. Monotreme middle ear is not primitive for Mammalia. National Science Review 8: nwab131.
35. Meng J, Mao F, Han G, Zheng X-T, Wang X-L, Wang Y. 2019. A comparative study on auditory and hyoid bones of Jurassic euharamiyidans and contrasting evidence for mammalian middle ear evolution. J Anat 236: 50-71.
36. Meng J, Wang Y, Li C. 2011. Transitional mammalian middle ear from a new Cretaceous Jehol eutriconodont. Nature 472:181-185.
37. Nixon KC. 1999. The parsimony ratchet, a new method for rapid parsimony analysis. Cladistics 15:407-414.
38. Ronquist F, Teslenko M, Van Der Mark P, Ayres DL, Darling A, Höhna S, Larget B, Liu L, Suchard MA, Huelsenbeck JP. 2012. MrBayes 3.2: efficient Bayesian phylogenetic inference and model choice across a large model space. Systematic Biol 61:539-542.
39. Sadier A, Sears KE, Womack M. 2021. Unraveling the heritage of lost traits. Journal of experimental zoology Part B, Molecular and developmental evolution 338:107-

118.

40. Sulej T, Krzesinski G, Talanda M, Wolniewicz AS, Blazejowski B, Bonde N, Gutowski P, Sienkiewicz M, Niedzwiedzki G. 2020. The earliest-known mammaliaform fossil from Greenland sheds light on origin of mammals. Proc Natl Acad Sci U S A 117:26861-26867.
41. Torres A, Goloboff PA, Catalano SA. 2021. Parsimony analysis of phylogenomic datasets (I): scripts and guidelines for using TNT (Tree Analysis using New Technology). Cladistics 61: 625-630.
42. Wang H, Hoffmann S, Wang D, Wang Y. 2022. A new mammal from the Lower Cretaceous Jehol Biota and implications for eutherian evolution. Philos T R Soc B 377: 20210042.
43. Wang H, Meng J, Wang Y. 2019. Cretaceous fossil reveals a new pattern in mammalian middle ear evolution. Nature 576:102-105.
44. Wang JY, Wible JR, Guo B, Shelley SL, Hu H, Bi S. 2021. A monotreme-like auditory apparatus in a Middle Jurassic haramiyidan. Nature 590:279-283.
45. Wang Y, Hu Y, Meng J, Li C. 2001. An ossified Meckel's cartilage in two Cretaceous mammals and origin of the mammalian middle ear. Science 294:357-361.
46. Wible JR, Hopson JA. 1995. Homologies of the prootic canal in mammals and non-mammalian cynodonts. J Vertebr Paleontol 15:331-356.
47. Wible JR, Novacek MJ, Rougier GW. 2004. New data on the skull and dentition in the Mongolian Late Cretaceous eutherian mammal *Zalambdalestes*. B Am Mus Nat Hist 281:1-144.
48. Wible JR, Rougier GW, Novacek MJ, Asher RJ. 2007. Cretaceous eutherians and Laurasian origin for placental mammals near the K/T boundary. Nature 447:1003-1006.
49. Wible JR, Rougier GW, Novacek MJ, Asher RJ. 2009. The eutherian mammal *Maelestes gobiensis* from the late Cretaceous of Mongolia and the phylogeny of Cretaceous Eutheria. Bulletin of American Museum of Natural History 327:1-123.
50. Wible JR, Shelley SL, Bi S. 2021. Response to 'Monotreme middle ear is not primitive for Mammalia'. National Science Review.
51. Yu Z, Wang M, Li Y, Deng C, He H. 2021. New geochronological constraints for the Lower Cretaceous Jiufotang Formation in Jianchang Basin, NE China, and their implications for the late Jehol Biota. Palaeogeography, Palaeoclimatology,

Palaeoecology 583:110657.
52. Zhou CF, Bhullar BS, Neander AI, Martin T, Luo ZX. 2019. New Jurassic mammaliaform sheds light on early evolution of mammal-like hyoid bones. Science 365:276-279.
53. Zhou CF, Wu SY, Martin T, Luo ZX. 2013. A Jurassic mammaliaform and the earliest mammalian evolutionary adaptations. Nature 500:163-167.
