# Supplementary material for: Middle ear innovation in Early Cretaceous eutherian mammals
Source: Nat Commun. 2023 Oct 26;14:6831. doi: 10.1038/s41467-023-42606-7 (PMC10603157; doi:10.1038/s41467-023-42606-7)
Supplement: Supplementary file 2 — Reporting Summary [file 41467_2023_42606_MOESM2_ESM.pdf]

nature portfolio

Corresponding author(s): Haibing Wang

Last updated by author(s): Sep 23, 2023

Reporting Summary

Nature Portfolio wishes to improve the reproducibility of the work that we publish. This form provides structure for consistency and transparency in reporting. For further information on Nature Portfolio policies, see our Editorial Policies and the Editorial Policy Checklist.

Statistics

For all statistical analyses, confirm that the following items are present in the figure legend, table legend, main text, or Methods section.

n/a	Confirmed	
[ ]	[x]	The exact sample size (*n*) for each experimental group/condition, given as a discrete number and unit of measurement
[x]	[ ]	A statement on whether measurements were taken from distinct samples or whether the same sample was measured repeatedly
[x]	[ ]	The statistical test(s) used AND whether they are one- or two-sided<br>*Only common tests should be described solely by name; describe more complex techniques in the Methods section.*
[x]	[ ]	A description of all covariates tested
[x]	[ ]	A description of any assumptions or corrections, such as tests of normality and adjustment for multiple comparisons
[x]	[ ]	A full description of the statistical parameters including central tendency (e.g. means) or other basic estimates (e.g. regression coefficient) AND variation (e.g. standard deviation) or associated estimates of uncertainty (e.g. confidence intervals)
[x]	[ ]	For null hypothesis testing, the test statistic (e.g. *F*, *t*, *r*) with confidence intervals, effect sizes, degrees of freedom and *P* value noted<br>*Give P values as exact values whenever suitable.*
[x]	[ ]	For Bayesian analysis, information on the choice of priors and Markov chain Monte Carlo settings
[x]	[ ]	For hierarchical and complex designs, identification of the appropriate level for tests and full reporting of outcomes
[x]	[ ]	Estimates of effect sizes (e.g. Cohen's *d*, Pearson's *r*), indicating how they were calculated

Our web collection on statistics for biologists contains articles on many of the points above.

Software and code

Policy information about availability of computer code

Data collection	Character matrix was compiled in Mesquite v. 3.7.
Data analysis	TNT v. 1.6 was used for phylogenetic analyses, and VGSTUDIO v. 3.0 was used for 3D reconstructions.

For manuscripts utilizing custom algorithms or software that are central to the research but not yet described in published literature, software must be made available to editors and reviewers. We strongly encourage code deposition in a community repository (e.g. GitHub). See the Nature Portfolio guidelines for submitting code & software for further information.

Data

Policy information about availability of data

All manuscripts must include a data availability statement. This statement should provide the following information, where applicable:

- Accession codes, unique identifiers, or web links for publicly available datasets
- A description of any restrictions on data availability
- For clinical datasets or third party data, please ensure that the statement adheres to our policy

The holotype of Microtherulum oneirodes (IVPP V24190) is housed in the collection of the Institute of Vertebrate Paleontology and Paleoanthropology (IVPP), Beijing, China. All data supporting the findings of this work (specimen, ct scan, virtual reconstructions) are available at IVPP. The data matrix for the phylogenetic analysis is available in the Supplementary Information. The original ct data can be shared on request via the Collection Department at IVPP. This published work and the nomenclatural acts it contains have been registered in ZooBank, and the Life Science Identifiers (LSID) for the new genus and species are registered with Zoobank (http://zoobank.org) with the identifiers urn:lsid:zoobank.org:act:5E0910F8-6F4B-46BF-B79C-4E0DF26EB630; urn:lsid:zoobank.org:act:EEC07F0A-

F8E5-4D16-9E3C-12A93E27E496.

Human research participants

Policy information about studies involving human research participants and Sex and Gender in Research.

Reporting on sex and gender	This is a study of fossil specimens. No sex and gender data were involved in the this study.
Population characteristics	NA
Recruitment	NA
Ethics oversight	NA

Note that full information on the approval of the study protocol must also be provided in the manuscript.

Field-specific reporting

Please select the one below that is the best fit for your research. If you are not sure, read the appropriate sections before making your selection.

- [x] Life sciences
- [ ] Behavioural & social sciences
- [ ] Ecological, evolutionary & environmental sciences

For a reference copy of the document with all sections, see nature.com/documents/nr-reporting-summary-flat.pdf

Life sciences study design

All studies must disclose on these points even when the disclosure is negative.

Sample size	Our study reports a finding of a new fossil, preserved in the main and counterpart slabs. Character matrix includes 135 taxa and 615 characters. Such comprehensive datasets are sufficient for exploring the phylogenetic position of the new taxon. Morphological comparisons with other fossil taxa and extant mammals were included in the manuscript and supplementary material where possible.
Data exclusions	We sampled both extinct and extant mammals and expanded the character list used for phylogenetic analyses.
Replication	We create a new character matrix for the phylogenetic analysis and the authors verify the reproducibility of phylogenetic results with multiple independent analyses.
Randomization	Randomizations were involved in the phylogenetic analysis with default values.
Blinding	The authors were blinded to group allocation during data analysis.

Reporting for specific materials, systems and methods

We require information from authors about some types of materials, experimental systems and methods used in many studies. Here, indicate whether each material, system or method listed is relevant to your study. If you are not sure if a list item applies to your research, read the appropriate section before selecting a response.

Materials & experimental systems

n/a	Involved in the study	
x		Antibodies
x		Eukaryotic cell lines
	x	Palaeontology and archaeology
x		Animals and other organisms
x		Clinical data
x		Dual use research of concern

Methods

n/a	Involved in the study	
x		ChIP-seq
x		Flow cytometry
x		MRI-based neuroimaging

Palaeontology and Archaeology

Specimen provenance	The specimens are currently deposited at the Institute of Vertebrate Paleontology and Paleoanthropology (IVPP), and the authors who are tenure-tracked faculty members at IVPP have permits to study the specimens.
Specimen deposition	The specimens are deposited at the Institute of Vertebrate Paleontology and Paleoanthropology, which is accessible to other researchers.
Dating methods	No new dates were provided in this study.

☒ Tick this box to confirm that the raw and calibrated dates are available in the paper or in Supplementary Information.

Ethics oversight	No ethical approval was required because this is a study of fossil material.

Note that full information on the approval of the study protocol must also be provided in the manuscript.
